# Supplementary material for: Longer durations of piperacillin/tazobactam treatment cause more prolonged alteration of colonization resistance in mice
Source: PLoS One. 2026 Jun 1;21(6):e0350031. doi: 10.1371/journal.pone.0350031 (PMC13225349; doi:10.1371/journal.pone.0350031)
Supplement: S1 Table — (DOCX) [file pone.0350031.s001.docx]

Growth in cecal contents


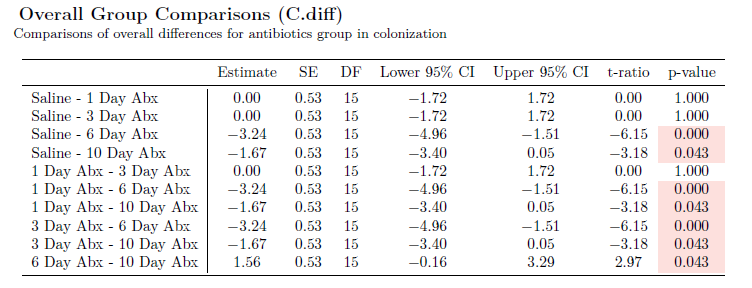


The results found that mice treated with 6 days of antibiotics showed significantly higher C.difficile colonization compared to the saline groups (*δ* = 3.24, 95% CI [1.51, 4.96]; *p* < 0.001). Comparisons between saline and 10-day group showed a statistically significant trend of increased colonization of the cecum (*δ* = 1.67, 95% CI [0.50, 3.85], *p* = 0.043). There were no significant differences between the saline control and the 1- and 3-day antibiotic groups (*p* = 0.99), indicating minimal differences in colonization pressure for shorter regimens. Both the 1 day and 3-day antibiotic groups differed significantly from the 6-day group (both *p* < 0.001) and the 10-day antibiotic group (p < 0.001). These results may indicate the C.difficile colonization is influenced by the duration of antibiotic treatment, particularly starting at 6 days of treatment, which creates a gut microbiome that is susceptible to C.difficile growth. There was an increasing trend with colonization in the 10-day groups, but the effect was not statistically significant. In contrast, the 1- and 3-day regimens appear to have less impact on colonization resistance, either preserving suppressive microbiota or allowing faster establishment of natural occurring flora.


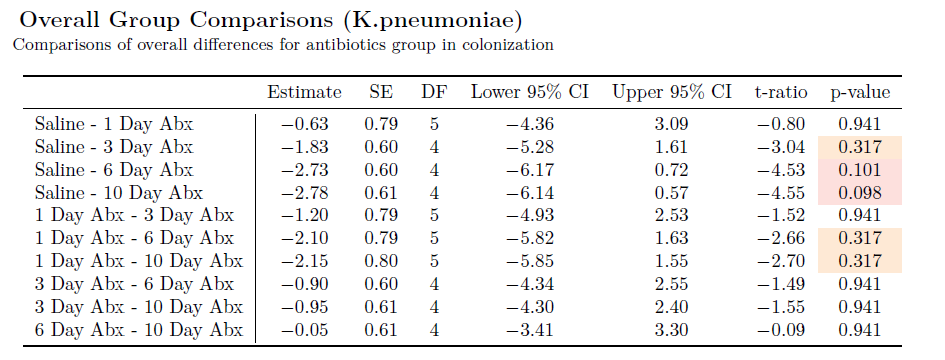


To evaluate the impact of antibiotic duration on the cecal environment ability to prevent *Klebsiella pneumoniae*, overall group comparisons of colonization levels of the cecal content were compared 0 and 24 hours after cecal harvesting. Across all comparisons, *K.pneumoniae* colonization levels tended to be higher in cecal contents from antibiotic treated groups compared to saline controls, with the highest observed differences in the 6-day (*δ* = 2.73, 95% CI [0.59, 6.15]) and 10 day group (*δ* = 2.78, 95% CI [0.58, 6.14]). These differences suggest that longer antibiotic durations produce more permissive cecal environments for *K.pneumoniae* colonization. However, none of the comparisons reached statistical significance (all *p* > 0.1)


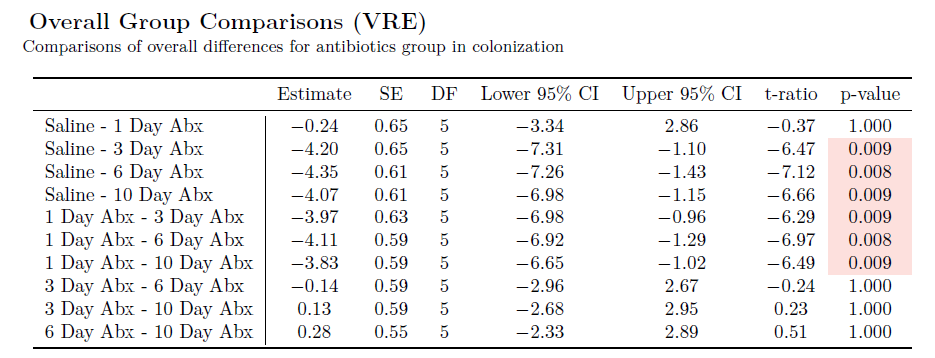


A mixed effects model was used to evaluate overall differences in VRE colonization within cecal contents of mice treated with varying durations of antibiotics. Significant differences in colonization were observed between the saline, and all multi-day antibiotic groups (3, 6, and 10 days), with consistently higher VRE burden in the antibiotic groups (all p < 0.009). In contrast, no significant differences were detected between the saline and 1 day group suggesting that brief antibiotic exposure does not disrupt the cecal environment colonization resistance to VRE. Additionally, no significant differences were found between the 3-, 6-, and 10-day regimens (p > 0.99). Collectively these finding demonstrate that longer antibiotic durations significantly reduce the suppressive capacity of the cecal environment against VRE colonization while shorter durations have minimal impact.

**Overall Summary**

Overall group comparisons revealed organism-specific responses to antibiotic duration in cecal colonization. For *C.difficile*, the 6- and 10-days treatment groups had the highest burden of colonization and significantly higher in C.*difficile* burden compared to the saline group (p < 0.043). Similarly , K.pneumoniae colonization was elevated in all groups compared to the saline control, with the highest colonization occurring within the 6- and 10-day groups, but none of the differences reached statistical significance (p > 0.09). For vancomycin resistant enterococcus, the impact of antibiotic duration was more pronounced. Mice treated with 3,6, or 10 days of antibiotics showed significantly lower colonization resistance compared to the saline group (all p < 0.009). The 1-day antibiotic group did not differ from the saline group (p > 0.99), suggesting that short-term exposure does not meaningfully compromise the suppressive capacity of the cecal environment toward VRE. No statistically significant differences were detected between the 3,6 -, and 10-day regimens, indicating a saturation effect with just 3 days of antibiotic use. These results suggest that longer antibiotic durations progressively compromise colonization resistance within the cecal, but degree of susceptibility varies by pathogen.
