# Supplementary material for: Longer durations of piperacillin/tazobactam treatment cause more prolonged alteration of colonization resistance in mice
Source: PLoS One. 2026 Jun 1;21(6):e0350031. doi: 10.1371/journal.pone.0350031 (PMC13225349; doi:10.1371/journal.pone.0350031)
Supplement: S2 Table — (PDF) [file pone.0350031.s002.pdf]

Klebsiella and VRE Day Infection 10

Samir Memic

2025-06-09

Contents

1 VRE and Klebsiella Challenge on Day 10 . . . **1**
1.1 Description . . . 1
1.2 Loading the Data . . . 1
1.3 Numerical Summary . . . 2
1.4 EDA . . . 5
1.5 Repeated Measures ANOVA (K.pneumoniae) . . . 6
1.5.1 Model Diagnostics . . . 6
1.5.2 Results . . . 8
1.6 Repeated Measures ANOVA (VRE) . . . 9
1.6.1 Model Diagnostics . . . 9
1.6.2 Results . . . 11
1.7 Linear Mixed Model (VRE) . . . 12
1.7.1 Model Diagnostics . . . 12
1.7.2 Results . . . 17
1.7.3 Conclusions . . . 19
1.8 Linear Mixed Model (*K.pneumoniae*) . . . 19
1.8.1 Model Diagnostics . . . 20
1.8.2 Results . . . 27
1.8.3 Conclusions . . . 29

1 VRE and Klebsiella Challenge on Day 10

1.1 Description

To assess the impact of antibiotic-induced microbiota disruption on susceptibility to multidrug-resistant pathogens, mice were challenged with *vancomycin-resistant Enterococcus* (VRE) and *Klebsiella pneumoniae* ten days after completing antibiotic treatment. This timeline allows for the assessment of residual microbiota perturbations and their affects on reducing colonization resistance. VRE and *K.pneumoniae* are frequently associated with healthcare-associated infections, particularly in patients recently treated with antibiotics. By challenging the recently treated mice with each of these organisms post-antibiotic exposure, this analysis aims to determine the effects of different antibiotic durations on host susceptibility.

1.2 Loading the Data

group	mice	day	organism	cfu
Saline	1	0	K.pneumoniae	2
Saline	2	0	K.pneumoniae	2
Saline	3	0	K.pneumoniae	2
Saline	4	0	K.pneumoniae	2

Saline	5	0	K.pneumoniae	2
Saline	6	0	K.pneumoniae	2

Missingness Check:

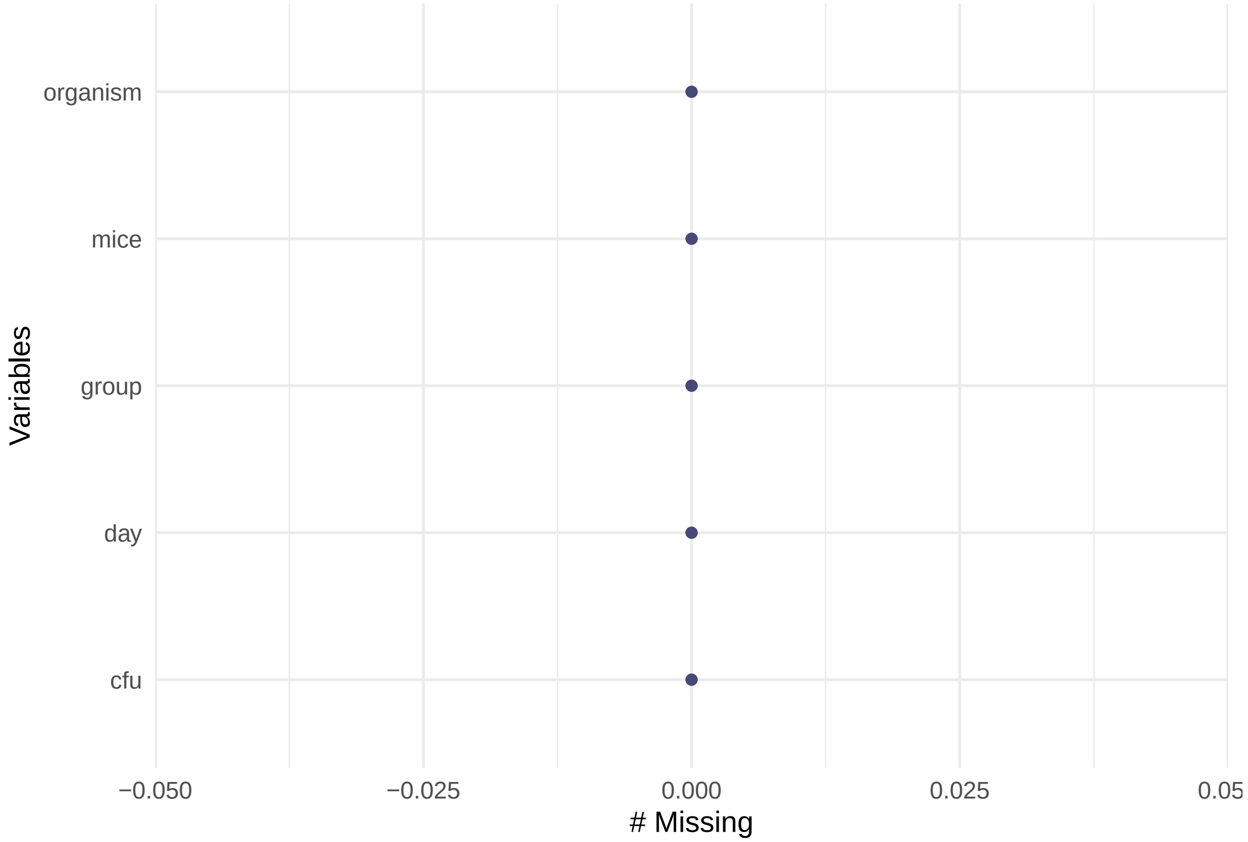

There are no missing values.

1.3 Numerical Summary

```
## fig3
##
##  5  Variables      304  Observations
## ---------------------------------------------------------------------------
## group
##        n  missing distinct
##      304        0        5
##
## Value      Saline  1 Day  3 Day  6 Day 10 Day
## Frequency      48     64     64     64     64
## Proportion  0.158  0.211  0.211  0.211  0.211
## ---------------------------------------------------------------------------
## mice
##        n  missing distinct
##      304        0       38
##
## lowest : 1  2  3  4  5 , highest: 34 35 36 37 38
```

```
## -------------------------------------------------------------------------------
## day
##        n  missing distinct
##      304        0        4
##
## Value         0    1    3    7
## Frequency    76   76   76   76
## Proportion 0.25 0.25 0.25 0.25
## -------------------------------------------------------------------------------
## organism
##        n  missing distinct
##      304        0        2
##
## Value      K.pneumoniae          VRE
## Frequency           152          152
## Proportion          0.5          0.5
## -------------------------------------------------------------------------------
## cfu
##        n  missing distinct     Info     Mean      Gmd      .05      .10
##      304        0       44    0.646    3.371     2.12    2.000    2.000
##      .25      .50      .75      .90      .95
##    2.000    2.000    4.508    7.670    7.993
##
## lowest : 2       3       3.30103 3.47712 3.69897
## highest: 9.07918 9.30103 9.47712 10.301  10.7782
## -------------------------------------------------------------------------------
```

Log_{10} Klebsiella Recovered by Antibiotic Group and Day

day	n	Mean	SD	SEM	Median	IQR	Min	Max
Saline								
0	6	2.00	0.00	0.00	2.00	0.00	2.00	2.00
1	6	2.00	0.00	0.00	2.00	0.00	2.00	2.00
3	6	2.00	0.00	0.00	2.00	0.00	2.00	2.00
7	6	2.00	0.00	0.00	2.00	0.00	2.00	2.00
1 Day								
0	8	2.00	0.00	0.00	2.00	0.00	2.00	2.00
1	8	2.00	0.00	0.00	2.00	0.00	2.00	2.00
3	8	2.00	0.00	0.00	2.00	0.00	2.00	2.00
7	8	2.12	0.35	0.12	2.00	0.00	2.00	3.00
3 Day								
0	8	2.00	0.00	0.00	2.00	0.00	2.00	2.00
1	8	2.56	1.08	0.38	2.00	0.42	2.00	4.78
3	8	2.90	1.76	0.62	2.00	0.62	2.00	6.70
7	8	3.78	2.23	0.79	3.15	2.30	2.00	7.70
6 Day								
0	8	2.00	0.00	0.00	2.00	0.00	2.00	2.00
1	8	6.98	3.58	1.26	8.24	5.55	2.00	10.78
3	8	5.63	3.13	1.11	6.85	5.97	2.00	8.85
7	8	6.30	1.95	0.69	6.95	1.40	2.00	7.70

10 Day								
0	8	2.00	0.00	0.00	2.00	0.00	2.00	2.00
1	8	4.57	1.96	0.69	4.42	3.22	2.00	6.70
3	8	5.80	2.45	0.87	6.65	2.63	2.00	7.78
7	8	6.25	2.03	0.72	6.92	1.59	2.00	8.00

Of interest, the saline control group remained at baseline concentrations $\log_{10}$ CFU/g concentrations of *klebsiella* from day 0 to day 7. For the mice treated with one day of antibiotics, the mice had an average concentration of 2 $\log_{10}$ CFU/g while on antibiotics and days 1 and 3 after stopping antibiotics. By day 7, average *klebsiella* concentration increased slightly ($\mu = 2.12$, $\sigma = 0.12$), with one mice having a concentration of 3 $\log_{10}$ CFU/g. Overall concentrations remained at baseline and similar to the saline control group. Mice treated with 3 days of antibiotics had slightly more variability in their *klebsiella* stool concentrations. While on antibiotics, these mice had baseline concentrations of klebsiella ($\mu = 2.0$, $\sigma = 0.0$), however, once the mice were taken off of antibiotics, the concentration of antibiotics began increasing from day 1 ($\mu = 2.56$, $\sigma = 1.08$), to day 3 ($\mu = 2.56$, $\sigma = 1.76$), and peaking at day 7 ($\mu = 3.78$, $\sigma = 2.23$). A similar but greater trend was noticed from mice treated with a 6 day course of antibiotics. From baseline levels, the mice had a largest increase by day 1 ($\mu = 6.98$, $\sigma = 3.58$) of all groups. The concentrations decreased by day 3 ($\mu = 5.63$, $\sigma = 3.13$), and a slight increase by day 7 ($\mu = 6.30$, $\sigma = 1.95$). The 10 day antibiotic treatment duration mice also had a large increase *klebsiella* concentrations after ceasing antibiotics. Stool concentrations increased to 4.57 $\log_{10}$ CFU ($\sigma = 1.96$) shortly after stopping one day. Pathogens concentrations steadily increased by day 3 ($\mu = 5.80$, $\sigma = 2.45$) and peaked at day 7 ($\mu = 6.25$, $\sigma = 2.03$). Overall these trends suggest that mice that are on longer courses of antibiotics are more prone to colonization with *klebsiella*, with antibiotics treatment durations of 3,6, and 10 days leaving the mice most vulnerable to infection.

$\mathbf{Log_{10}}$ VRE Recovered by Antibiotic Group and Day

day	n	Mean	SD	SEM	Median	IQR	Min	Max
Saline								
0	6	2.00	0.00	0.00	2.00	0.00	2.00	2.00
1	6	2.00	0.00	0.00	2.00	0.00	2.00	2.00
3	6	2.00	0.00	0.00	2.00	0.00	2.00	2.00
7	6	2.00	0.00	0.00	2.00	0.00	2.00	2.00
1 Day								
0	8	2.00	0.00	0.00	2.00	0.00	2.00	2.00
1	8	2.00	0.00	0.00	2.00	0.00	2.00	2.00
3	8	2.00	0.00	0.00	2.00	0.00	2.00	2.00
7	8	2.00	0.00	0.00	2.00	0.00	2.00	2.00
3 Day								
0	8	2.00	0.00	0.00	2.00	0.00	2.00	2.00
1	8	2.93	2.02	0.72	2.00	0.44	2.00	7.70
3	8	5.23	2.44	0.86	6.13	3.97	2.00	7.90
7	8	3.15	2.13	0.75	2.00	1.15	2.00	6.60
6 Day								
0	8	2.00	0.00	0.00	2.00	0.00	2.00	2.00
1	8	6.11	3.33	1.18	7.24	6.27	2.00	9.30
3	8	6.10	3.50	1.24	7.42	7.12	2.00	9.48
7	8	5.01	2.73	0.97	5.65	4.97	2.00	8.85
10 Day								
0	8	2.00	0.00	0.00	2.00	0.00	2.00	2.00

1	8	4.50	2.36	0.84	4.10	3.03	2.00	7.90
3	8	5.41	2.84	1.00	7.04	5.55	2.00	7.95
7	8	4.73	2.39	0.84	5.59	4.60	2.00	7.48

A similar trend can be seen when the mice were co-challenged with VRE. The saline control and mice treated with one day of antibiotics had no increases in VRE concentrations in stool when challenged ($\mu = 2.0$, $\sigma = 0.0$). The mice treated with 3 days of antibiotics, saw a slight increase in VRE after 1 day ($\mu = 2.93$, $\sigma = 2.02$) with the highest concentrations happening by day 3 ($\mu = 5.23$, $\sigma = 2.44$), and a decrease by day 7 ($\mu = 3.15$, $\sigma = 2.13$). Mice with a antibiotic treatment duration of 6 days had a large increase after 1 day ($\mu = 6.11$, $\sigma = 3.33$) and steadily remained around this concentration by day 3 ($\mu = 6.10$, $\sigma = 3.50$) and day 7 ($\mu = 5.01$, $\sigma = 2.73$). Interestingly, the 10 day antibiotic treatment mice had a similar trend but to a lesser magnitude. By day 1 stool concentrations increased to an average of 4.50 $\log_{10}$ CFU ($\sigma = 2.36$) and peaked at day 3 ($\mu = 5.41$, $\sigma = 2.84$) with a slight decrease by day 7 ($\mu = 4.73$, $\sigma = 2.39$). As with *klebsiella* the mice showed a similar trend when challenged with VRE after being treated with antibiotics. The saline group and mice treated with one day of antibiotics had no infection when challenged with VRE, while mice treated with 3,6, and 10 day of antibiotics, all had a increase of VRE in their stool concentrations that remained elevated up to 7 days after being challenged.

1.4 EDA

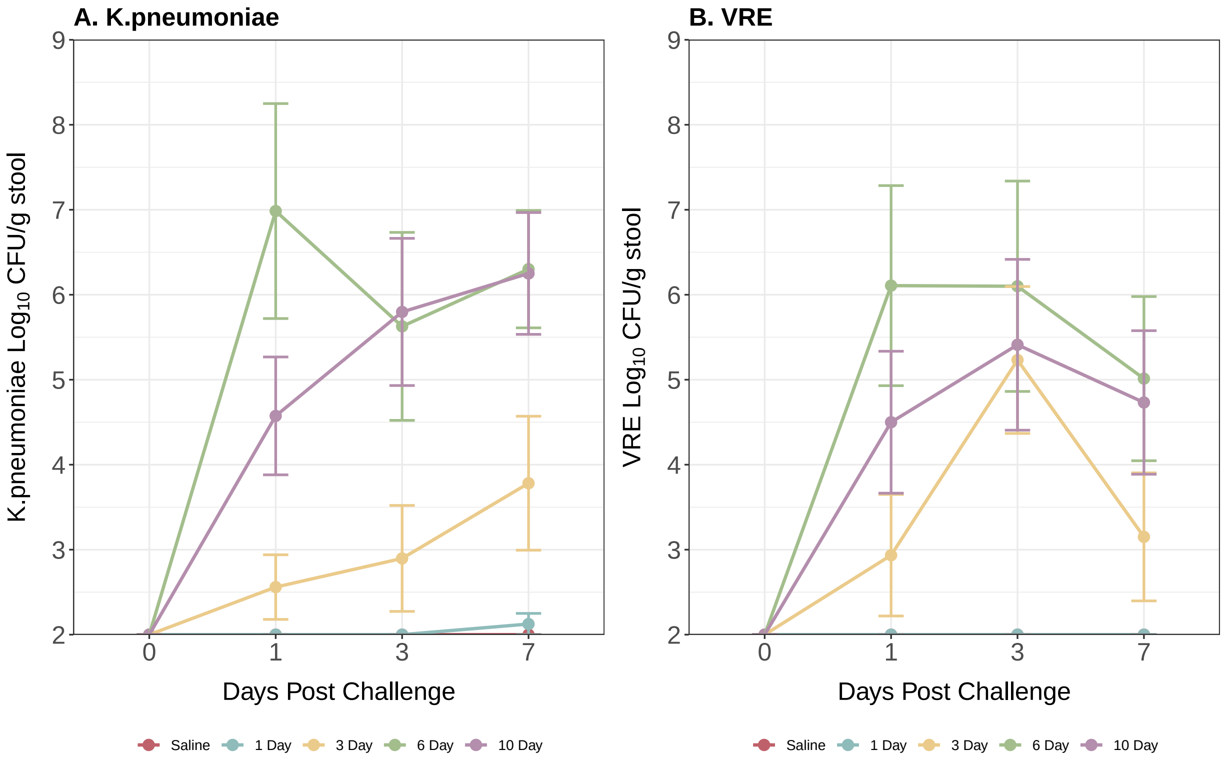

The figure shows the changes in colonization in mouse stool following varying durations of antibiotic treatment. Mice receiving longeer courses of antibiotics exhibted higher levels of *K.pneumoniae* and VRE compared to those with shorter durations of antibiotics (1 day). The 6 and 10 day antibiotic groups demostrated the highest increase in both *K.pneumoniae* and VRE by day 1 and maintained a higher burden of each organism by day 7.

1.5 Repeated Measures ANOVA (K.pneumoniae)

A repeated measures ANOVA is an extension of the classic ANOVA in which each subject provides measurements at multiple levels of a within-subject factor. In this analysis, we assess how $\log_{10}$ CFU stool concentrations varies with a between-subjects factor (`group`: Saline, Day 1, Day 3, Day 6, Day 10) and a within-subjects factor (`day`: 1, 3, 7, 14, 22) in mice challenged with *Klebsiella pneumoniae* after treatment with varying lengths of antibiotics.

The repeated measures ANOVA model partitions variance into:

- **Between-subjects effects** - Overall differences among antibiotic groups
- **Within-subject effects** - Changes in *Klebsiella pneumoniae* colonization levels over the `days`
- **Interaction** - Whether the day-to-day trajectory of colonizations differs by `day` and `group`
- **Residual** - The unexplained within-mouse variability

1.5.1 Model Diagnostics

The repeated measures ANOVA has the following assumptions:

- **Independence of subjects** - Each mouse's response is independent of every other's
- **Normality of residuals** - The within-subject residuals (deviations from each mouse's group by day mean are approximately normally distributed)
- **Sphericity (Homogeneity of covariances)** - The variances of the pairwise differences between levels of the within subject factor (`day`) are equal
- **Homoscedasticity of between-subjects factor** - The variance of observations across the levels of the between-subjects factor (`group`) is approximately equal
- **Balanced Design** - The within-subject levels are categorical and each subject has the same measurements at every level

1.5.1.1 Independence of Subjects

The subjects (`mice`) are independent of one another and had no effect on the other mice. All measurements came from the same mice at multiple time points

1.5.1.2 Normality of residuals

Normality of residuals in a repeated measure ANOVA is important because the model assumes the data is distributed around a central value which is important for valid F-tests. Deviation from normality may lead to misleading results such as inflated p-values.

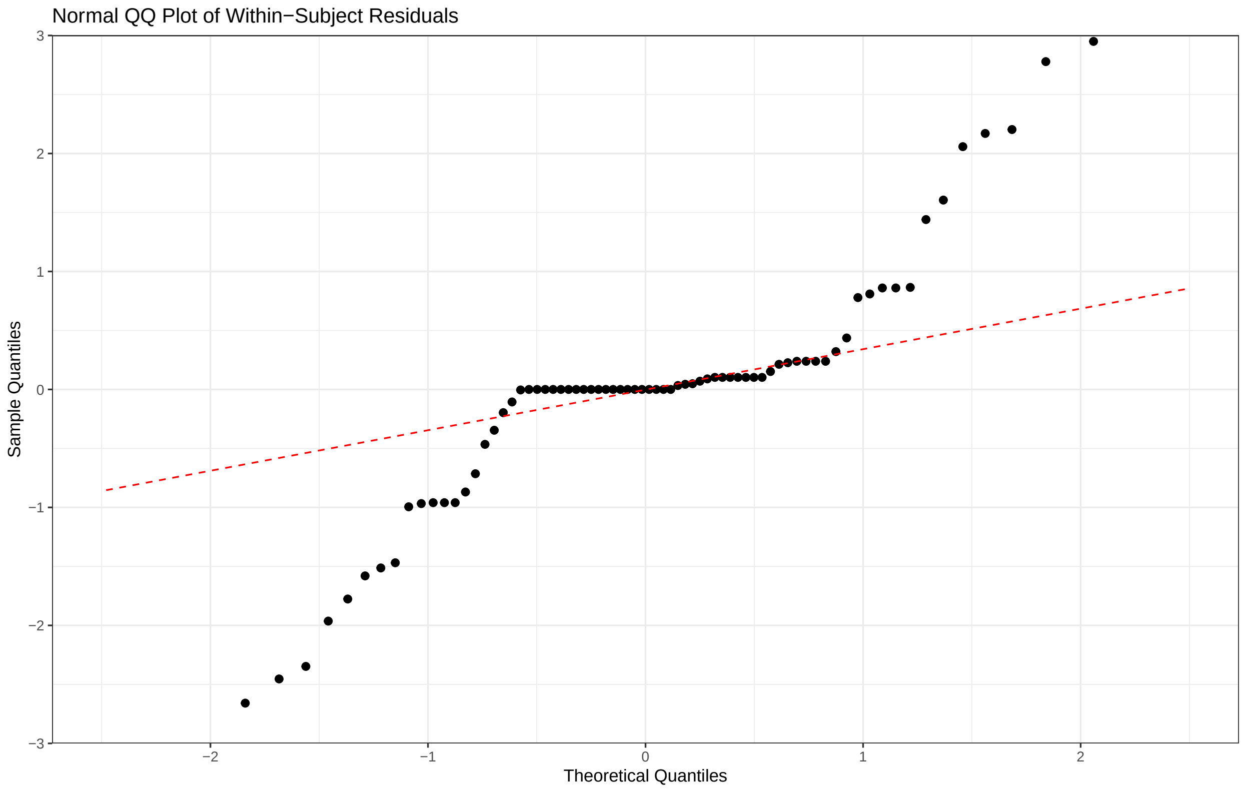

The QQ plot shows that there is fairly large deviations from normality in it's tails, while the middle values somewhat hug the red dashed values. Overall it seems that the normality assumption is violated for the repeated-measures ANOVA.

1.5.1.3 Sphericity

The sphericity assumption of the repeated measures ANOVA, is the assumption that the variances of the differences between all possible pairs of levels of the repeated measure factor (`day`) are equal. Violations of this assumption can lead to results being inflated and the higher probability of a type 1 error (False positive). Or in other words, it may lead to the repeated measures ANOVA may come to the false conclusion that there is a significant effect when there isn't one. A common test for sphericity is Mauchly's test. A significant p-value from a Mauchly's test suggests that sphericity has been violated:

Mauchly's Test for Sphericity

	W	p-value	p<.05
day	0.860	0.090	
group:day	0.860	0.090	

The table displays the results of the Mauchly's test. It corresponds to testing two effects of `day` (The main within-subjects effect) and `group:day` (The group x day interaction).

The columns are as follows:

- **W** - Mauchly's W statistic (range 0-1; smaller values indicate greater departure from spherecitiy)
- **p** - The significance test for equality of variances of all pairwise differences
- **p<.05** - An asterisk (*) if p < .05, indicating a significant violation

For both the **day** main effect and the **group** $\times$ **day** interaction, Mauchly's $W = 0.860$ with $p = 0.090$. Because $p > .05$ in each case, we fail reject the null hypothesis and proceed with our standard analysis

without needing to apply corrections for violations of sphericity.

1.5.1.4 Balanced Data The data contains no missing values for any of the trials, however there is an imbalance in the in the number of subjects for each of the antibiotic groups. Repeated-measures ANOVA typically require the same number of subjects when doing the comparisons, but the ANOVA is robust to moderate departures in it's assumptions.

1.5.2 Results

Repeated-Measures ANOVA Results (K.pneumoniae)

	DFn	DFd	SSn	SSd	F	p-value	p<.05	ges	
(Intercept)	1	33	1618.758015	222.2311	240.38	0.000	*	0.82305937	*
group	4	33	367.365485	222.2311	13.64	0.000	*	0.51353589	*
day	2	66	5.039592	125.7682	1.32	0.273		0.01427489	
group:day	8	66	20.199097	125.7682	1.32	0.247		0.05485928	

Mauchly's Test for Sphericity

	W	p-value	p<.05
day	0.860	0.090	
group:day	0.860	0.090	

The repeated measures ANOVA shows several significant effects.

The Mauchly's test revealed that there were no violations of sphericity for both the `day` and `group:day` effects had ($W = 0.860$; $p = 0.09$). There was a strong between-subject effect of antibiotic group ($F(4{,}33) = 13.64$, $p < 0.001$, generalized $\eta = 0.51$), indicating that pathogen concentrations levels within stool differed across at least one of the different antibiotic treatment durations.

Dunnett Post-hoc Comparisons

Each antibiotic group vs. saline for K.pneumoniae*

	Estimate	SE	DF	Lower 95% CI	Upper 95% CI	t-ratio	p-value
**1 Day Post-Treatment**							
1 Day - Saline	0.00	1.01	70	−2.54	2.54	0.00	1.000
3 Day - Saline	0.56	1.01	70	−1.98	3.10	0.55	0.913
6 Day - Saline	4.98	1.01	70	2.44	7.53	4.92	0.000
10 Day - Saline	2.57	1.01	70	0.03	5.12	2.54	0.046
**3 Day Post-Treatment**							
1 Day - Saline	0.00	1.01	70	−2.54	2.54	0.00	1.000
3 Day - Saline	0.90	1.01	70	−1.65	3.44	0.89	0.754
6 Day - Saline	3.63	1.01	70	1.08	6.17	3.58	0.002
10 Day - Saline	3.80	1.01	70	1.25	6.34	3.75	0.001
**7 Day Post-Treatment**							
1 Day - Saline	0.13	1.01	70	−2.42	2.67	0.12	0.998
3 Day - Saline	1.78	1.01	70	−0.76	4.33	1.76	0.247
6 Day - Saline	4.30	1.01	70	1.76	6.84	4.25	0.000

10 Day - Saline	4.25	1.01	70	1.71	6.79	4.20	0.000

Overall Group Comparisons

Comparisons of overall average K.pneumoniae CFU differences for antibiotics groups

	Estimate	SE	DF	Lower 95% CI	Upper 95% CI	t-ratio	p-value
Saline - 1 Day	−0.04	0.81	33	−2.48	2.39	−0.05	0.959
Saline - 3 Day	−1.08	0.81	33	−3.51	1.35	−1.33	0.701
Saline - 6 Day	−4.30	0.81	33	−6.74	−1.87	−5.32	0.000
Saline - 10 Day	−3.54	0.81	33	−5.97	−1.11	−4.38	0.001
1 Day - 3 Day	−1.04	0.75	33	−3.29	1.22	−1.39	0.701
1 Day - 6 Day	−4.26	0.75	33	−6.52	−2.01	−5.69	0.000
1 Day - 10 Day	−3.50	0.75	33	−5.75	−1.25	−4.67	0.000
3 Day - 6 Day	−3.22	0.75	33	−5.48	−0.97	−4.30	0.001
3 Day - 10 Day	−2.46	0.75	33	−4.71	−0.21	−3.29	0.012
6 Day - 10 Day	0.76	0.75	33	−1.49	3.02	1.02	0.701

1.6 Repeated Measures ANOVA (VRE)

1.6.1 Model Diagnostics

The repeated measures ANOVA has the following assumptions:

- **Independence of subjects** - Each mouse's response is independent of every other's
- **Normality of residuals** - The within-subject residuals (deviations from each mouse's group by day mean are approximately normally distributed)
- **Sphericity (Homogeneity of covariances)** - The variances of the pairwise differences between levels of the within subject factor (`day`) are equal
- **Homoscedasticity of between-subjects factor** - The variance of observations across the levels of the between-subjects factor (`group`) is approximately equal
- **Balanced Design** - The within-subject levels are categorical and each subject has the same measurements at every level

1.6.1.1 Independence of Subjects

The subjects (`mice`) are independent of one another and had no effect on the other mice. All measurements came from the same mice at multiple time points

1.6.1.2 Normality of residuals

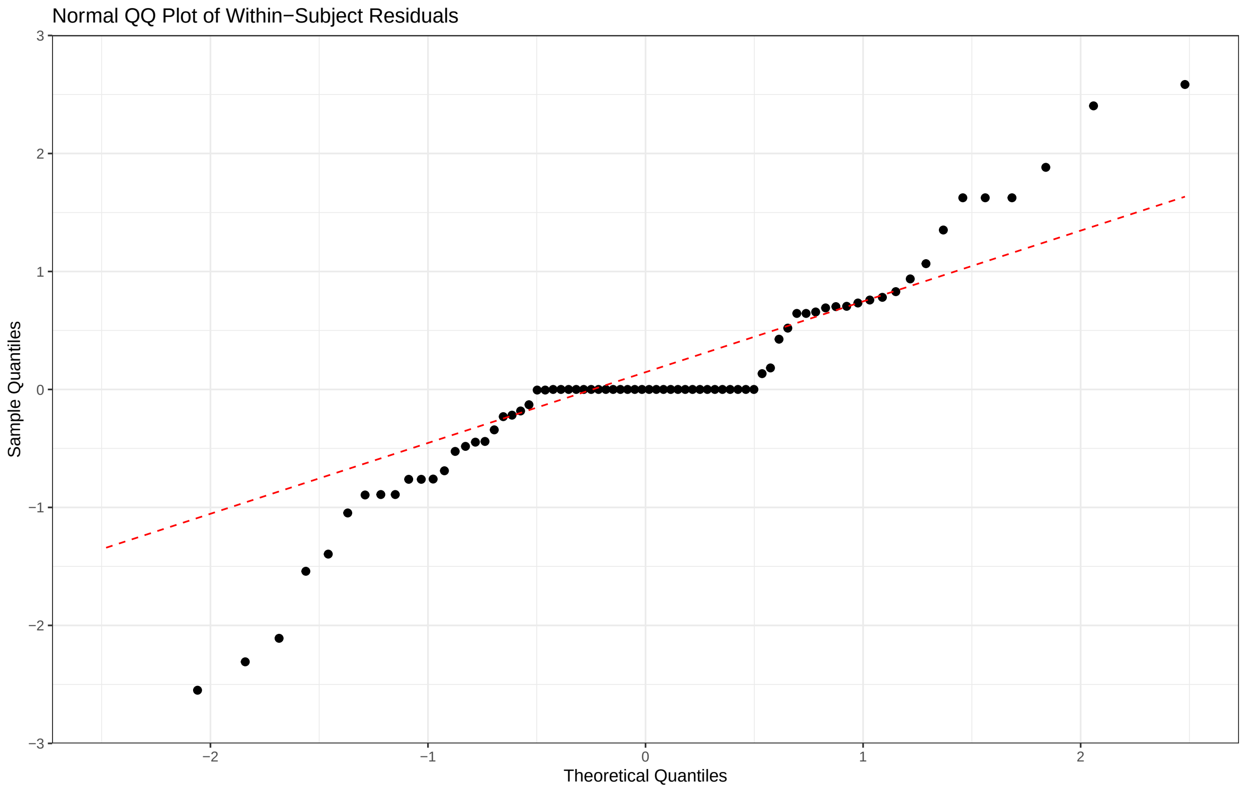

1.6.1.3 Sphericity

Mauchly's Test for Sphericity

	W	p-value	p<.05
day	0.919	0.260	
group:day	0.919	0.260	

The table displays the results of the Mauchly's test. It corresponds to testing two effects of `day` (The main within-subjects effect) and `group:day` (The group x day interaction).

The columns are as follows:

- **W** - Mauchly's W statistic (range 0-1; smaller values indicate greater departure from spherecitiy)
- **p** - The significance test for equality of variances of all pairwise differences
- **p<.05** - An asterisk (*) if p $<$ .05, indicating a significant violation

For both the **day** main effect and the **group** $\times$ **day** interaction, Mauchly's $W = 0.919$ with $p = 0.260$. Because $p > .05$ in each case, we fail reject the null hypothesis and proceed with our standard analysis without needing to apply corrections for violations of sphericity.

1.6.1.4 Balanced Data The data contains no missing values for any of the trials, however there is an imbalance in the in the number of subjects for each of the antibiotic groups. Repeated-measures ANOVA typically require the same number of subjects when doing the comparisons, but the ANOVA is robust to moderate departures in it's assumptions.

1.6.2 Results

Repeated-Measures ANOVA Results

	DFn	DFd	SSn	SSd	F	p-value	p<.05	ges	
(Intercept)	1	33	1522.42276	380.18913	132.14	0.000	*	0.77047470	*
group	4	33	254.33755	380.18913	5.52	0.002	*	0.35930028	*
day	2	66	12.73696	73.34238	5.73	0.005	*	0.02731678	*
group:day	8	66	21.39016	73.34238	2.41	0.024	*	0.04503934	*

Mauchly's Test for Sphericity

	W	p-value	p<.05
day	0.919	0.260	
group:day	0.919	0.260	

Dunnett Post-hoc Comparisons

Each antibiotic group vs. saline for VRE

	Estimate	SE	DF	Lower 95% CI	Upper 95% CI	t-ratio	p-value
1 Day Post-Treatment							
1 Day - Saline	0.00	1.16	46	−2.94	2.94	0.00	1.000
3 Day - Saline	0.93	1.16	46	−2.00	3.87	0.81	0.798
6 Day - Saline	4.11	1.16	46	1.17	7.05	3.55	0.003
10 Day - Saline	2.50	1.16	46	−0.44	5.44	2.16	0.117
3 Day Post-Treatment							
1 Day - Saline	0.00	1.16	46	−2.94	2.94	0.00	1.000
3 Day - Saline	3.23	1.16	46	0.29	6.17	2.80	0.027
6 Day - Saline	4.10	1.16	46	1.16	7.04	3.55	0.003
10 Day - Saline	3.41	1.16	46	0.47	6.35	2.95	0.018
7 Day Post-Treatment							
1 Day - Saline	0.00	1.16	46	−2.94	2.94	0.00	1.000
3 Day - Saline	1.15	1.16	46	−1.79	4.09	1.00	0.690
6 Day - Saline	3.01	1.16	46	0.07	5.95	2.61	0.043
10 Day - Saline	2.73	1.16	46	−0.21	5.67	2.36	0.076

Overall Group Comparisons

Comparisons of overall average VRE CFU differences for antibiotics groups

	Estimate	SE	DF	Lower 95% CI	Upper 95% CI	t-ratio	p-value
Saline - 1 Day	0.00	1.06	33	−3.18	3.18	0.00	1.000
Saline - 3 Day	−1.77	1.06	33	−4.96	1.41	−1.67	0.414
Saline - 6 Day	−3.74	1.06	33	−6.92	−0.56	−3.53	0.011
Saline - 10 Day	−2.88	1.06	33	−6.06	0.30	−2.72	0.072
1 Day - 3 Day	−1.77	0.98	33	−4.72	1.18	−1.81	0.398
1 Day - 6 Day	−3.74	0.98	33	−6.69	−0.79	−3.82	0.006

1 Day - 10 Day	−2.88	0.98	33	−5.83	0.07	−2.94	0.048
3 Day - 6 Day	−1.97	0.98	33	−4.92	0.98	−2.01	0.317
3 Day - 10 Day	−1.11	0.98	33	−4.06	1.84	−1.13	0.798
6 Day - 10 Day	0.86	0.98	33	−2.09	3.81	0.88	0.798

1.7 Linear Mixed Model (VRE)

1.7.1 Model Diagnostics

The linear mixed model has the following assumptions:

- **Linearity** – The expected CFU change is a linear function of the fixed effects (`group`, `day`, and `group` x `day`). Nonlinear function will result in biased estimates
- **Normality of Level-1 Residuals** – After accounting for the fixed effects and the random intercept, the remaining (within-in mouse) errors should be normal. This ensures proper *p*-values and CIs
- **Homoscedasticty within Groups** – Within each antibiotic `group`, the residual variance should be constant. (This is addressed using the constant variance function within the model which assigns group-specific variances)
- **Independence of Residuals** – For the random intercepts in the model, the residuals should be uncorrelated both within and between mice (The within mouse variance is captured with the random slope)
- **Normality of Random Effects** – The mouse-specific intercepts are assumed to follow a normal distribution
- **Correct Random-Effects Structure** – The within-mouse correlation is captured by a single intercept (instead of random slopes). If mice differ in how quickly they clear *C.auris* omitting a random slope could bias the fixed-effects SEs
- **No Perfect Multicollinearity** – The predictors must not be highly correlated with one another. Collinearity can inflate SEs and make hypothesis tests unreliable
- **Properly Nested Data** – Each observation must belong to one mice, and the clustering factor (`mice`) must be uniquely identify independent sampling units

1.7.1.1 Linearity

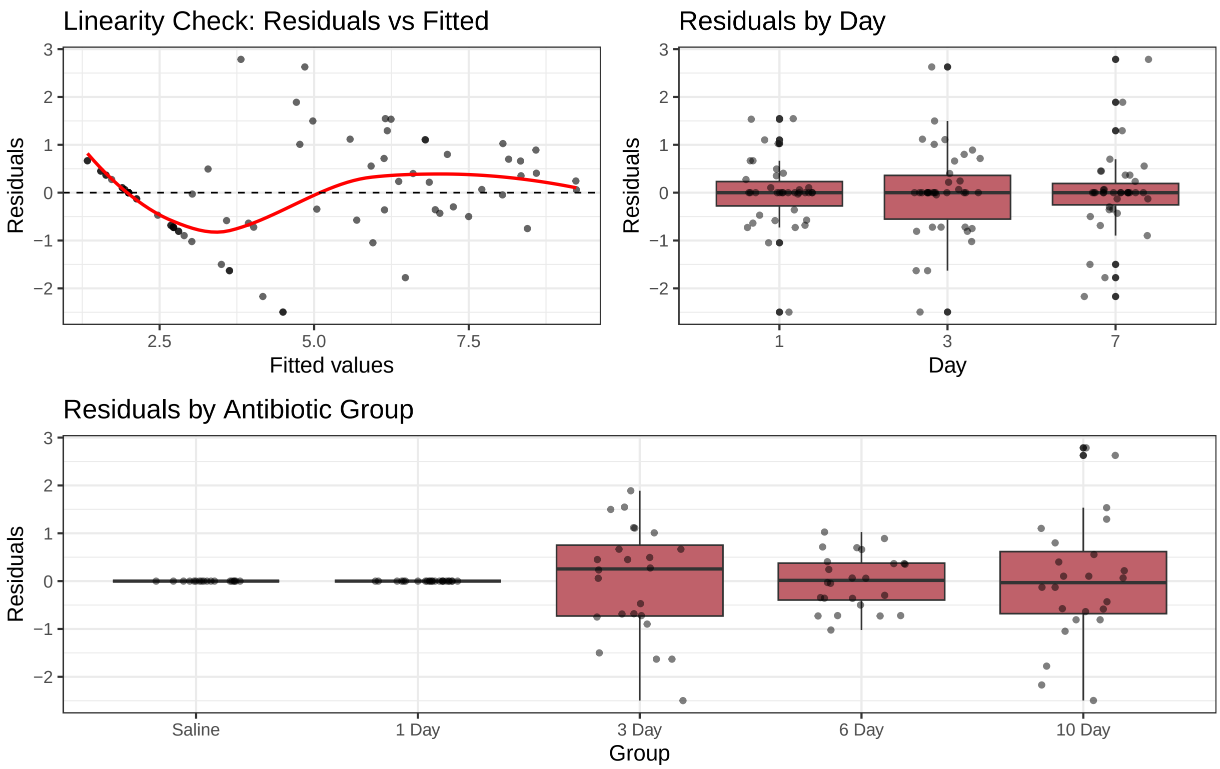

1.7.1.2 Normality of Level-1 Residuals

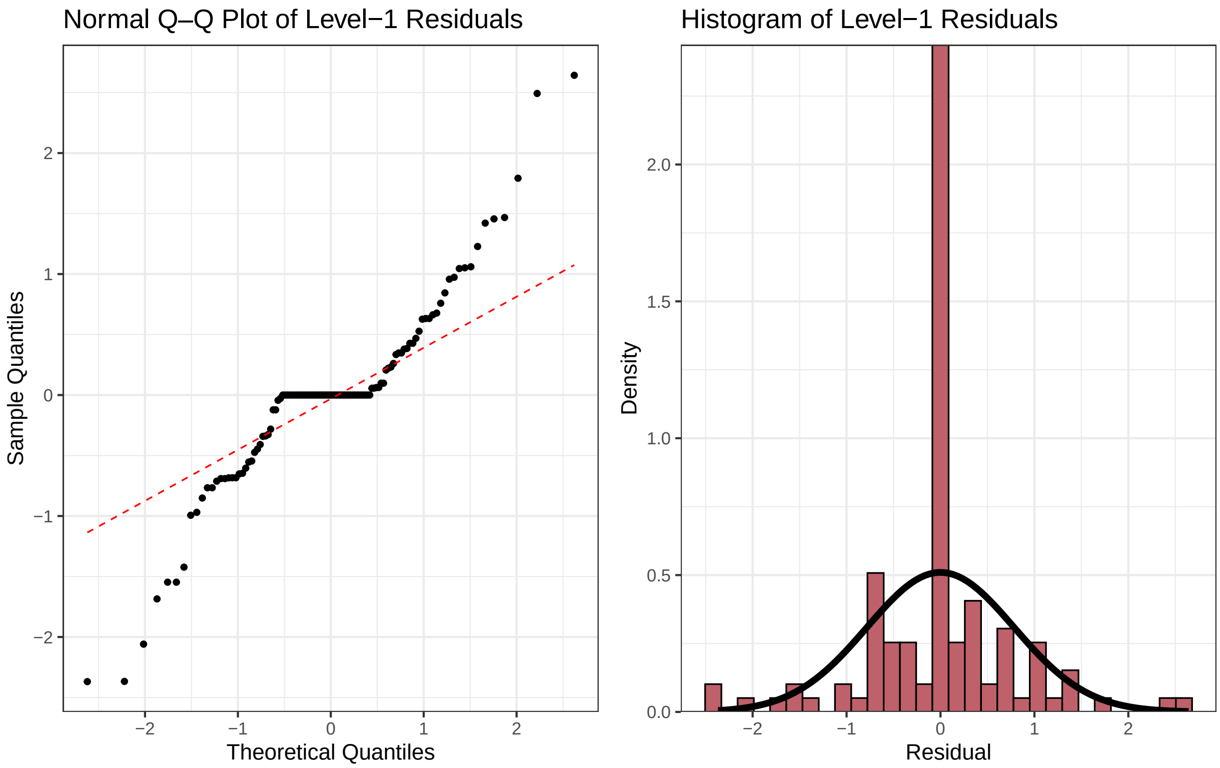

```
##
##  Shapiro-Wilk normality test
##
## data:  diag_df$resid
## W = 0.90565, p-value = 6.763e-07
```

1.7.1.3 Homoscedasticity within Groups

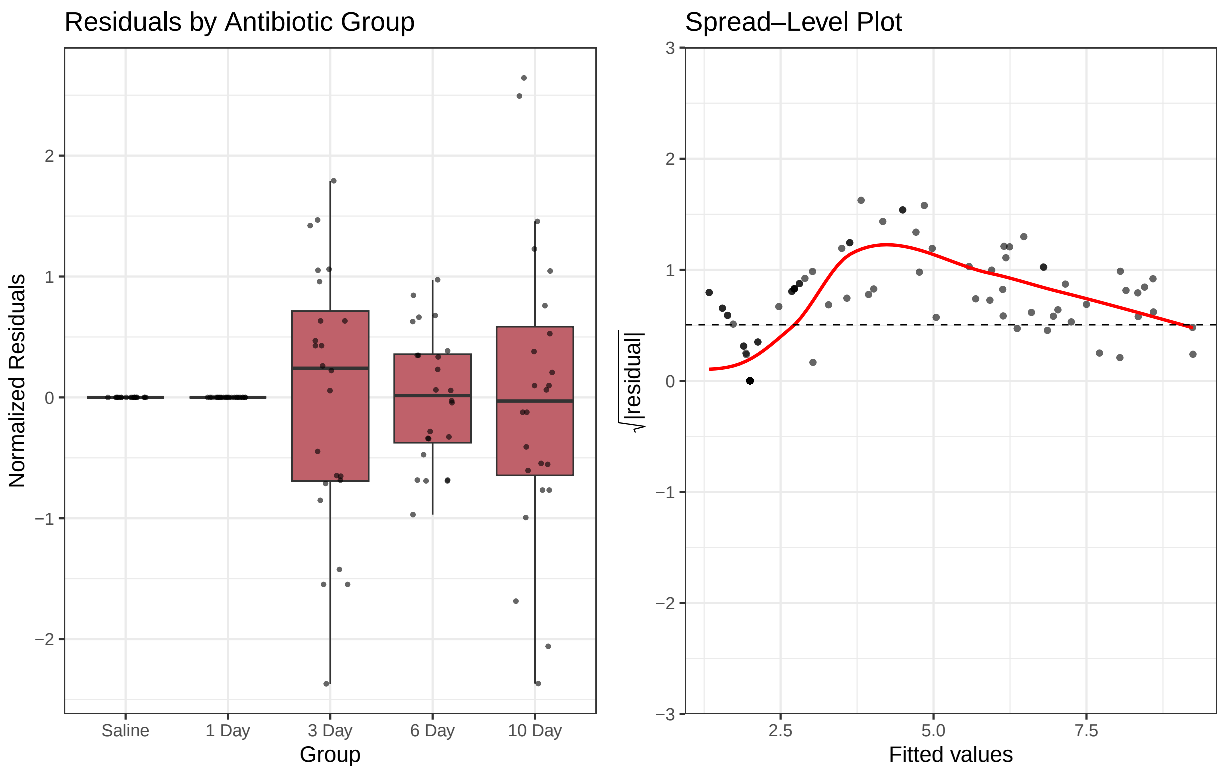


```
## Levene's Test for Homogeneity of Variance (center = median)
##        Df F value    Pr(>F)
## group   4  18.602 1.115e-11 ***
##       109
## ---
## Signif. codes:  0 '***' 0.001 '**' 0.01 '*' 0.05 '.' 0.1 ' ' 1
```

1.7.1.4 Independence of Residuals An ACF plot is used to represent the autocorrelation of time series data, to show how strongly data correlates with past values at different intervals (lags)

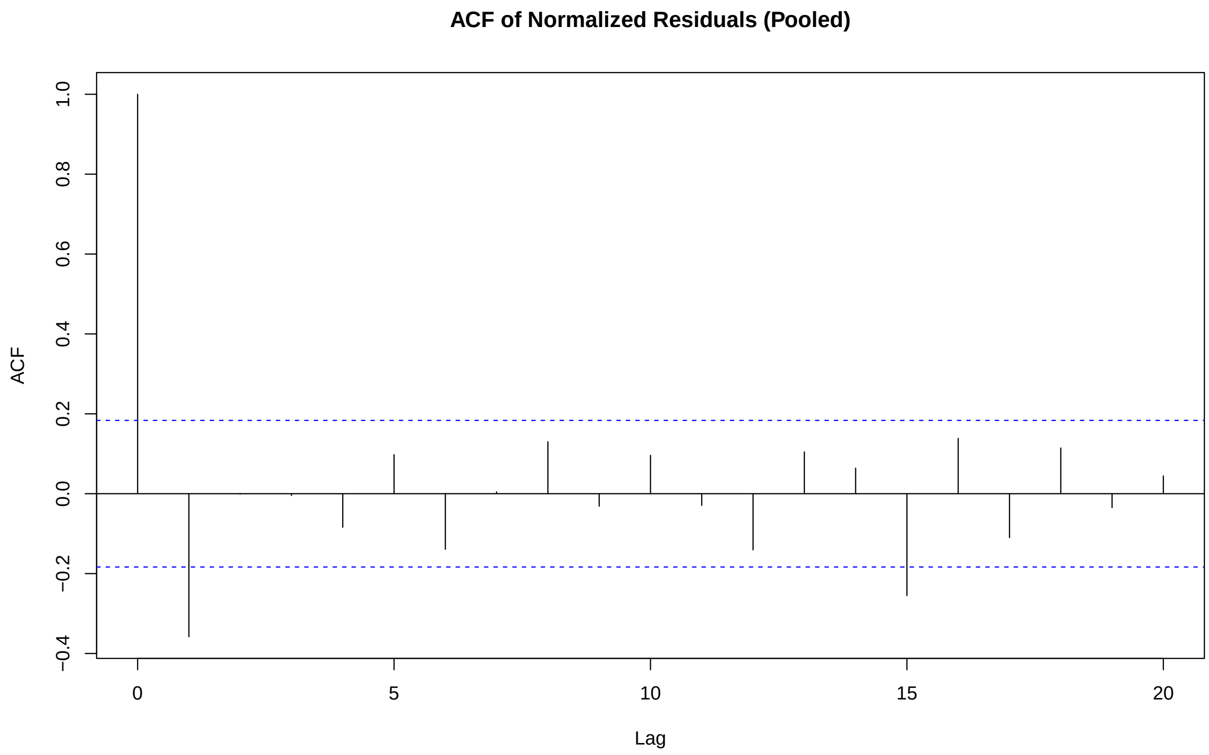
ACF of Normalized Residuals (Pooled)
ACF
Lag
1.0
0.8
0.6
0.4
0.2
0.0
−0.2
−0.4
0
5
10
15
20

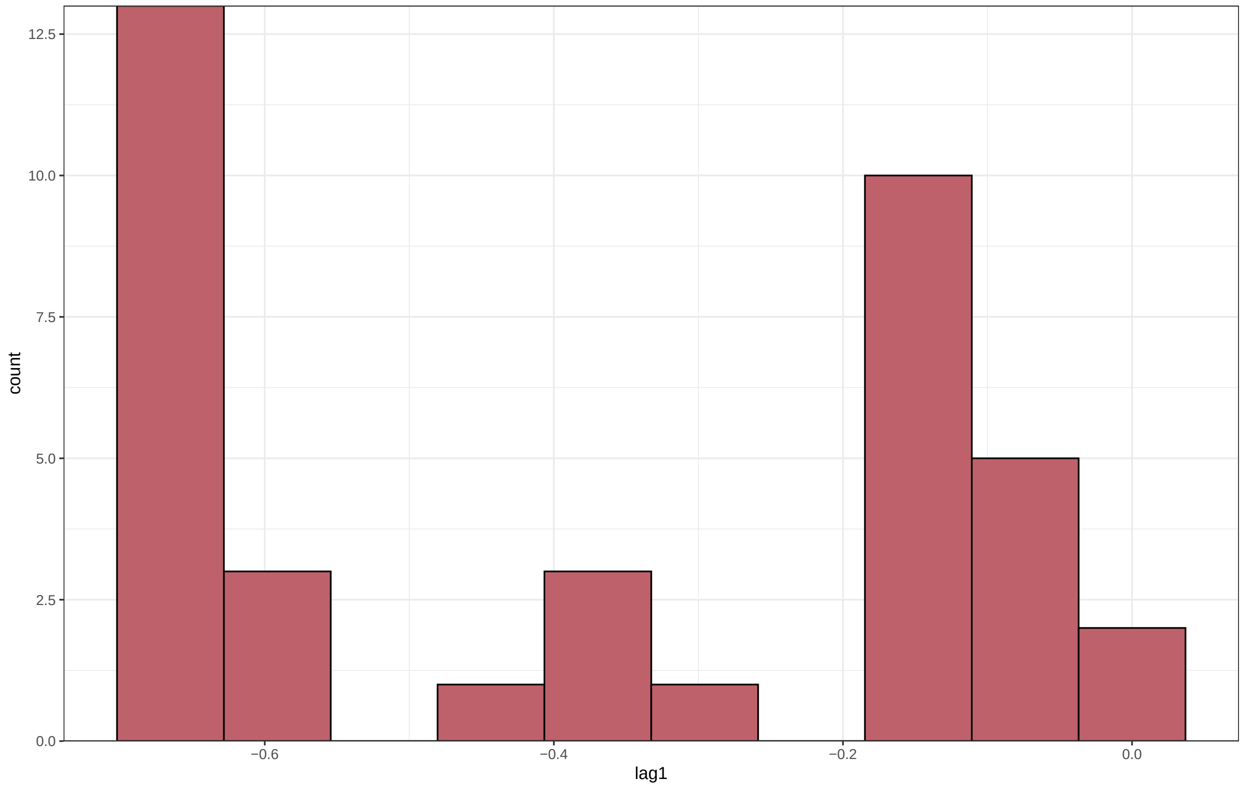
count
lag1
12.5
10.0
7.5
5.0
2.5
0.0
−0.6
−0.4
−0.2
0.0

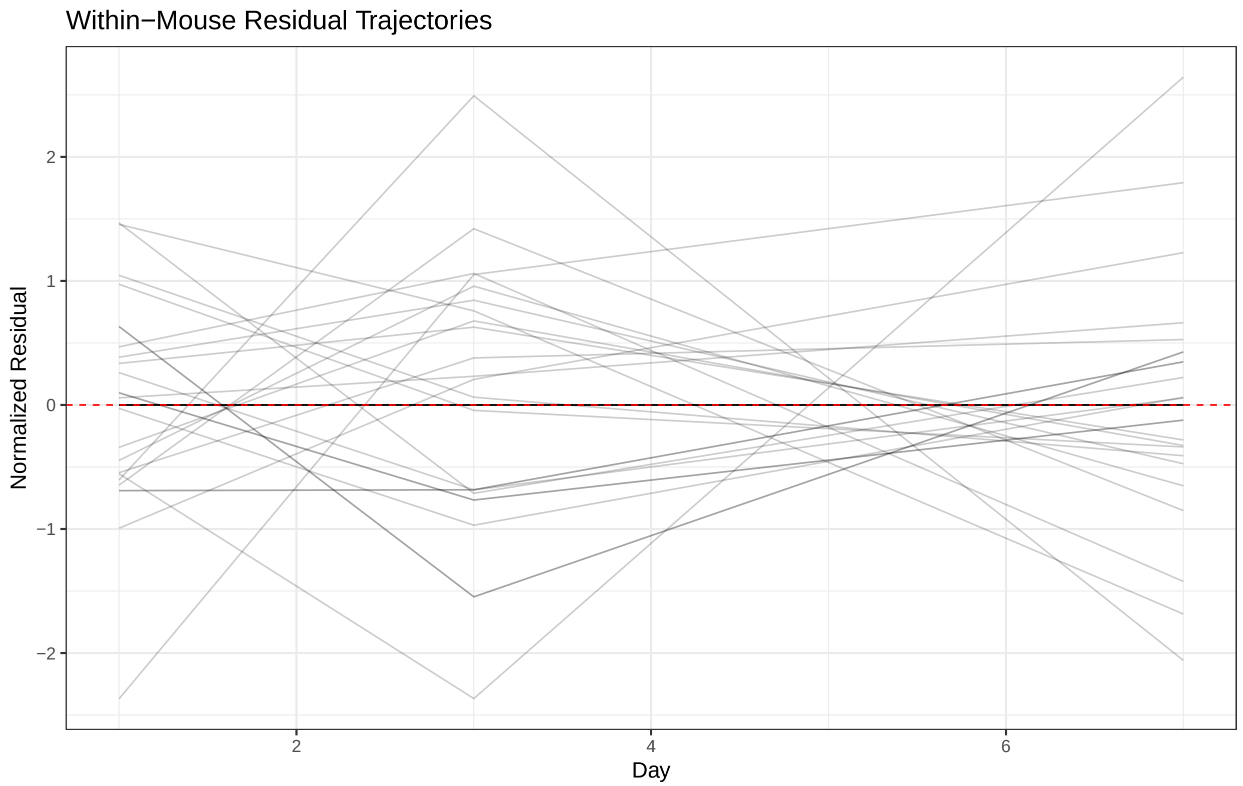

1.7.1.5 Normality of Random Effects

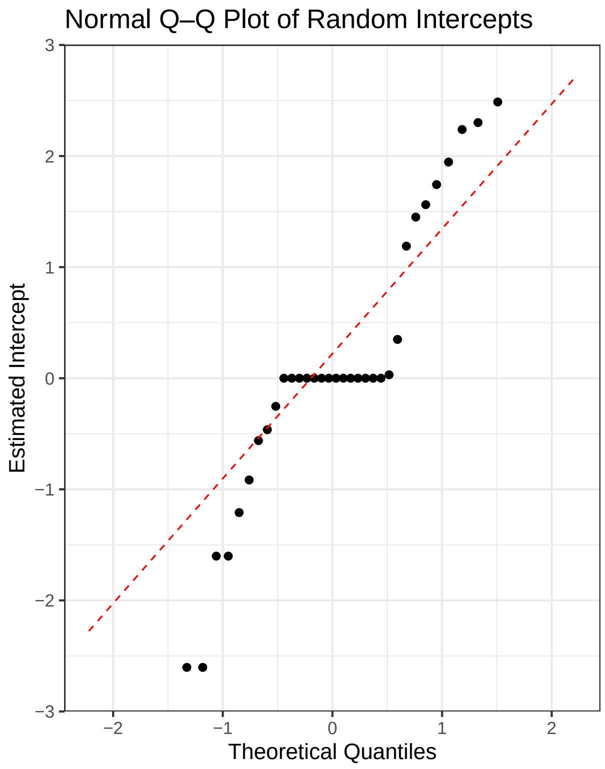

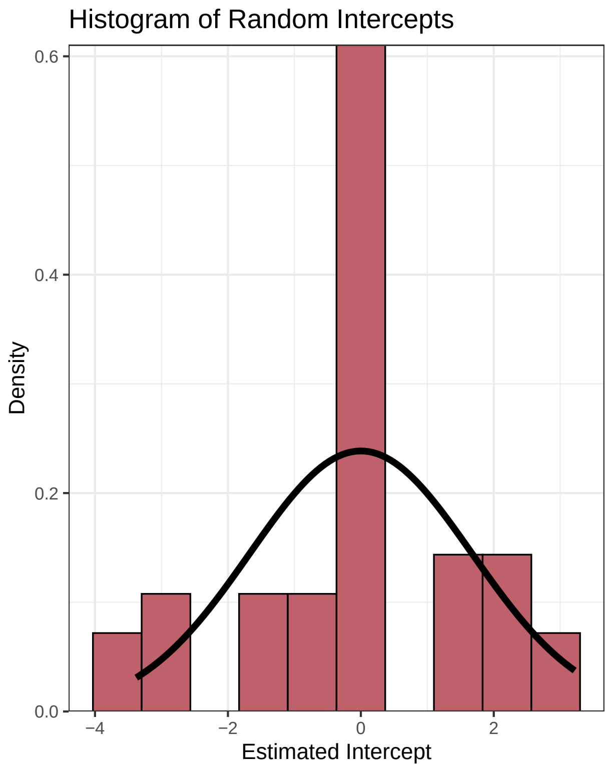

```
##
##  Shapiro-Wilk normality test
##
## data:  rand_eff$intercept
## W = 0.93082, p-value = 0.0213
```

1.7.1.6 Correct Random-Effects Structure

```
## mice = pdLogChol(1)
##             Variance StdDev
## (Intercept) 3.469878 1.862761
## Residual    1.111248 1.054158
```

```
## [1] 0.7574291
```

An ICC value of 0.75 indicates that roughly 75% of the total variability in $\log_{10}$ CFU is attributable to differences between mice, while the remaining the remaining variance arises within mice (over time and group effects). This large ICC justifies including a mouse-level random intercept, since mice do meaningfully differ in their baseline colonization.

1.7.1.7 No Perfect Multicollinearity

```
##               GVIF Df GVIF^(1/(2*Df))
## group     1.000000  4        1.000000
## day       1.026844  2        1.006645
## group:day 1.026844  8        1.001657
```

The VIF terms are very large (where values greater than 10 are considered severe issues) but this is an unadjusted inflation factor on multi-df terms. The GVIF^2 terms provide a reasonable estimate indicating that we have no major issues with collinearity.

1.7.1.8 Properly Nested Data All `mice` ID appear in exactly one group (no mouse switches between antibiotic conditions) and each mice has the same number of observations (one per level of `day`), so the repeated-measures structure is balanced and nested. This indicates our data is properly nested for a random-intercept model.

1.7.2 Results

Model Fit Statistics

Statistic	Value
AIC	453.54
BIC	497.66
logLik	−209.77

Random-Effects Variance Components

	Variance	StdDev
(Intercept)	3.470	1.863
Residual	1.111	1.054

Fixed Effects Estimates – Linear Mixed Model (VRE)

Modeling CFU by group × day × organism

Term	Estimate	SE	Lower 95% CI	Upper 95% CI	z	p-value
(Intercept)	3.68	0.32	3.04	4.32	11.50	0.000
1 Day Abx	−1.68	0.70	−3.10	−0.26	−2.41	0.022
3 Day Abx	−1.68	0.62	−2.95	−0.41	−2.69	0.011
6 Day Abx	0.09	0.62	−1.18	1.37	0.15	0.882
10 Day Abx	2.06	0.62	0.79	3.33	3.30	0.002
3	−0.17	0.14	−0.45	0.11	−1.21	0.230
7	0.47	0.14	0.19	0.75	3.34	0.001
1 Day Abx × 3	0.17	0.31	−0.44	0.78	0.56	0.580
3 Day Abx × 3	0.17	0.27	−0.38	0.72	0.62	0.537
6 Day Abx × 3	−0.67	0.27	−1.22	−0.12	−2.43	0.018
10 Day Abx × 3	0.54	0.27	−0.01	1.09	1.96	0.054
1 Day Abx × 7	−0.47	0.31	−1.08	0.14	−1.53	0.130
3 Day Abx × 7	−0.47	0.27	−1.02	0.08	−1.71	0.092
6 Day Abx × 7	0.99	0.27	0.44	1.54	3.61	0.001
10 Day Abx × 7	−0.11	0.27	−0.66	0.44	−0.40	0.690

A linear mixed effects model model was used to examine how VRE colonization ($\log_{10}$ CFU/g) was influenced by antibiotic treatment duration ten days after treatment. The model found several significant effects for the various antibiotic groups, on day 7, and several significant interaction terms.

Dunnett Post-hoc Comparisons

Each antibiotic group vs. saline at each post-treatment day for VRE

	Estimate	SE	DF	Lower 95% CI	Upper 95% CI	t-ratio	p-value
**1 Day Post-Treatment**							
1 Day - Saline	0.00	1.16	46	−3.00	3.00	0.00	1.000
3 Day - Saline	0.93	1.16	46	−2.07	3.94	0.81	0.846
6 Day - Saline	4.11	1.16	46	1.10	7.11	3.55	0.004
10 Day - Saline	2.50	1.16	46	−0.50	5.50	2.16	0.107
**3 Day Post-Treatment**							
1 Day - Saline	0.00	1.16	46	−3.00	3.00	0.00	1.000
3 Day - Saline	3.23	1.16	46	0.23	6.24	2.80	0.015
6 Day - Saline	4.10	1.16	46	1.10	7.10	3.55	0.004
10 Day - Saline	3.41	1.16	46	0.41	6.42	2.95	0.015
**7 Day Post-Treatment**							
1 Day - Saline	0.00	1.16	46	−3.00	3.00	0.00	1.000
3 Day - Saline	1.15	1.16	46	−1.85	4.16	1.00	0.650
6 Day - Saline	3.01	1.16	46	0.01	6.02	2.61	0.049
10 Day - Saline	2.73	1.16	46	−0.27	5.74	2.36	0.067

To evaluate how antibiotic duration influences *vancomycin-resistant Enterococcus* (VRE) colonization over time, Dunnett-adjusted pairwise comparisons between each antibiotic group and the saline control at 1,3, and 7 days post challenge. One day after inoculation with VRE, there were no significant differences between from the saline group for the 1,3, and 10 day groups (all $p > 0.107$). The 6 day antibiotic group had a significantly higher colonization level with VRE ($\delta = 4.11$; 95% CI [1.10, 7.11]; $p = 0.004$). Three days after infection, colonization with VRE increased for several groups. The 3 ($\delta = 3.23$; 95% CI [0.23, 6.24]; $p =$

0.015) day and 10 day ($\delta = 3.41$; 95% CI [0.41, 6.42]; $p = 0.015$) antibiotic groups became significantly higher in colonization compared to the saline group. The 6 day antibiotic treatment group continued to remain significantly different ($p = 0.004$) while the 1 day antibiotic group continued to show no difference in VRE colonization ($p > 0.99$). Seven days after inoculation, there was no significant difference between the saline group and the 1-day antibiotic group ($p > 0.99$) and the 3-day antibiotic group ($p = 0.650$). The 10-day antibiotic group had a higher, but non-significant, burden of VRE compared to the saline group ($\delta = 2.73$; 95% CI [-0.27, 5.74]; $p = 0.067$). The 6-day antibiotic group remained significantly higher than the saline group ($\delta = 3.01$; 95% CI [0.01, 6.02]; $p = 0.049$).

These results indicate a duration-dependent effect of antibiotics on VRE susceptibility. By day 3 post-inoculation, mice pre-treated with antibiotics for longer than 3 days exhibited significantly higher colonization compared to saline controls. This reinforces the idea that extended antibiotics use disrupts the microbiota and thus colonization resistance and makes an individual more susceptible to VRE overgrowth, with measurable differences emerging by 72 hours post-infection.

Overall Group Comparisons (VRE)

Comparisons of overall differences for antibiotics group in colonization

	Estimate	SE	DF	Lower 95% CI	Upper 95% CI	t-ratio	p-value
Saline - 1 Day	0.00	1.06	33	−3.18	3.18	0.00	1.000
Saline - 3 Day	−1.77	1.06	33	−4.96	1.41	−1.67	0.414
Saline - 6 Day	−3.74	1.06	33	−6.92	−0.56	−3.53	0.011
Saline - 10 Day	−2.88	1.06	33	−6.07	0.30	−2.72	0.072
1 Day - 3 Day	−1.77	0.98	33	−4.72	1.18	−1.81	0.398
1 Day - 6 Day	−3.74	0.98	33	−6.69	−0.79	−3.82	0.006
1 Day - 10 Day	−2.88	0.98	33	−5.83	0.07	−2.94	0.048
3 Day - 6 Day	−1.97	0.98	33	−4.92	0.98	−2.01	0.317
3 Day - 10 Day	−1.11	0.98	33	−4.06	1.84	−1.13	0.798
6 Day - 10 Day	0.86	0.98	33	−2.09	3.81	0.88	0.798

To assess the impact of antibiotic treatment duration on overall *vancomycin resistant Enterocuccus* (VRE) colonization pairwise comparisons were performed across all antibiotic treatment duration groups independent of post-infection day. These contrasts reflect the cumulative differences in colonization levels of VRE throughout the experiment.

Compared to the saline group, all antibiotic groups except 1 day antibiotic group showed increased colonization, with varying statistical significance. The 6-day ($\delta = 3.74$; 95% CI [0.56, 6.92]; $p = 0.011$) and 10-day ($\delta = 2.88$; 95% CI [0.30, 6.06]; $p = 0.07$) groups had the largest differences from the saline group, but only the 6-day antibiotic group reached significance while the 10-day group trended to significance. The 3-day group showed a moderate increase in VRE colonization compared to the saline group ($\delta = 1.77$; 95% CI [-1.41, 4.96]; $p = 0.414$) through this difference was not statistically significant. Within antibiotic groups longer duration were generally associated with significantly higher VRE colonization. When comparing the 1-day antibiotic group against the 3-day antibiotic group there was no significant difference between the ($p = 0.398$). The 6 and 10 day antibiotic groups were significantly higher compared to the 1-day group ($p < 0.05$). There was no significant different pairwise comparisons between the 3,6, and 10 day antibiotic groups (all $p > 0.317$).

1.7.3 Conclusions

The model and pairwise contrasts found that longer durations of antibiotics were associated with higher colonization levels of VRE.

1.8 Linear Mixed Model (*K.pneumoniae*)

A linear mixed effects model is an extension of the ordinary regression model that incorporates fixed effects (i.e., population parameters such as antibiotic `group`, `day`, and their interaction) and random effects (Subject-

specific deviations that account for correlation among repeated measures, such as a random intercept for each mouse to account for baseline CFU levels or random slopes for antibiotics to account for different clearance rates). The model specified incorporated the fixed effects of `group` and `day` and their interaction in predicting Log_{10} CFU levels of *K.pneumoniae*. A random intercept was incorporated for each mice to allow them to have their own average baseline CFU levels on day 1. Furthermore, a variance identity structure was used to allow each antibiotic and organism group to have it's own residual variance. This allows each group to have it's own variance σ^2 rather than assuming each group has the same. This is due to the past plots and Levene's tests showing mice from different antibiotic differing in their variance.

1.8.1 Model Diagnostics

The linear mixed model has the following assumptions:

- **Linearity** – The expected CFU change is a linear function of the fixed effects (`group`, `day`, and `group` x `day`). Nonlinear function will result in biased estimates
- **Normality of Level-1 Residuals** – After accounting for the fixed effects and the random intercept, the remaining (within-in mouse) errors should be normal. This ensures proper *p*-values and CIs
- **Homoscedasticty within Groups** – Within each antibiotic `group`, the residual variance should be constant. (This is addressed using the constant variance function within the model which assigns group-specific variances)
- **Independence of Residuals** – For the random intercepts in the model, the residuals should be uncorrelated both within and between mice (The within mouse variance is captured with the random slope)
- **Normality of Random Effects** – The mouse-specific intercepts are assumed to follow a normal distribution
- **Correct Random-Effects Structure** – The within-mouse correlation is captured by a single intercept (instead of random slopes). If mice differ in how quickly they clear *C.auris* omitting a random slope could bias the fixed-effects SEs
- **No Perfect Multicollinearity** – The predictors must not be highly correlated with one another. Collinearity can inflate SEs and make hypothesis tests unreliable
- **Properly Nested Data** – Each observation must belong to one mice, and the clustering factor (`mice`) must be uniquely identify independent sampling units

1.8.1.1 Linearity

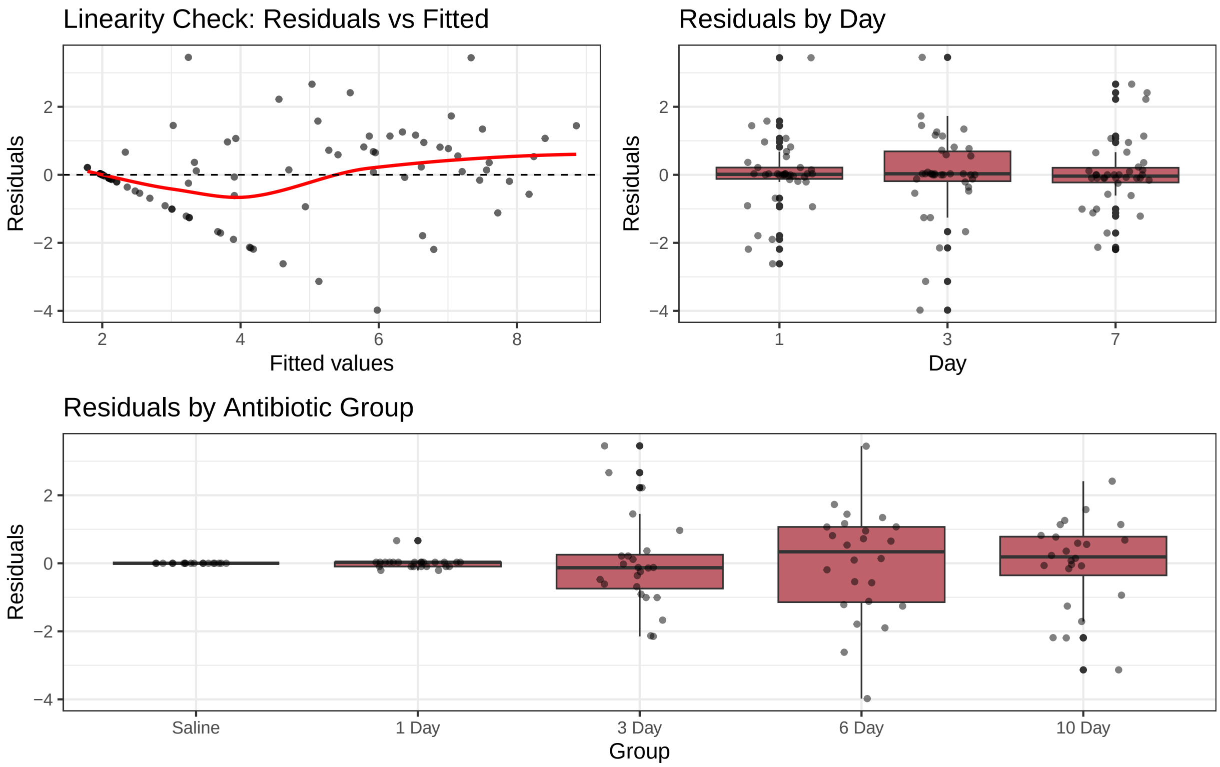

The diagnostics plot suggest that the assumption is mostly met. For the residuals vs. fitted plot, the red LOESS line is flat around zero for most of the fitted values, indicating that the model is capturing the mean structure well. The residual plot by `day` shows the majority of residuals hover around 0 with no clear trend of shifting of residual below or above the center. Variability by `day` appears to be relevantly consistent. The residual plot by `antibiotic` group shows the that majority of residuals are centered around zero. However there is show variability in the spread between the groups. Overall, there appears to be no glaring nonlinearity and no systematic bias in the residuals by `day` or `group`.

1.8.1.2 Normality of Level-1 Residuals

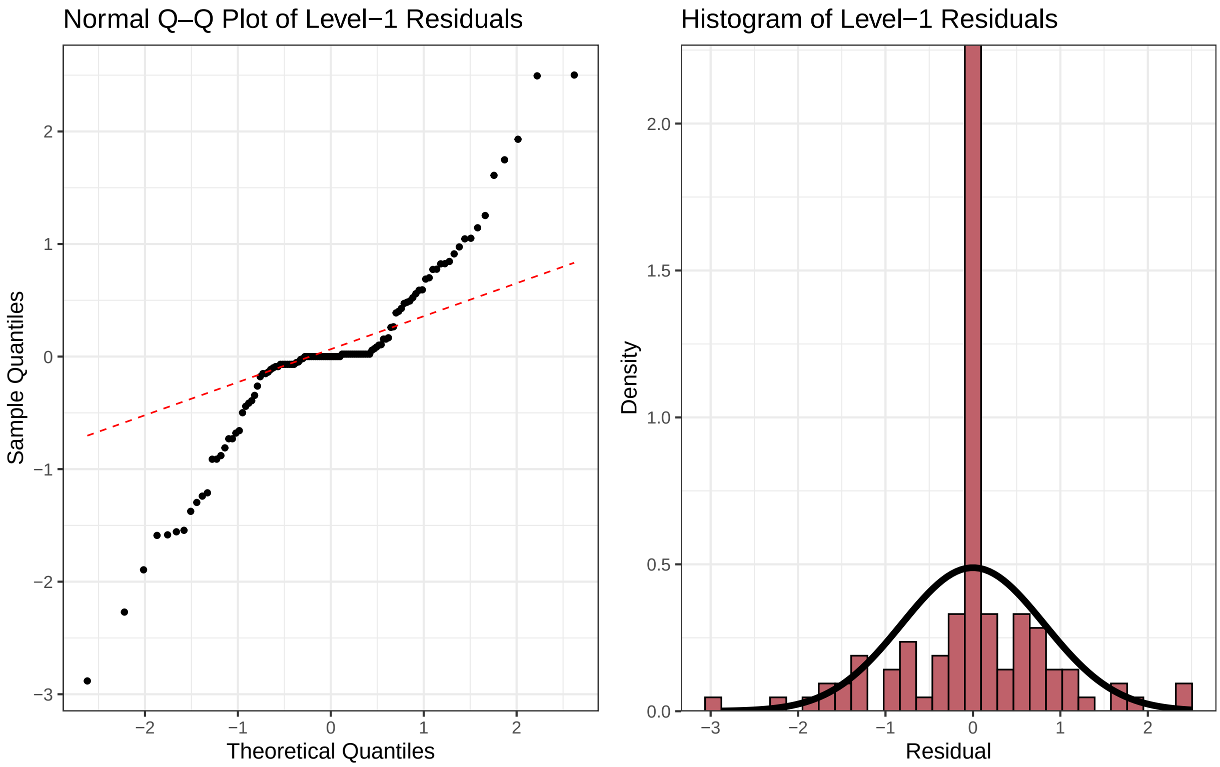


```
##
##  Shapiro-Wilk normality test
##
## data:  diag_df$resid
## W = 0.90817, p-value = 9.143e-07
```

The Shapiro test is highly significant ($p < 0.001$) indicating that the Level-1 residuals deviate from a normal distribution. However, small deviations at the extreme will trigger the Shapiro-Wilk significance with a large number of observations. The normal Q-Q plot and histogram confirm the deviation from normality for the residuals. Specifically, the Q-Q plot shows the middle residuals follow the normal line, but they are heavy deviations in the tails. Overall the assumption appears to be violated but due to lienar mixed models being able to tolerate moderate departures, we can proceed with the model. Of note, this issue can be handled by applying robust SEs to mitigate the damage of the extreme outliers.

1.8.1.3 Homoscedasticity within Groups

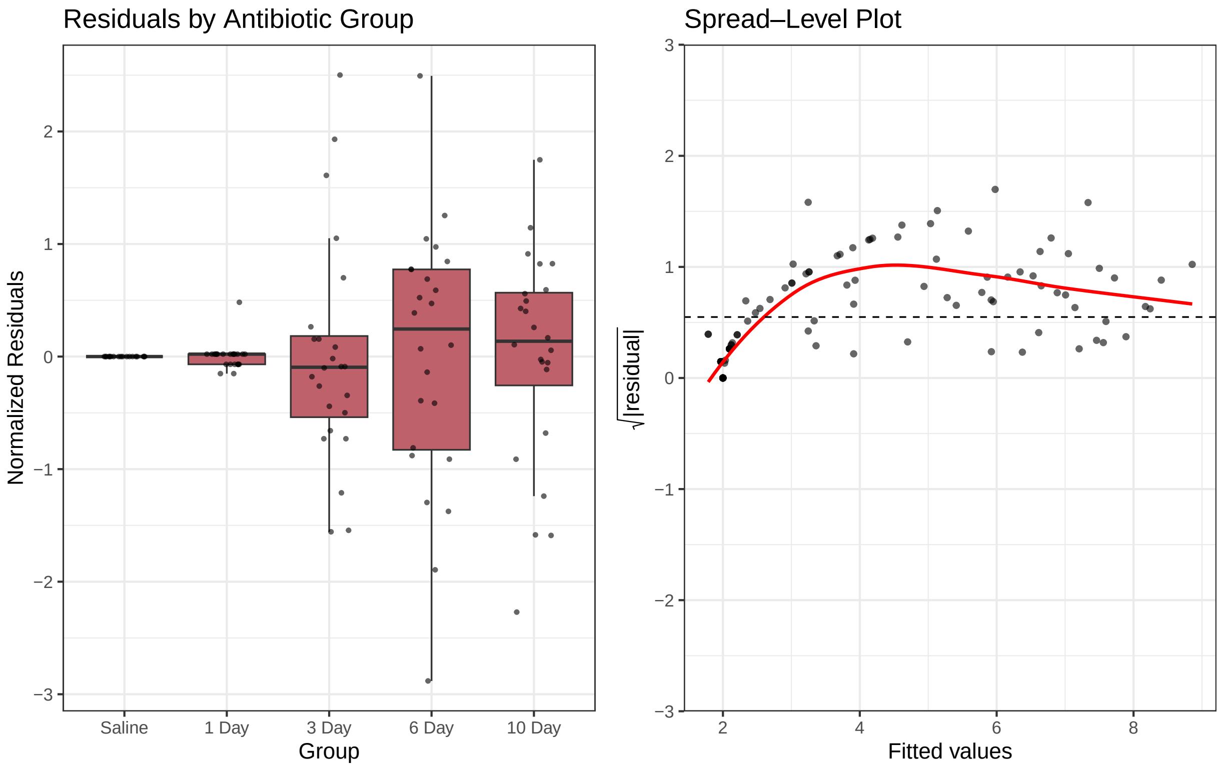


```
## Levene's Test for Homogeneity of Variance (center = median)
##        Df F value    Pr(>F)
## group   4  11.571 7.248e-08 ***
##       109
## ---
## Signif. codes:  0 '***' 0.001 '**' 0.01 '*' 0.05 '.' 0.1 ' ' 1
```

Levene's test provided a p-value < 0.001, so we fail to retain the null hypothesis indicating there are equal variances across the five antibiotic durations.

The residual plot by antibiotic shows that the antibiotics groups of 3,6, and 10 days have wide spread compared to the saline and 1 day group. The spread-plot shows the LOESS curve close to the horizontal line, indicating the residual variability does not trend systematically with the fitted CFU values. There is a slight curve indicating a bit or residual heteroskedasticity, but the curvature is fairly small and mostly driven by low-range values.

1.8.1.4 Independence of Residuals An ACF plot is used to represent the autocorrelation of time series data, to show how strongly data correlates with past values at different intervals (lags)

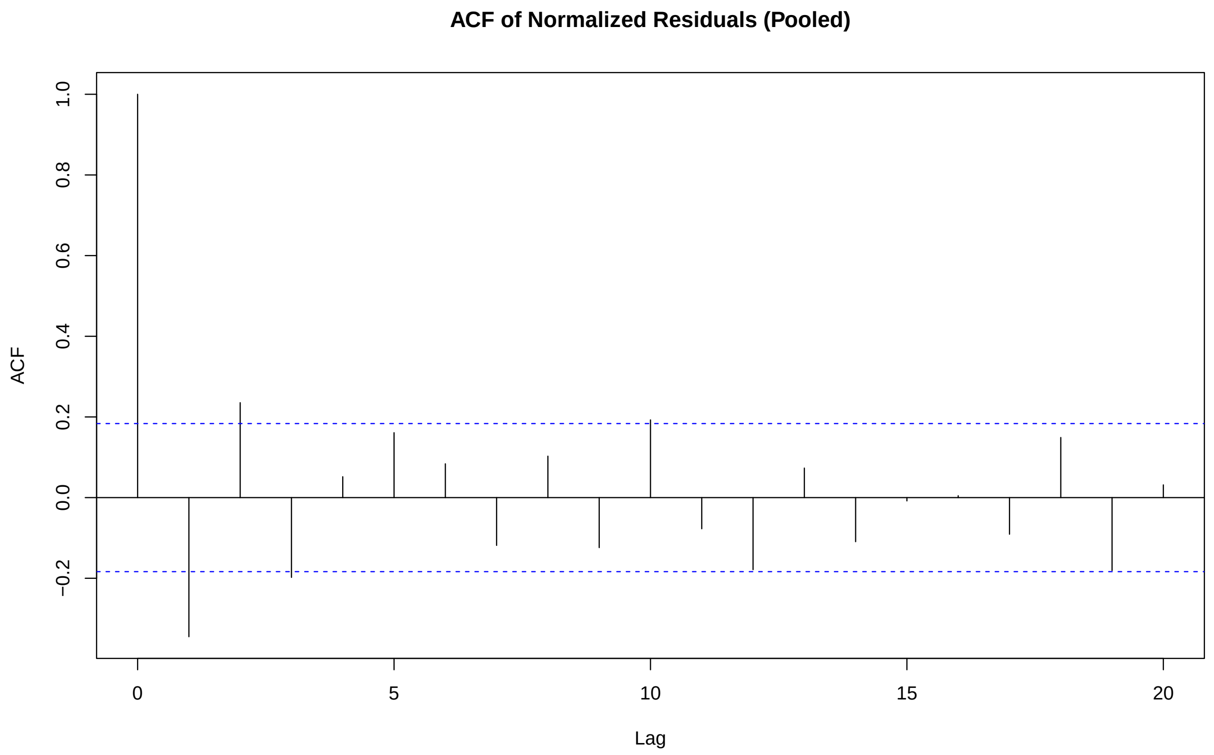

For the pooled ACF table, aside for lag-0, all autocorrelation bars lie well inside the dashed bounds, with no systematic spikes at lag 1 or beyond. This indicates that overall, the residuals are not serially correlated

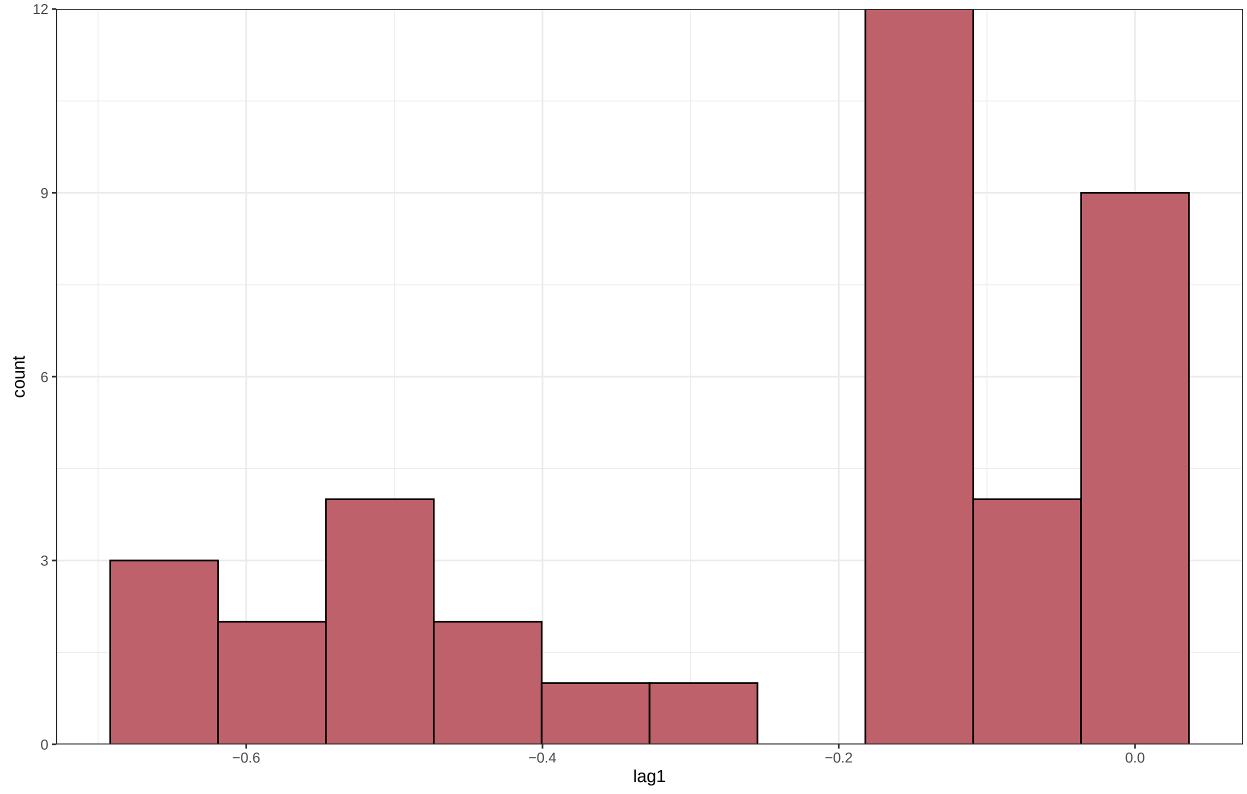

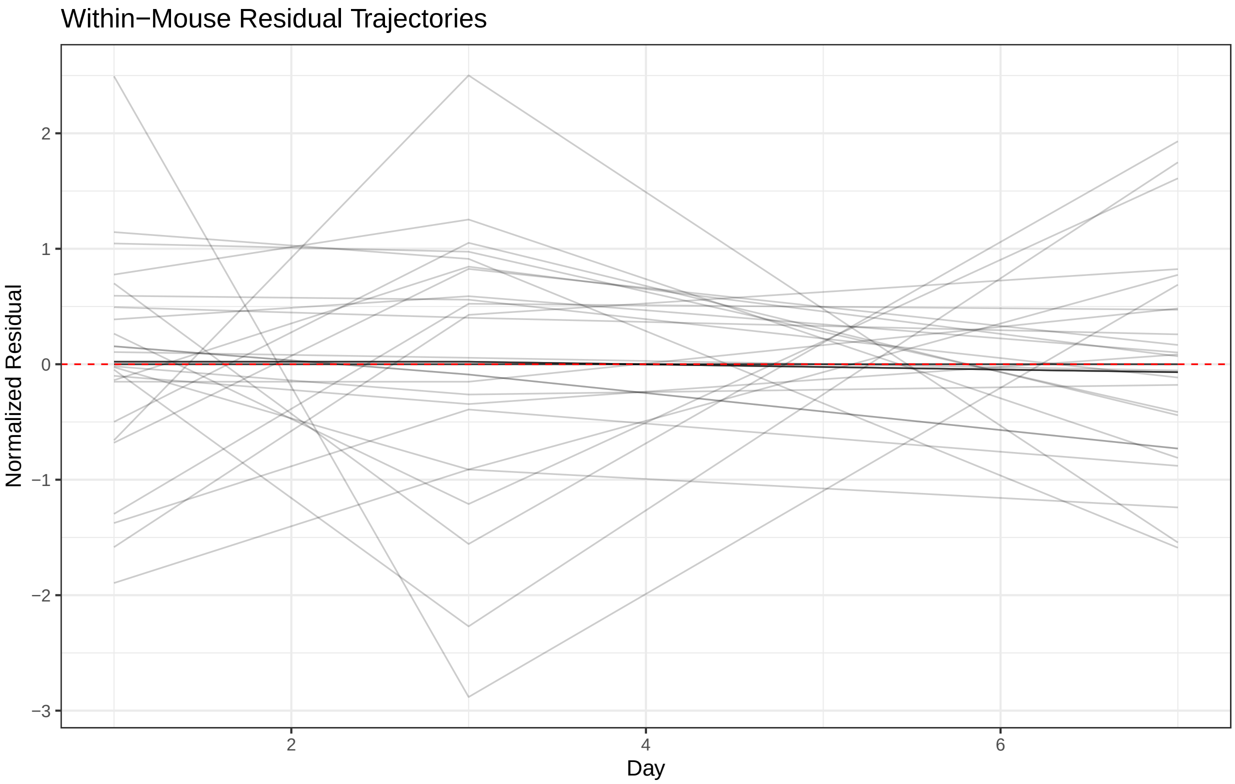

For the within-mice trajectories, the lines cross back and forth over zero without long runs above or below. If residuals were dependent, smooth drifts or runs within the individual mice would appear instead of random bouncing around. The histogram shows a normal distribution with clusters around zero.

1.8.1.5 Normality of Random Effects

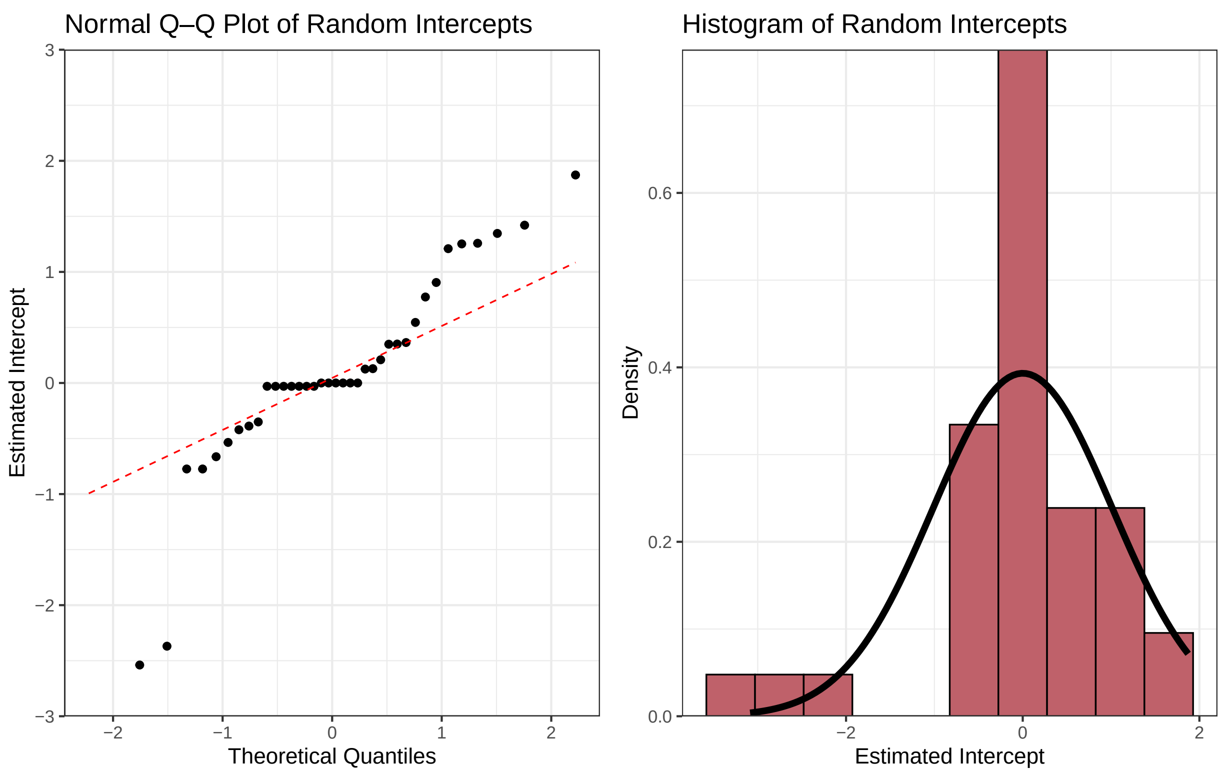


```
##
##  Shapiro-Wilk normality test
##
## data:  rand_eff$intercept
## W = 0.87359, p-value = 0.0004966
```

The Q-Q plot shows that most points fall along the reference line, with only slight deviations at the extreme tails. The histogram density plot shows a slightly distorted bell-shaped curve around zero. The Shapiro-Wilk returned a $W = 0.87$ and $p < 0.001$, so we fail to reject the normality of the random effects.

1.8.1.6 Correct Random-Effects Structure

```
## mice = pdLogChol(1)
##             Variance StdDev
## (Intercept) 1.609566 1.268687
## Residual    1.905579 1.380427
```

```
## [1] 0.4578948
```

1.8.1.7 No Perfect Multicollinearity

```
##                 GVIF Df GVIF^(1/(2*Df))
## group     1.000000  4        1.000000
## day       1.026844  2        1.006645
## group:day 1.026844  8        1.001657
```

The VIF terms are very large (where values greater than 10 are considered severe issues) but this is an unadjusted inflation factor on multi-df terms. The GVIF^2 terms provide a reasonable estimate indicating that we have no major issues with collinearity.

1.8.1.8 Properly Nested Data

All `mice` ID appear in exactly one group (no mouse switches between antibiotic conditions) and each mice has the same number of observations (one per level of `day`), so the repeated-measures structure is balanced and nested. This indicates our data is properly nested for a random-intercept model.

1.8.2 Results

Model Fit Statistics

Statistic	Value
AIC	471.41
BIC	515.53
logLik	−218.71

Random-Effects Variance Components

	Variance	StdDev
(Intercept)	1.610	1.269
Residual	1.906	1.380

Fixed Effects Estimates – Linear Mixed Model (K.pneumoniae)

Modeling CFU by group × day × organism

Term	Estimate	SE	Lower 95% CI	Upper 95% CI	z	p-value
(Intercept)	3.79	0.24	3.30	4.28	15.50	0.000
1 Day Abx	−1.79	0.53	−2.88	−0.71	−3.36	0.002
3 Day Abx	−1.75	0.48	−2.72	−0.78	−3.67	0.001
6 Day Abx	−0.71	0.48	−1.69	0.26	−1.49	0.145
10 Day Abx	2.51	0.48	1.54	3.48	5.26	0.000
3	−0.17	0.18	−0.54	0.20	−0.92	0.360
7	−0.13	0.18	−0.50	0.24	−0.70	0.486
1 Day Abx × 3	0.17	0.40	−0.63	0.97	0.42	0.674
3 Day Abx × 3	0.13	0.36	−0.59	0.85	0.36	0.723
6 Day Abx × 3	−0.35	0.36	−1.07	0.37	−0.97	0.333
10 Day Abx × 3	0.85	0.36	0.13	1.57	2.36	0.021
1 Day Abx × 7	0.13	0.40	−0.67	0.93	0.32	0.749
3 Day Abx × 7	0.09	0.36	−0.63	0.80	0.24	0.809
6 Day Abx × 7	−0.05	0.36	−0.77	0.66	−0.15	0.882
10 Day Abx × 7	−0.55	0.36	−1.27	0.17	−1.52	0.132

Dunnett Post-hoc Comparisons

Each antibiotic group vs. saline at each post-treatment day for Klebsiella

	Estimate	SE	DF	Lower 95% CI	Upper 95% CI	t-ratio	p-value
1 Day Post-Treatment							
1 Day - Saline	0.00	1.01	70	−2.60	2.60	0.00	1.000
3 Day - Saline	0.56	1.01	70	−2.04	3.16	0.55	1.000
6 Day - Saline	4.98	1.01	70	2.39	7.58	4.92	0.000

10 Day - Saline	2.57	1.01	70	−0.02	5.17	2.54	0.040
3 Day Post-Treatment							
1 Day - Saline	0.00	1.01	70	−2.60	2.60	0.00	1.000
3 Day - Saline	0.90	1.01	70	−1.70	3.49	0.89	0.757
6 Day - Saline	3.63	1.01	70	1.03	6.22	3.58	0.002
10 Day - Saline	3.80	1.01	70	1.20	6.39	3.75	0.001
7 Day Post-Treatment							
1 Day - Saline	0.13	1.01	70	−2.47	2.72	0.12	0.902
3 Day - Saline	1.78	1.01	70	−0.81	4.38	1.76	0.166
6 Day - Saline	4.30	1.01	70	1.70	6.90	4.25	0.000
10 Day - Saline	4.25	1.01	70	1.65	6.85	4.20	0.000

To evaluate the impact of antibiotic duration on *Klebsiella pneumonia* colonization, Dunnet-adjusted pairwise comparisons were performed between each antibiotic group and saline control at 1,3, and 7 days post-inoculation with *Klebsiella pneumonia*.

One day after infection with *K.pneumonia* the 3-day antibiotic group showed a positive but non-significant increase ($\delta = 0.56$; 95% CI [-2.04, 3.16]; $p > 0.99$), and the 1-day group showed no difference ($p > 0.99$), suggesting minimal impact of shorter antibiotic exposures on early colonization with *K.pneumonia*. Only the 6-day ($\delta = 4.98$; 95% CI [2.39, 7.58]; $p < 0.001$) and 10-day ($\delta = 2.57$; 95% CI [-0.02, 5.17]; $p = 0.04$) antibiotic groups showed significantly elevated CFu compared to the saline control, indicating increased susceptibility to *Klebsiella* colonization.

By day three, significant increases in *Klebsiella* burden were seen in the 6-day ($\delta = 3.63$; 95% CI [1.03, 6.22]; $p = 0.002$) and 10-day antibiotic ($\delta = 3.80$; 95% CI [1.20, 6.39]; $p = 0.001$) groups. The 3-day antibiotic group had increase in *Klebsiella* colonization but the difference was non-significant ($p = 0.757$). The one-day antibiotic group continued to remain statistically-indistinguishable different ($p > 0.99$).

Seven days after infection, all antibiotic groups saw an increase in their colonization burden. However, the day-1 antibiotic group continued to remain non-significantly different from the saline group ($p = 0.902$). The 3-day saline group peaked with their *Klebsiella* stool concentration with an average of 1.78 $\log_{10}$ CFU/g (SE = 1.01; 95% CI [-0.81, 4,38]) and trended toward significance ($p = 0.166$). The 6 and 10 day groups had an average *Klebsiella* stool concentration greater than 4 $\log_{10}$ CFU/g (both $p < 0.001$).

These results highlight a clear duration-dependent effect of antibiotic exposure on *Klebsiella pneumonia* colonization. Mice treated with one day of antibiotics did not have increased susceptibility to *K.pneumonia* colonization. However, durations longer than 3 days showed significantly compromised levels of host resistance to *K.pneumonia*, with the most pronounced effects following 6 and 10 days of antibiotics with significant differences appearing 1 day after colonization. Three days of treatment found non-significant differences from the saline group, but increasing *K.pneumonia* stool burden one week after infection.

Overall Group Comparisons for K.pneumoniae

Comparisons of overall differences for antibiotics group in colonization

	Estimate	SE	DF	Lower 95% CI	Upper 95% CI	t-ratio	p-value
Saline - 1 Day	−0.04	0.81	33	−2.48	2.39	−0.05	0.959
Saline - 3 Day	−1.08	0.81	33	−3.51	1.35	−1.33	0.701
Saline - 6 Day	−4.30	0.81	33	−6.74	−1.87	−5.32	0.000
Saline - 10 Day	−3.54	0.81	33	−5.97	−1.11	−4.38	0.001
1 Day - 3 Day	−1.04	0.75	33	−3.29	1.22	−1.39	0.701
1 Day - 6 Day	−4.26	0.75	33	−6.52	−2.01	−5.69	0.000
1 Day - 10 Day	−3.50	0.75	33	−5.75	−1.25	−4.67	0.000
3 Day - 6 Day	−3.22	0.75	33	−5.48	−0.97	−4.30	0.001

3 Day - 10 Day	-2.46	0.75	33	-4.71	-0.21	-3.29	0.012
6 Day - 10 Day	0.76	0.75	33	-1.49	3.02	1.02	0.701

Compared to the saline group, colonization was significantly elevated in the 6-day ($\delta = 4.30$; 95% CI [1.87, 6.74]; $p < 0.001$) and 10-day ($\delta = 3.54$; 95% CI [1.11, 5.97]; $p < 0.001$) antibiotic groups, while the 3-day group was non-significantly different from the saline group ($p = 0.701$). Shorter regimens, like the 1-day treatment group did not significantly alter colonization with *K.pneumonia*, suggesting the preservation of colonization resistance under brief antibiotic pressure. Direct comparisons between antibiotics groups further confirmed this pattern. Both 1-day vs. 6-day and 1-day vs 10-day groups ($p < 0.001$) were both highly significant, confirming that extended antibiotics use leads to higher colonization compared to brief exposure. There was no significant difference between the 1-day and 3 day antibiotic treatment groups, indicating a potential plateau in susceptibility beyond 3 days.

These results indicate a clear, duration-dependent increase in *K.pneumonia* colonization following different antibiotic treatment durations. Mice treated with longer than 3 days of antibiotics showed significantly higher levels of colonization with *K.pneumonia* compared to the control group, while durations shorter than or equal to 3 days were not significantly different from the saline controls. These results indicate that longer antibiotic treatments enhance the susceptibility to pathogenic like *K.pneumonia*.

1.8.3 Conclusions

K.pneumonia colonization was more pronounced in mice treated with longer durations of antibiotics, while shorter durations of antibiotics showed no significant difference from the saline group.
