# Supplementary material for: Longer durations of piperacillin/tazobactam treatment cause more prolonged alteration of colonization resistance in mice
Source: PLoS One. 2026 Jun 1;21(6):e0350031. doi: 10.1371/journal.pone.0350031 (PMC13225349; doi:10.1371/journal.pone.0350031)
Supplement: S3 Table — (PDF) [file pone.0350031.s003.pdf]

Klebsiella and VRE Infection Day 24

Samir Memic

2025-06-10

Contents

1 KP and VRE Colonization after 24 days **1**
- 1.1 Description . . . 1
- 1.2 Loading the Data . . . 1
- 1.3 Numerical Summary . . . 2
- 1.4 EDA . . . 5
- 1.5 Linear Mixed Model (VRE) . . . 5
  - 1.5.1 Model Diagnostics . . . 5
  - 1.5.2 Results . . . 11
- 1.6 Linear Mixed Model (Klebsiella) . . . 13
  - 1.6.1 Model Diagnostics . . . 13
  - 1.6.2 Results . . . 18
- 1.7 Repeated Measure ANOVA (VRE) . . . 20
  - 1.7.1 Model Diagnostics . . . 20
  - 1.7.2 Results . . . 21
- 1.8 Repeated Measure ANOVA (Klebsiella) . . . 22
  - 1.8.1 Model Diagnostics . . . 22
  - 1.8.2 Results . . . 24
  - 1.8.3 Conclusions . . . 25

1 KP and VRE Colonization after 24 days

1.1 Description

1.2 Loading the Data

Preview of Data

group	mice	day	organism	cfu
Saline	1	0	K.pneumoniae	2
Saline	2	0	K.pneumoniae	2
Saline	3	0	K.pneumoniae	2
Saline	4	0	K.pneumoniae	2
1 Day	5	0	K.pneumoniae	2
1 Day	6	0	K.pneumoniae	2
1 Day	7	0	K.pneumoniae	2
1 Day	8	0	K.pneumoniae	2
1 Day	9	0	K.pneumoniae	2
3 Day	10	0	K.pneumoniae	2

Missingness check:

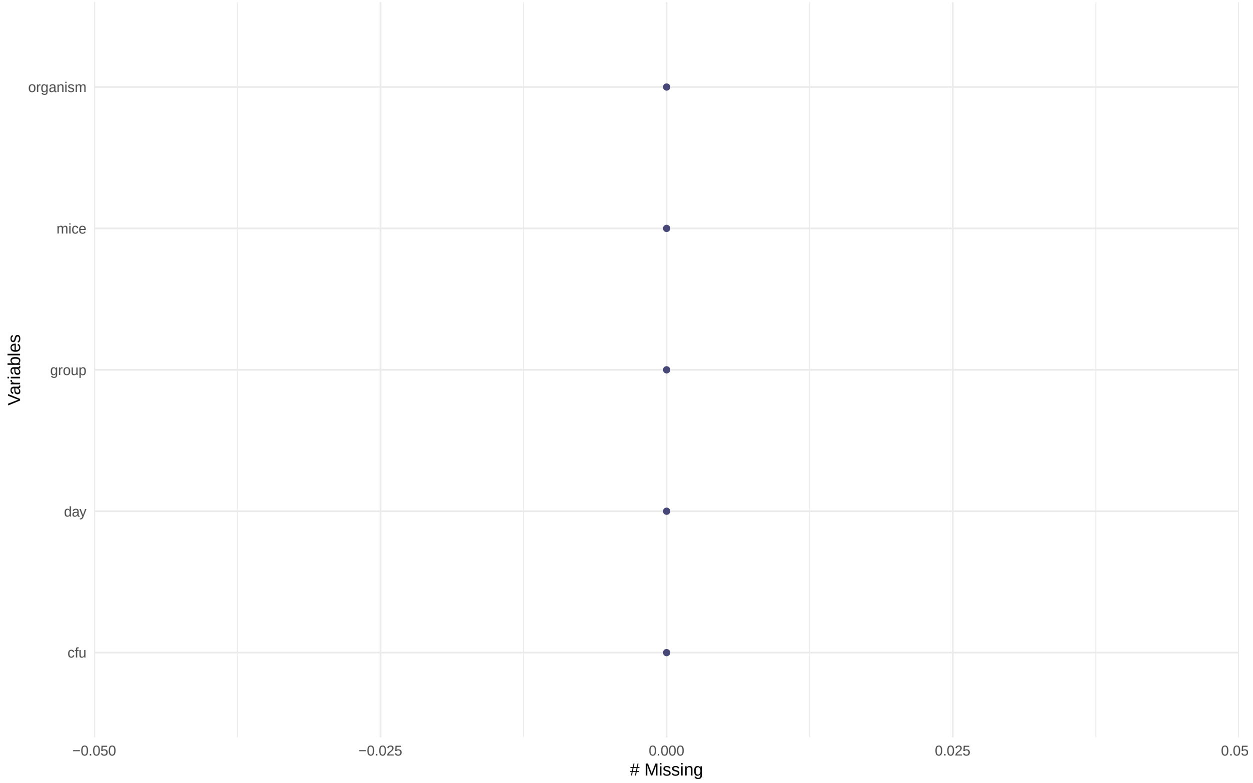

There is no missing data within the dataset.

1.3 Numerical Summary

```
## vrekp4
##
##  5  Variables      180  Observations
## --------------------------------------------------------------------------------
## group
##        n  missing distinct
##      180        0        5
##
## Value      Saline  1 Day  3 Day  6 Day 10 Day
## Frequency      24     30     30     48     48
## Proportion  0.133  0.167  0.167  0.267  0.267
## --------------------------------------------------------------------------------
## mice
##        n  missing distinct
##      180        0       30
##
## lowest : 1  2  3  4  5 , highest: 26 27 28 29 30
## --------------------------------------------------------------------------------
## day
##        n  missing distinct
##      180        0        3
##
```

```
## Value          0     1     5
## Frequency     60    60    60
## Proportion 0.333 0.333 0.333
## ---------------------------------------------------------------------------
## organism
##        n  missing distinct
##      180        0        2
##
## Value      K.pneumoniae          VRE
## Frequency            90           90
## Proportion          0.5          0.5
## ---------------------------------------------------------------------------
## cfu
##        n  missing distinct     Info     Mean      Gmd      .05      .10
##      180        0       18    0.361    2.371   0.6769    2.000    2.000
##      .25      .50      .75      .90      .95
##    2.000    2.000    2.000    3.301    4.656
##
## 2 (155, 0.861), 3 (2, 0.011), 3.30102999566398 (6, 0.033), 3.47712125471966 (3,
## 0.017), 3.69897000433602 (1, 0.006), 4.04139268515823 (1, 0.006),
## 4.47712125471966 (1, 0.006), 4.60205999132796 (1, 0.006), 4.65321251377534 (1,
## 0.006), 4.69897000433602 (1, 0.006), 5.07918124604763 (1, 0.006),
## 5.84509804001426 (1, 0.006), 6.47712125471966 (1, 0.006), 6.61278385671974 (1,
## 0.006), 6.95424250943933 (1, 0.006), 7.69897000433602 (1, 0.006),
## 7.84509804001426 (1, 0.006), 7.90308998699194 (1, 0.006)
##
## For the frequency table, variable is rounded to the nearest 0
## ---------------------------------------------------------------------------
```

Log_{10} VRE Recovered by Antibiotic Group and Day

day	n	Mean	SD	SEM	Median	IQR	Min	Max
Saline								
0	4	2.00	0.00	0.00	2.00	0.00	2.00	2.00
1	4	2.00	0.00	0.00	2.00	0.00	2.00	2.00
5	4	2.00	0.00	0.00	2.00	0.00	2.00	2.00
1 Day								
0	5	2.00	0.00	0.00	2.00	0.00	2.00	2.00
1	5	2.26	0.58	0.26	2.00	0.00	2.00	3.30
5	5	2.00	0.00	0.00	2.00	0.00	2.00	2.00
3 Day								
0	5	2.00	0.00	0.00	2.00	0.00	2.00	2.00
1	5	2.41	0.91	0.41	2.00	0.00	2.00	4.04
5	5	2.00	0.00	0.00	2.00	0.00	2.00	2.00
6 Day								
0	8	2.00	0.00	0.00	2.00	0.00	2.00	2.00
1	8	3.37	1.20	0.42	3.30	0.56	2.00	5.85
5	8	2.00	0.00	0.00	2.00	0.00	2.00	2.00
10 Day								
0	8	2.00	0.00	0.00	2.00	0.00	2.00	2.00

1	8	4.55	1.67	0.59	4.63	2.13	2.00	6.95
5	8	3.31	2.44	0.86	2.00	1.15	2.00	7.85

Log_{10} ***K.pneumoniae*** Recovered by Antibiotic Group and Day

day	n	Mean	SD	SEM	Median	IQR	Min	Max
Saline								
0	4	2.00	0.00	0.00	2.00	0.00	2.00	2.00
1	4	2.00	0.00	0.00	2.00	0.00	2.00	2.00
5	4	2.00	0.00	0.00	2.00	0.00	2.00	2.00
1 Day								
0	5	2.00	0.00	0.00	2.00	0.00	2.00	2.00
1	5	2.00	0.00	0.00	2.00	0.00	2.00	2.00
5	5	2.00	0.00	0.00	2.00	0.00	2.00	2.00
3 Day								
0	5	2.00	0.00	0.00	2.00	0.00	2.00	2.00
1	5	2.00	0.00	0.00	2.00	0.00	2.00	2.00
5	5	2.00	0.00	0.00	2.00	0.00	2.00	2.00
6 Day								
0	8	2.00	0.00	0.00	2.00	0.00	2.00	2.00
1	8	2.00	0.00	0.00	2.00	0.00	2.00	2.00
5	8	2.00	0.00	0.00	2.00	0.00	2.00	2.00
10 Day								
0	8	2.00	0.00	0.00	2.00	0.00	2.00	2.00
1	8	2.49	0.70	0.25	2.00	1.12	2.00	3.48
5	8	4.22	2.46	0.87	3.74	3.45	2.00	7.90

1.4 EDA

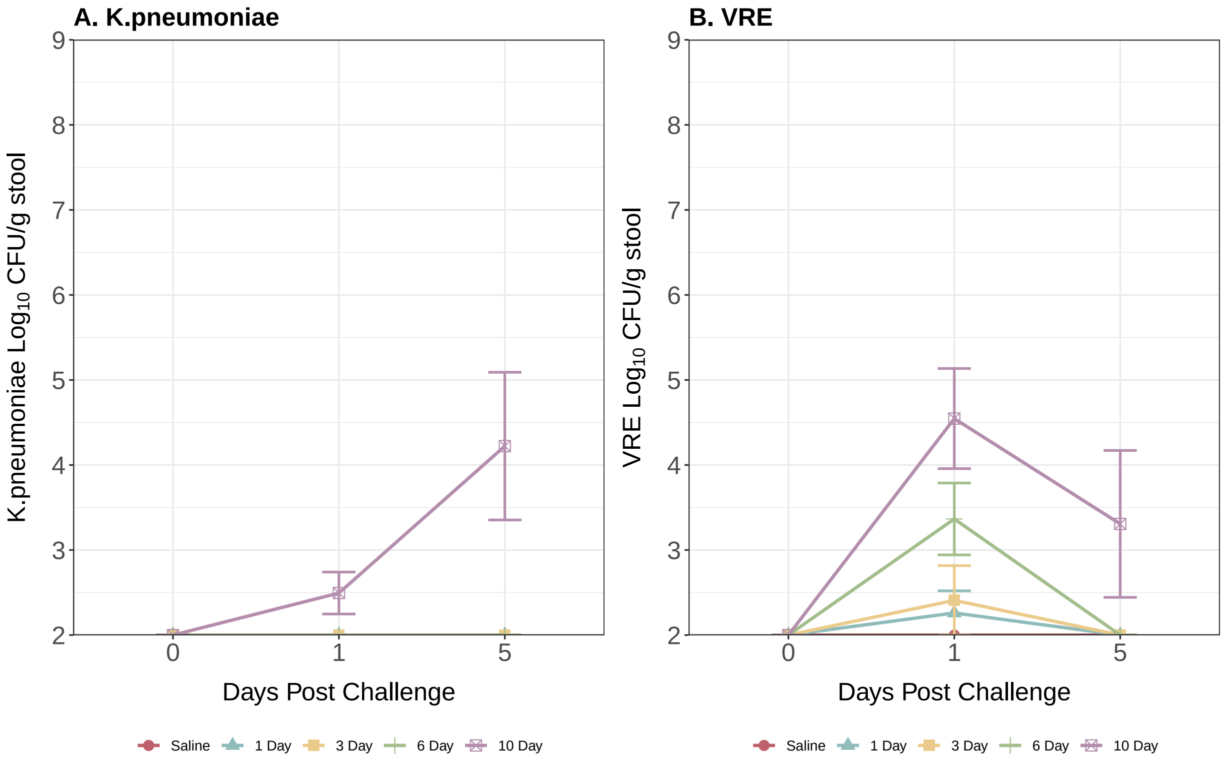

1.5 Linear Mixed Model (VRE)

1.5.1 Model Diagnostics

The linear mixed model has the following assumptions:

- **Linearity** – The expected CFU change is a linear function of the fixed effects (`group`, `day`, and `group` x `day`). Nonlinear function will result in biased estimates
- **Normality of Level-1 Residuals** – After accounting for the fixed effects and the random intercept, the remaining (within-in mouse) errors should be normal. This ensures proper p-values and CIs
- **Homoscedasticty within Groups** – Within each antibiotic `group`, the residual variance should be constant. (This is addressed using the constant variance function within the model which assigns group-specific variances)
- **Independence of Residuals** – For the random intercepts in the model, the residuals should be uncorrelated both within and between mice (The within mouse variance is captured with the random slope)
- **Normality of Random Effects** – The mouse-specific intercepts are assumed to follow a normal distribution
- **Correct Random-Effects Structure** – The within-mouse correlation is captured by a single intercept (instead of random slopes). If mice differ in how quickly they clear VRE omitting a random slope could bias the fixed-effects SEs
- **No Perfect Multicollinearity** – The predictors must not be highly correlated with one another. Collinearity can inflate SEs and make hypothesis tests unreliable
- **Properly Nested Data** – Each observation must belong to one mice, and the clustering factor (`mice`) must be uniquely identify independent sampling units

1.5.1.1 Linearity

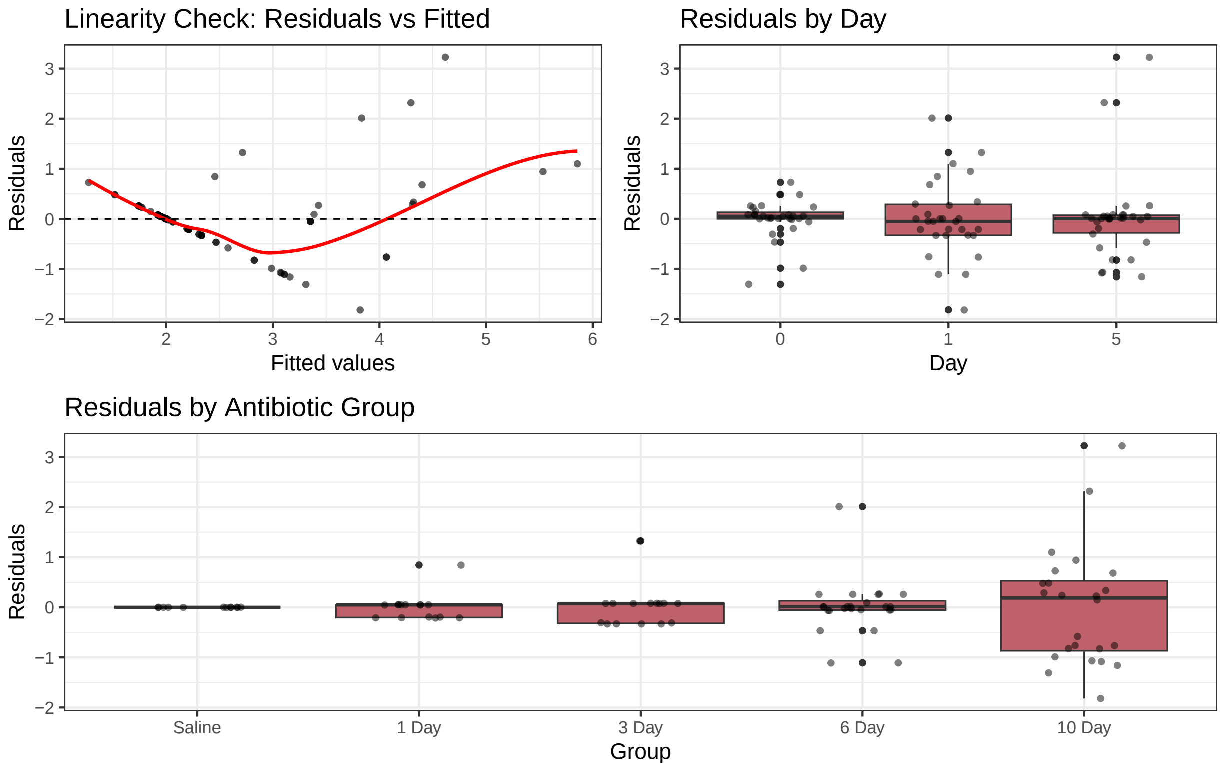

1.5.1.2 Normality of Level-1 Residuals

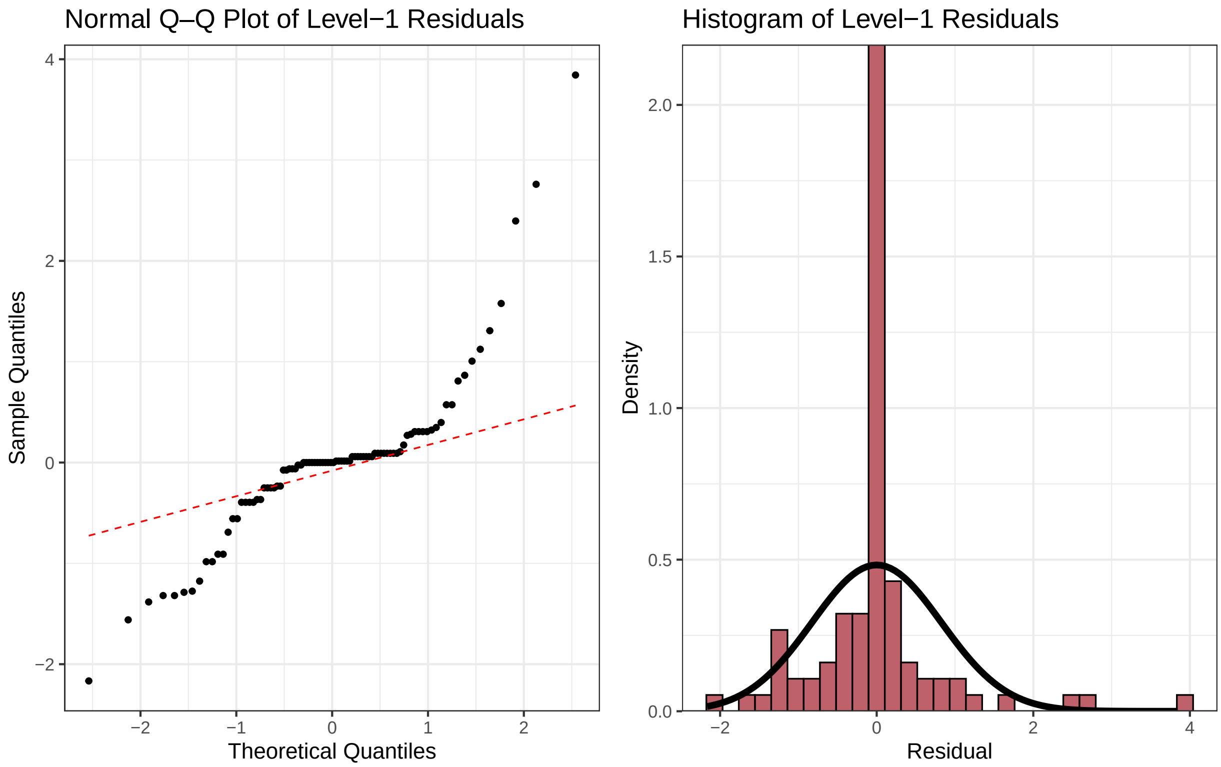


```
##
##  Shapiro-Wilk normality test
##
## data:  diag_df$resid
## W = 0.82737, p-value = 7.271e-09
```

1.5.1.3 Homoscedasticity within Groups

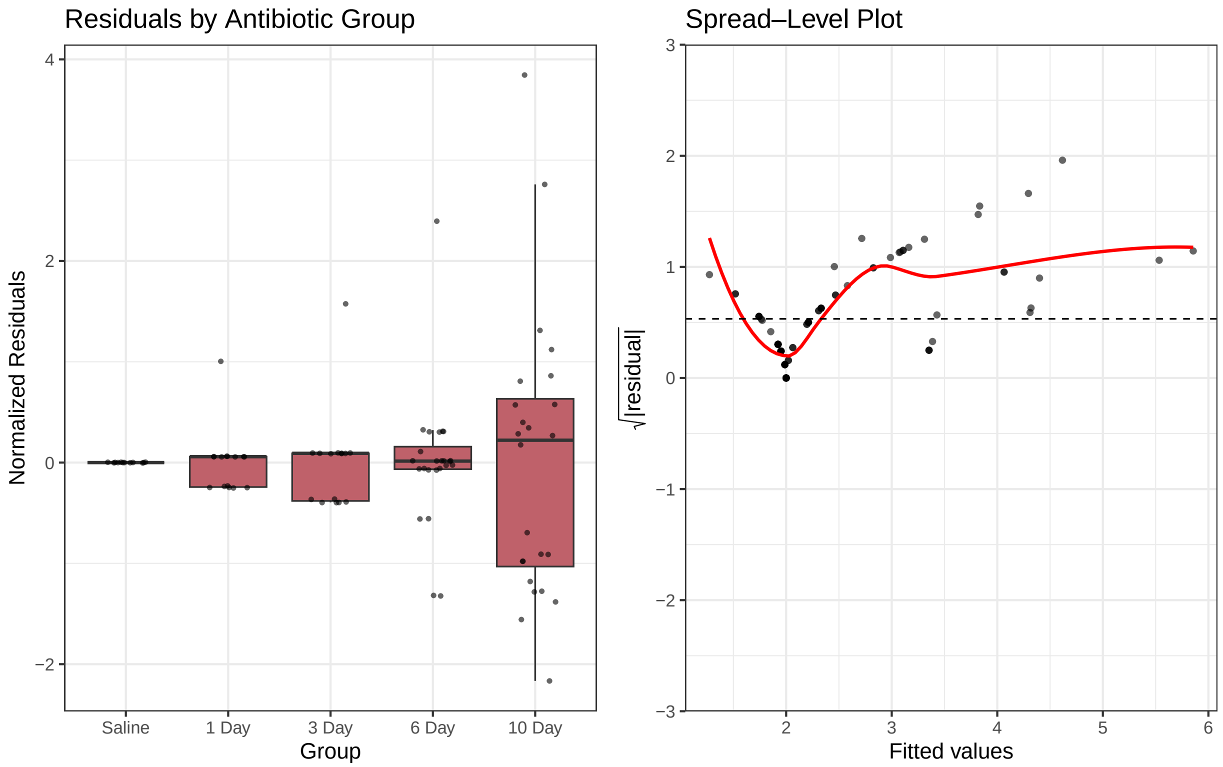


```
## Levene's Test for Homogeneity of Variance (center = median)
##       Df F value    Pr(>F)
## group  4  10.435 6.347e-07 ***
##       85
## ---
## Signif. codes:  0 '***' 0.001 '**' 0.01 '*' 0.05 '.' 0.1 ' ' 1
```

1.5.1.4 Independence of Residuals

An ACF plot is used to represent the autocorrelation of time series data, to show how strongly data correlates with past values at different intervals (lags)

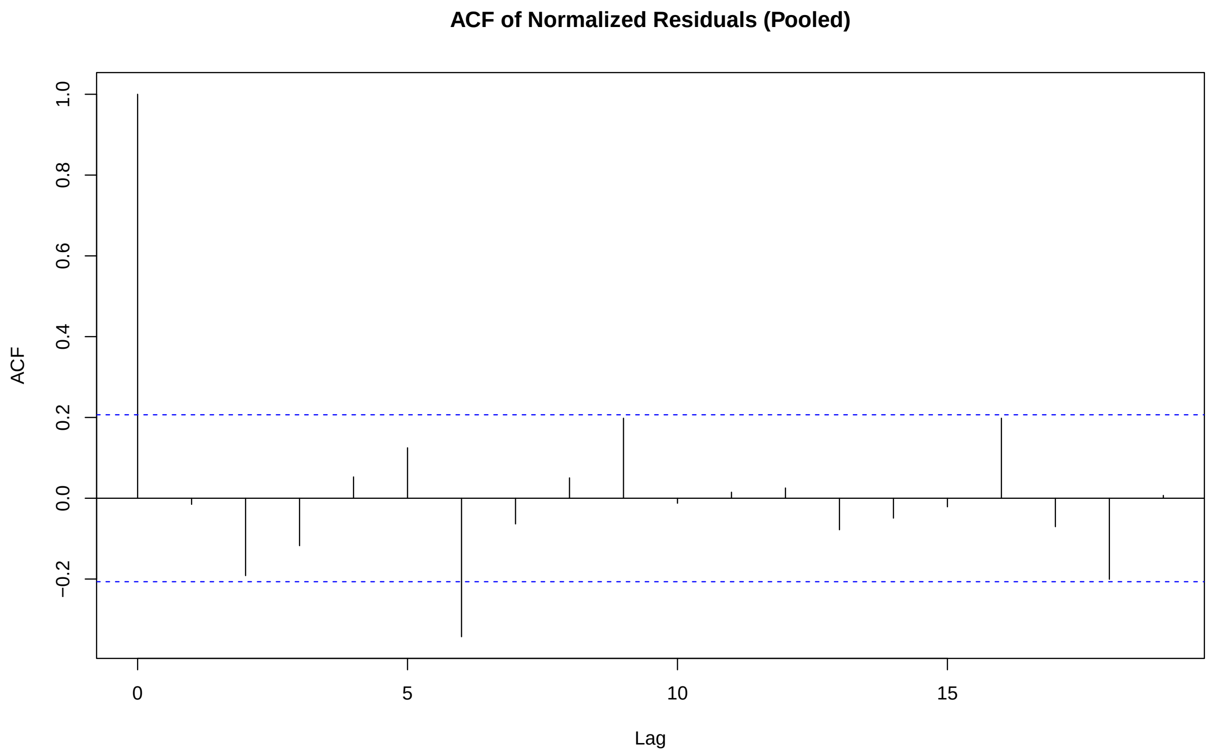
ACF of Normalized Residuals (Pooled)
ACF
1.0
0.8
0.6
0.4
0.2
0.0
−0.2
0
5
10
15
Lag

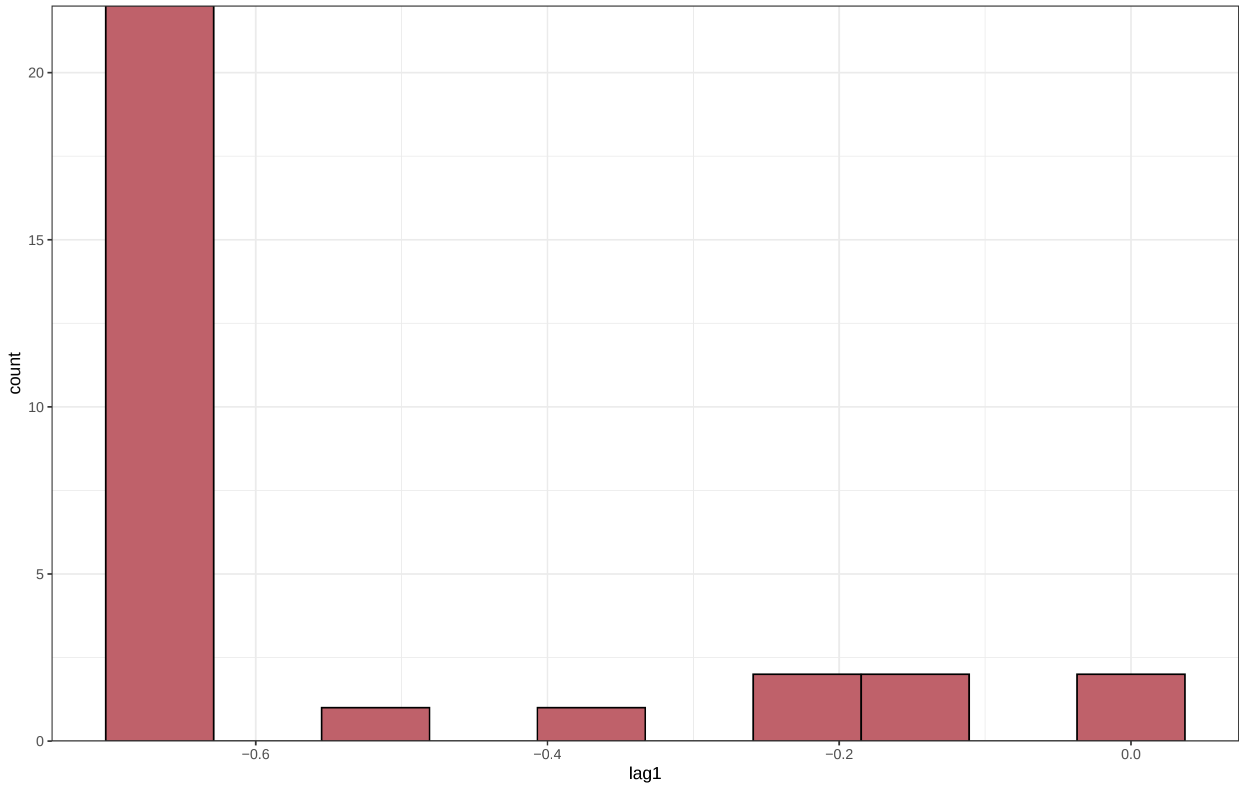
count
20
15
10
5
0
−0.6
−0.4
−0.2
0.0
lag1

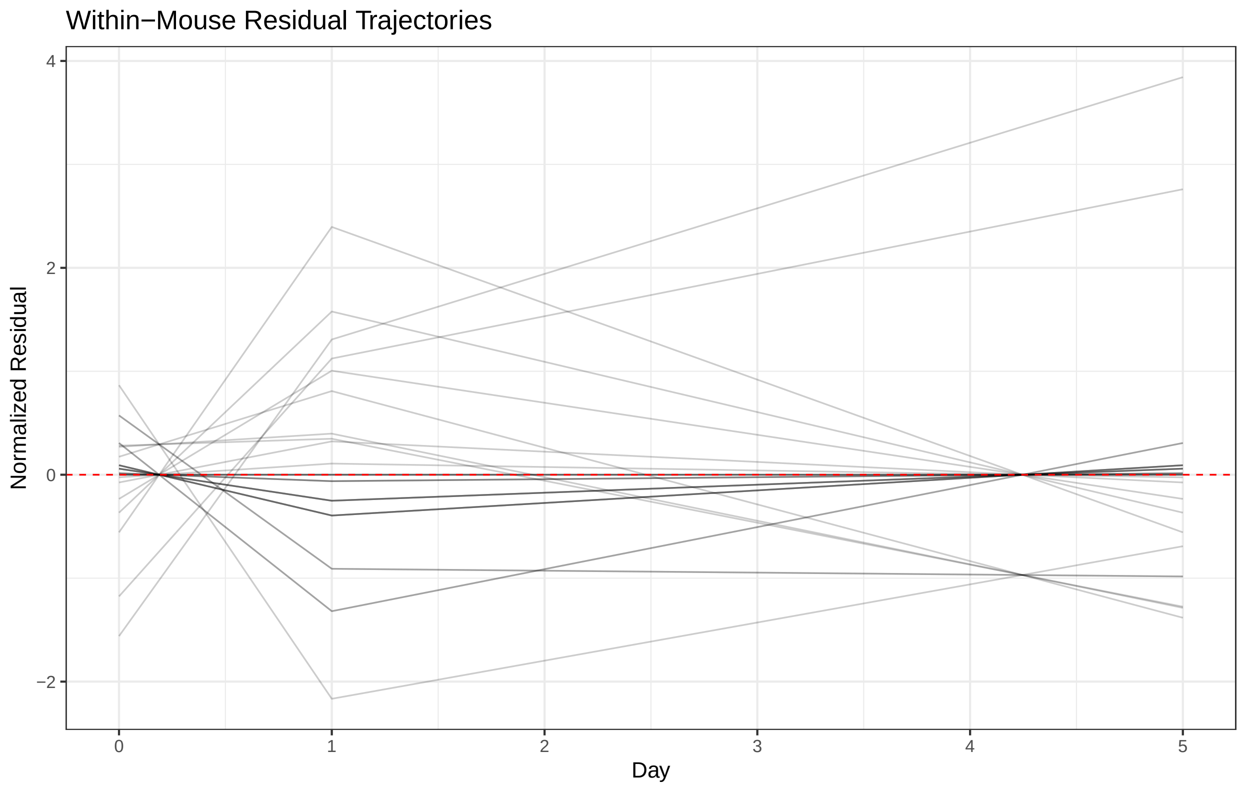

1.5.1.5 Normality of Random Effects

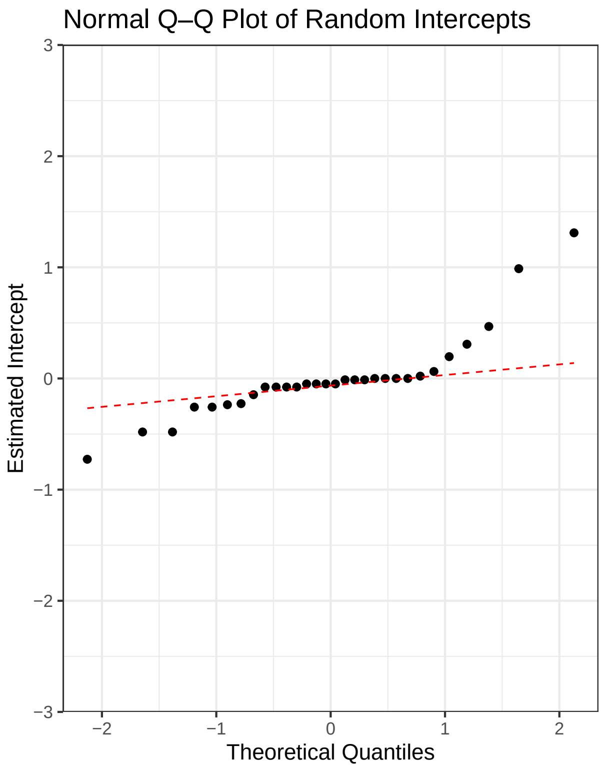

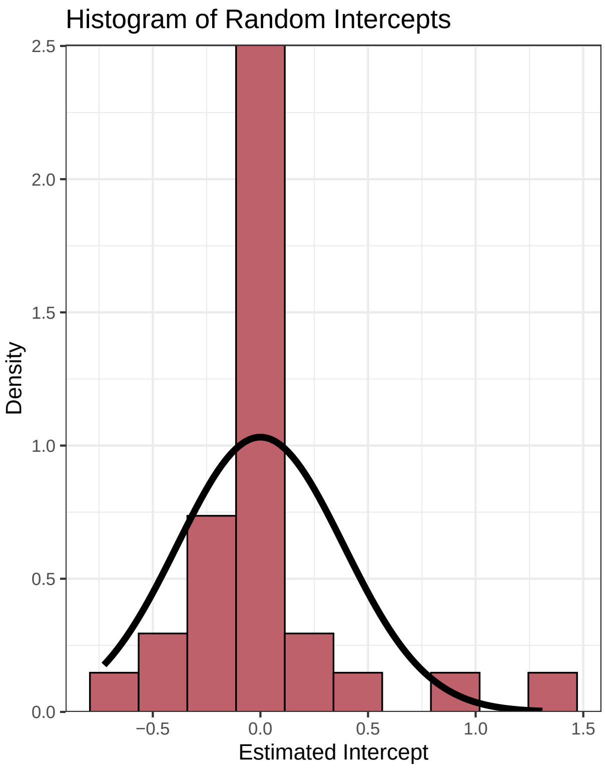

```
##
##  Shapiro-Wilk normality test
##
## data:  rand_eff$intercept
## W = 0.80159, p-value = 7.009e-05
```

1.5.1.6 Correct Random-Effects Structure

```
## mice = pdLogChol(1)
##             Variance  StdDev
## (Intercept) 0.3064590 0.5535874
## Residual    0.7053213 0.8398341
```

```
## [1] 0.3028909
```

1.5.1.7 No Perfect Multicollinearity

```
##               GVIF Df GVIF^(1/(2*Df))
## group        81.00  4        1.732051
## day          56.25  2        2.738613
## group:day 1923.75  8        1.604203
```

The VIF terms are very large (where values greater than 10 are considered severe issues) but this is an unadjusted inflation factor on multi-df terms. The GVIF^2 terms provide a reasonable estimate indicating that we have no major issues with collinearity.

1.5.1.8 Properly Nested Data All `mice` ID appear in exactly one group (no mouse switches between antibiotic conditions) and each mice has the same number of observations (one per level of `day`), so the repeated-measures structure is balanced and nested. This indicates our data is properly nested for a random-intercept model.

1.5.2 Results

Model Fit Statistics

Statistic	Value
AIC	267.81
BIC	307.21
logLik	−116.91

Random-Effects Variance Components

	Variance	StdDev
(Intercept)	0.306	0.554
Residual	0.705	0.840

Fixed Effects Estimates – Linear Mixed Model

Modeling CFU by group x day x organism

Term	Estimate	SE	Lower 95% CI	Upper 95% CI	z	p-value
(Intercept)	2.00	0.50	0.99	3.01	3.98	0.000

1 Day Abx Day	0.00	0.67	−1.39	1.39	0.00	1.000
6 Day Abx Day	0.00	0.67	−1.39	1.39	0.00	1.000
NA Day	0.00	0.62	−1.27	1.27	0.00	1.000
NA Day	0.00	0.62	−1.27	1.27	0.00	1.000
1	0.00	0.59	−1.19	1.19	0.00	1.000
NA	0.00	0.59	−1.19	1.19	0.00	1.000
1 Day Abx Day × 1	0.26	0.80	−1.34	1.86	0.33	0.745
6 Day Abx Day × 1	0.41	0.80	−1.19	2.01	0.51	0.611
NA Day × 1	1.37	0.73	−0.10	2.83	1.88	0.066
NA Day × 1	2.55	0.73	1.09	4.01	3.50	0.001
1 Day Abx Day × NA	0.00	0.80	−1.60	1.60	0.00	1.000
6 Day Abx Day × NA	0.00	0.80	−1.60	1.60	0.00	1.000
NA Day × NA	0.00	0.73	−1.46	1.46	0.00	1.000
NA Day × NA	1.31	0.73	−0.15	2.77	1.80	0.078

Dunnett Post-hoc Comparisons

Each antibiotic group vs. saline at each post-treatment day for VRE

	Estimate	SE	DF	Lower 95% CI	Upper 95% CI	t-ratio	p-value
0 Day Post-Treatment							
1 Day - Saline	0.00	0.67	64	−1.73	1.73	0.00	1.000
3 Day - Saline	0.00	0.67	64	−1.73	1.73	0.00	1.000
6 Day - Saline	0.00	0.62	64	−1.58	1.58	0.00	1.000
10 Day - Saline	0.00	0.62	64	−1.58	1.58	0.00	1.000
1 Day Post-Treatment							
1 Day - Saline	0.26	0.67	64	−1.47	1.99	0.39	1.000
3 Day - Saline	0.41	0.67	64	−1.33	2.14	0.61	1.000
6 Day - Saline	1.37	0.62	64	−0.22	2.95	2.22	0.091
10 Day - Saline	2.55	0.62	64	0.96	4.13	4.13	0.000
5 Day Post-Treatment							
1 Day - Saline	0.00	0.67	64	−1.73	1.73	0.00	1.000
3 Day - Saline	0.00	0.67	64	−1.73	1.73	0.00	1.000
6 Day - Saline	0.00	0.62	64	−1.58	1.58	0.00	1.000
10 Day - Saline	1.31	0.62	64	−0.28	2.89	2.12	0.151

To evaluate how antibiotic duration influences *vancomycin-resistant Enterococcus* (VRE) colonization over time, Dunnett-adjusted pairwise comparisons between each antibiotic group and the saline control at 1,and 5 days post challenge. One day after inoculation with VRE, there were no significant differences with the 1-day and 3-day antibiotic groups ($p > 0.99$). The 6-day antibiotic group showed an non-significant increase in VRE stool concentration with an average of 1.37 $\log_{10}$ CFU/g (95% CI [-0.22, 2.95]; $p = 0.091$). The 10 day antibiotic treatment group had the largest significant colonization of VRE with an average of 2.55 $\log_{10}$ CFU/g (95% CI [0.96, 4.13]; $p < 0.001$) in comparison to the saline group. Five days after colonization with VRE, the 1, 3, and 6 day antibiotics were non-significantly different from saline group. The 10-day antibiotic group had an average concentration of 1.31 $\log_{10}$ CFU/g (95% CI [-0.28, 2.89]) but was not significantly different from the control group. These results suggest that antibiotic treatment durations greater than 6 days are likely to make one individual more prone to a VRE infection, with the 10-day antibiotic group being the most susceptible.

Overall Group Comparisons

Comparisons of overall differences for antibiotics group in colonization

	Estimate	SE	DF	Lower 95% CI	Upper 95% CI	t-ratio	p-value
Saline - 1 Day	−0.09	0.49	25	−1.61	1.43	−0.18	1.000
Saline - 3 Day	−0.14	0.49	25	−1.66	1.39	−0.28	1.000
Saline - 6 Day	−0.46	0.45	25	−1.84	0.93	−1.01	1.000
Saline - 10 Day	−1.28	0.45	25	−2.67	0.10	−2.85	0.086
1 Day - 3 Day	−0.05	0.47	25	−1.48	1.39	−0.11	1.000
1 Day - 6 Day	−0.37	0.42	25	−1.66	0.92	−0.88	1.000
1 Day - 10 Day	−1.20	0.42	25	−2.49	0.10	−2.85	0.086
3 Day - 6 Day	−0.32	0.42	25	−1.61	0.97	−0.76	1.000
3 Day - 10 Day	−1.15	0.42	25	−2.44	0.14	−2.74	0.091
6 Day - 10 Day	−0.83	0.37	25	−1.96	0.30	−2.25	0.234

Pairwise comparisons between all antibiotics groups found no significant differences (all $p > 0.085$)

1.6 Linear Mixed Model (Klebsiella)

1.6.1 Model Diagnostics

The linear mixed model has the following assumptions:

- **Linearity** – The expected CFU change is a linear function of the fixed effects (`group`, `day`, and `group` x `day`). Nonlinear function will result in biased estimates
- **Normality of Level-1 Residuals** – After accounting for the fixed effects and the random intercept, the remaining (within-in mouse) errors should be normal. This ensures proper *p*-values and CIs
- **Homoscedasticty within Groups** – Within each antibiotic `group`, the residual variance should be constant. (This is addressed using the constant variance function within the model which assigns group-specific variances)
- **Independence of Residuals** – For the random intercepts in the model, the residuals should be uncorrelated both within and between mice (The within mouse variance is captured with the random slope)
- **Normality of Random Effects** – The mouse-specific intercepts are assumed to follow a normal distribution
- **Correct Random-Effects Structure** – The within-mouse correlation is captured by a single intercept (instead of random slopes). If mice differ in how quickly they clear *Klebsiella* omitting a random slope could bias the fixed-effects SEs
- **No Perfect Multicollinearity** – The predictors must not be highly correlated with one another. Collinearity can inflate SEs and make hypothesis tests unreliable
- **Properly Nested Data** – Each observation must belong to one mice, and the clustering factor (`mice`) must be uniquely identify independent sampling units

1.6.1.1 Linearity

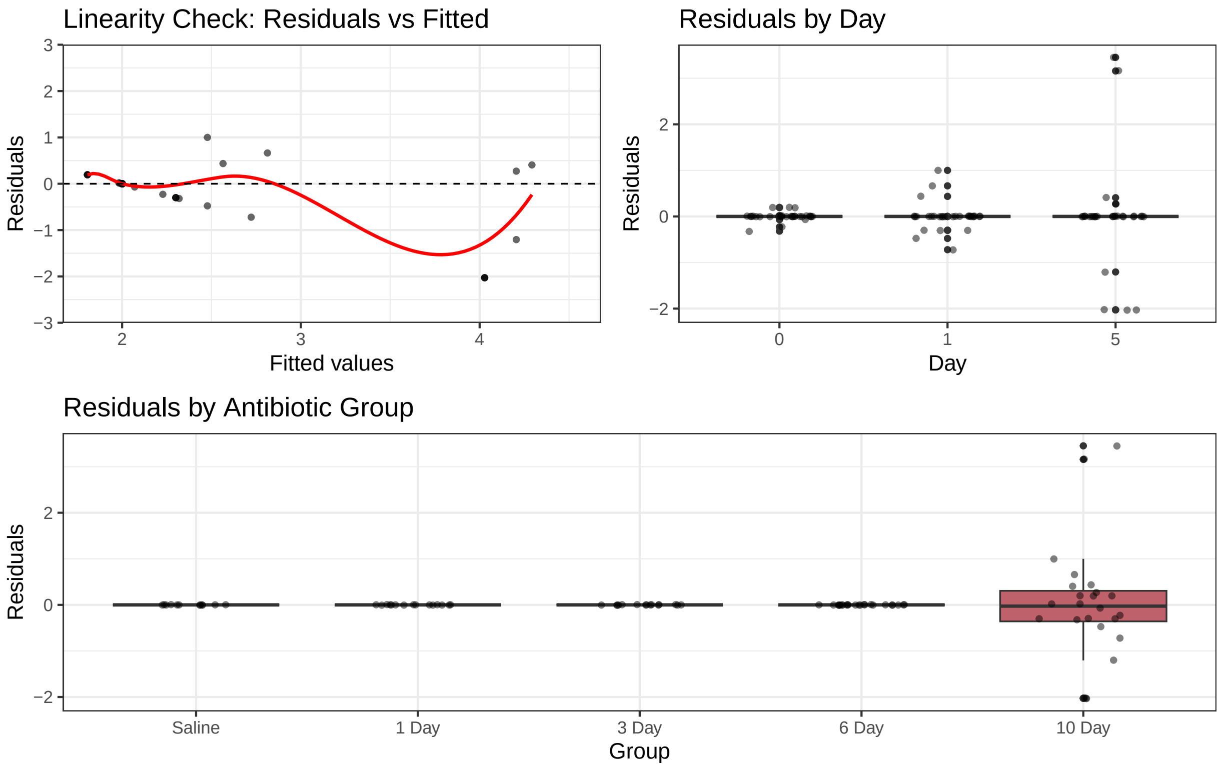

1.6.1.2 Normality of Level-1 Residuals

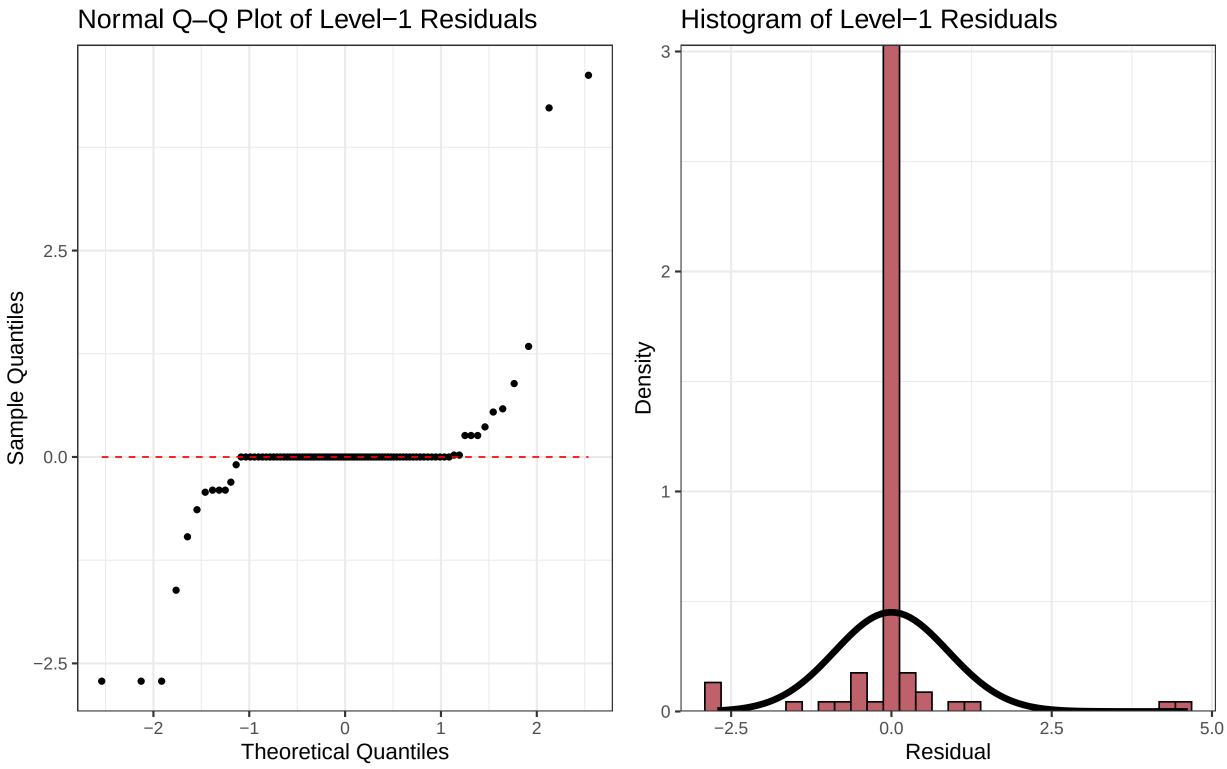

```
##
##  Shapiro-Wilk normality test
##
## data:  diag_df$resid
## W = 0.48779, p-value = 3.646e-16
```

1.6.1.3 Homoscedasticity within Groups

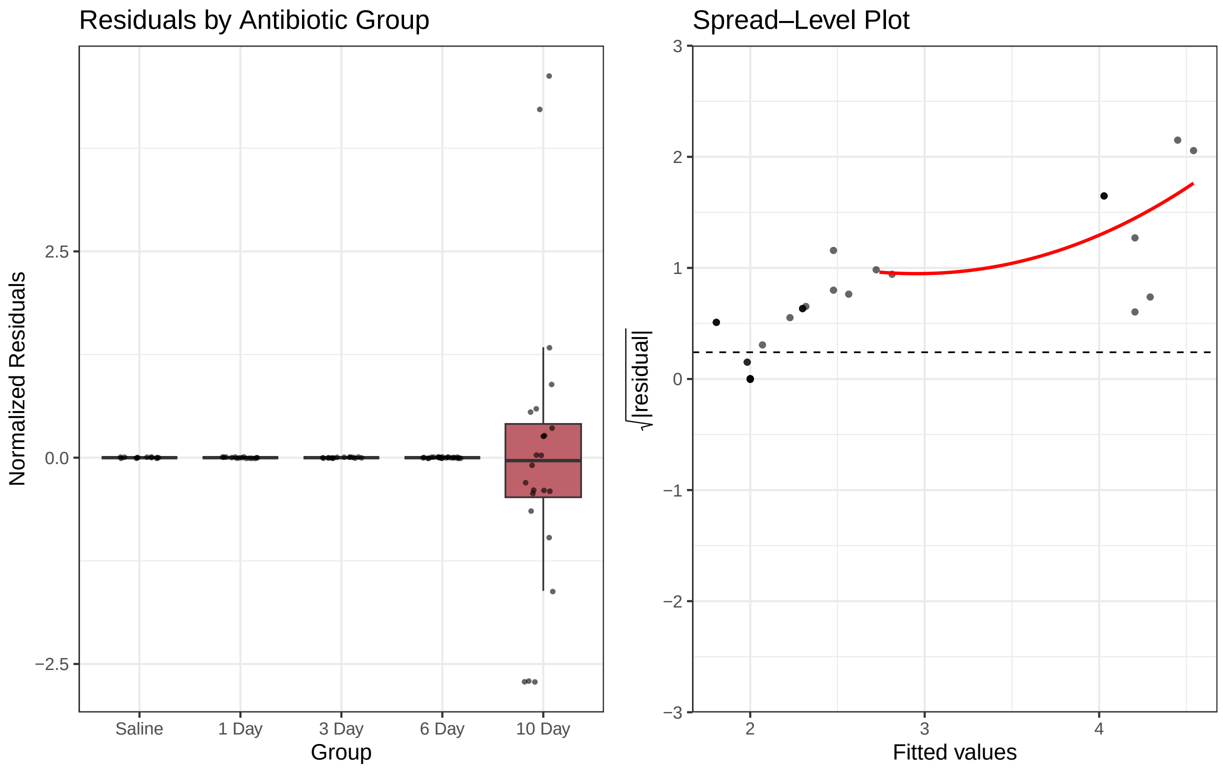


```
## Levene's Test for Homogeneity of Variance (center = median)
##       Df F value    Pr(>F)
## group  4  11.723 1.254e-07 ***
##       85
## ---
## Signif. codes:  0 '***' 0.001 '**' 0.01 '*' 0.05 '.' 0.1 ' ' 1
```

1.6.1.4 Independence of Residuals An ACF plot is used to represent the autocorrelation of time series data, to show how strongly data correlates with past values at different intervals (lags)

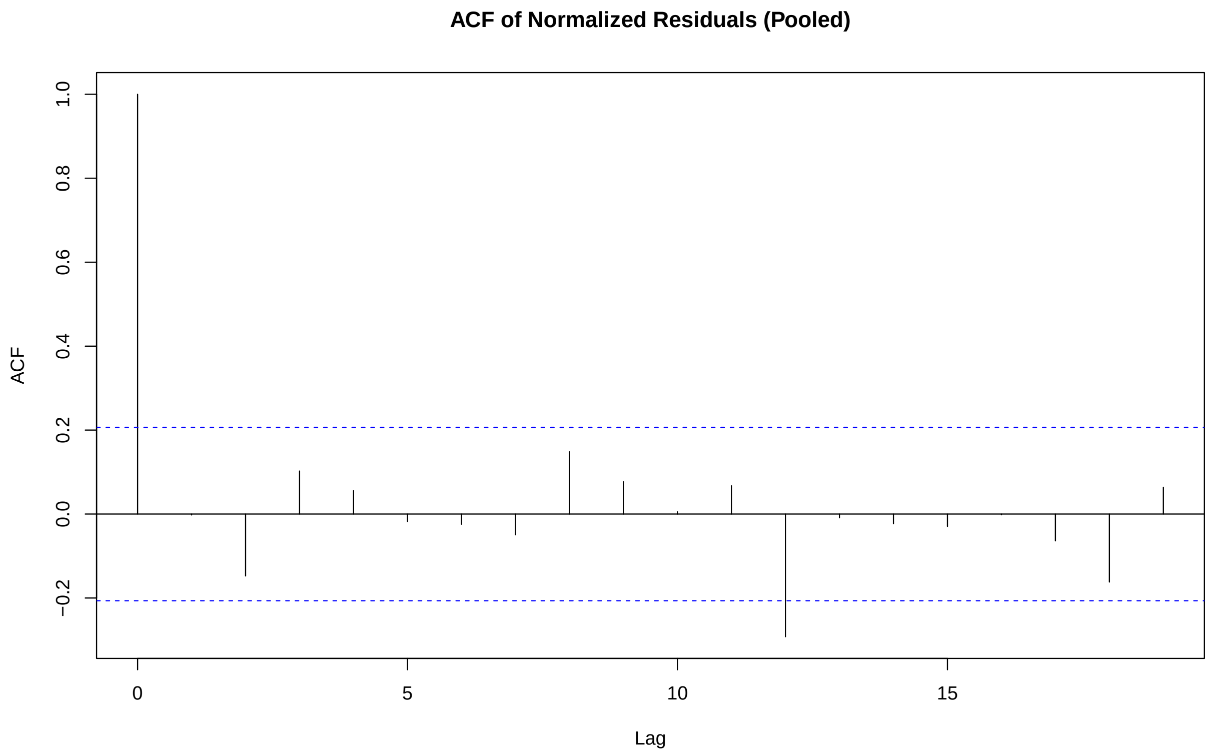
ACF of Normalized Residuals (Pooled)
ACF
1.0
0.8
0.6
0.4
0.2
0.0
−0.2
0
5
10
15
Lag

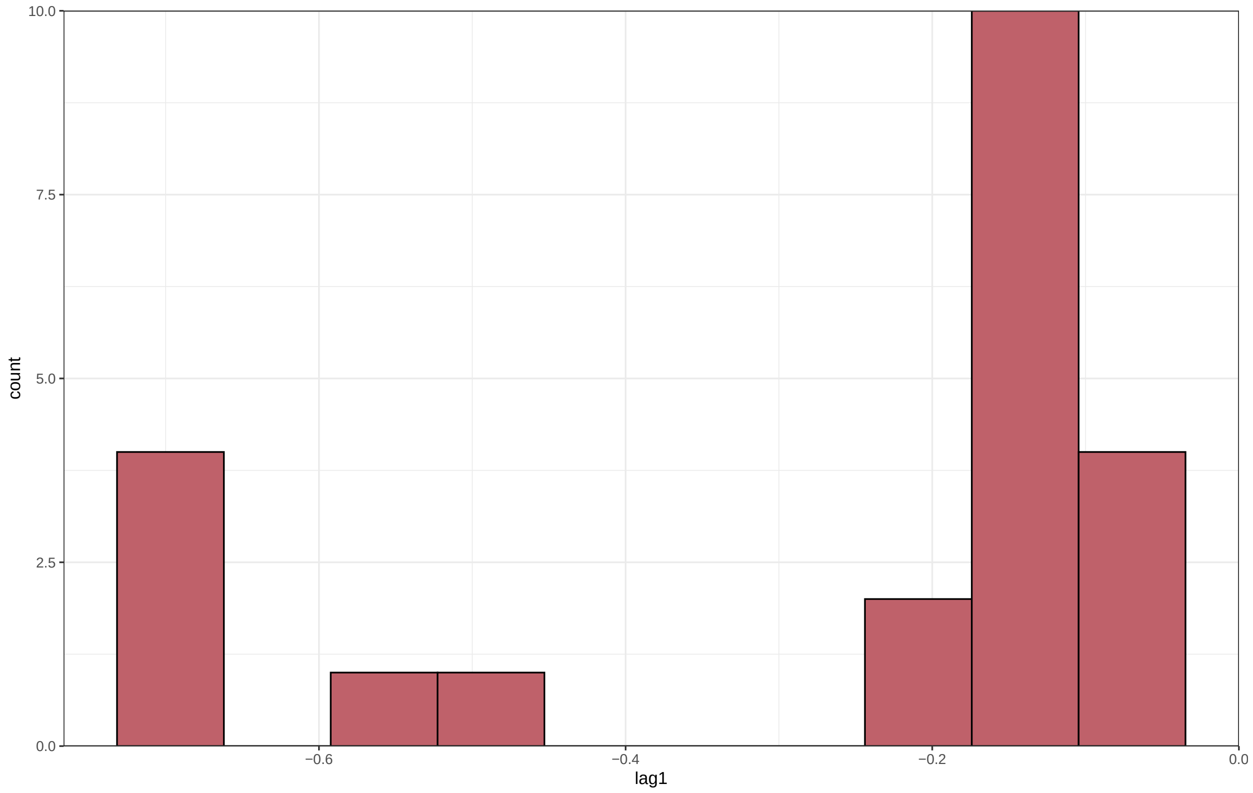
count
10.0
7.5
5.0
2.5
0.0
−0.6
−0.4
−0.2
0.0
lag1

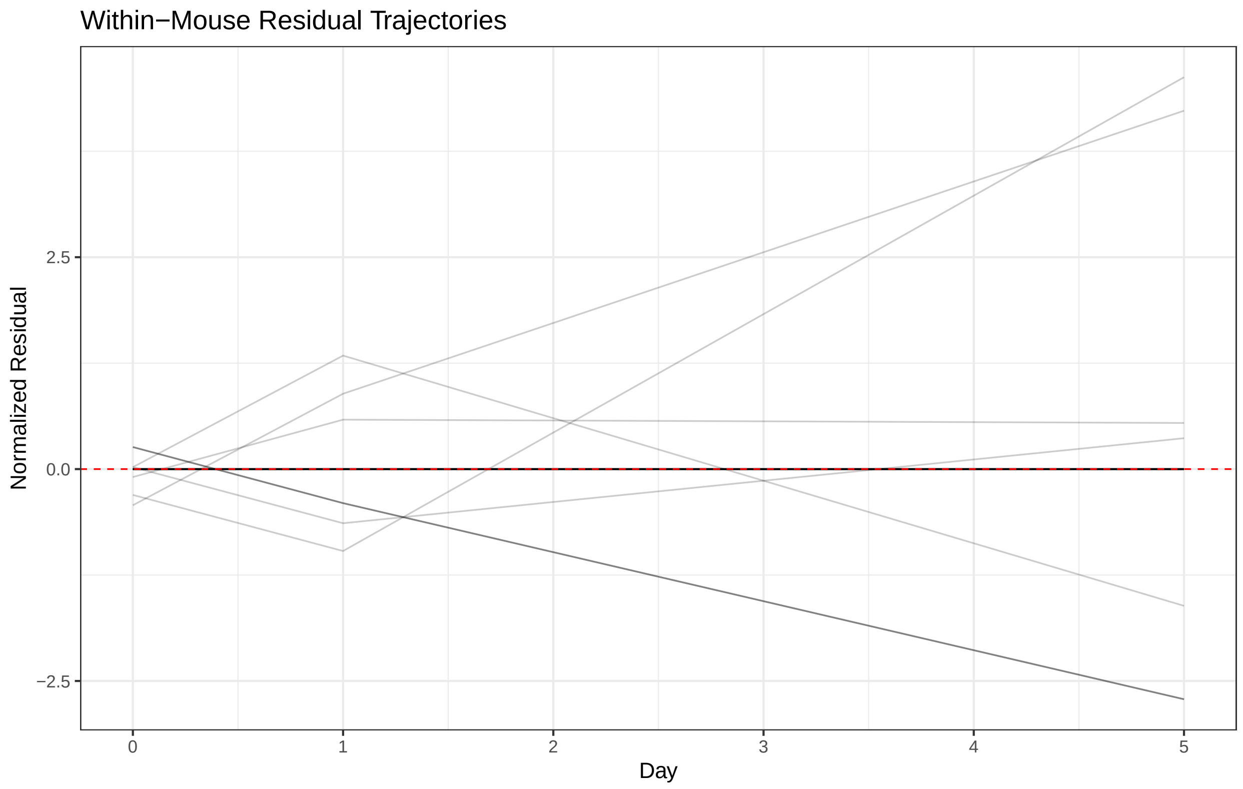

1.6.1.5 Normality of Random Effects

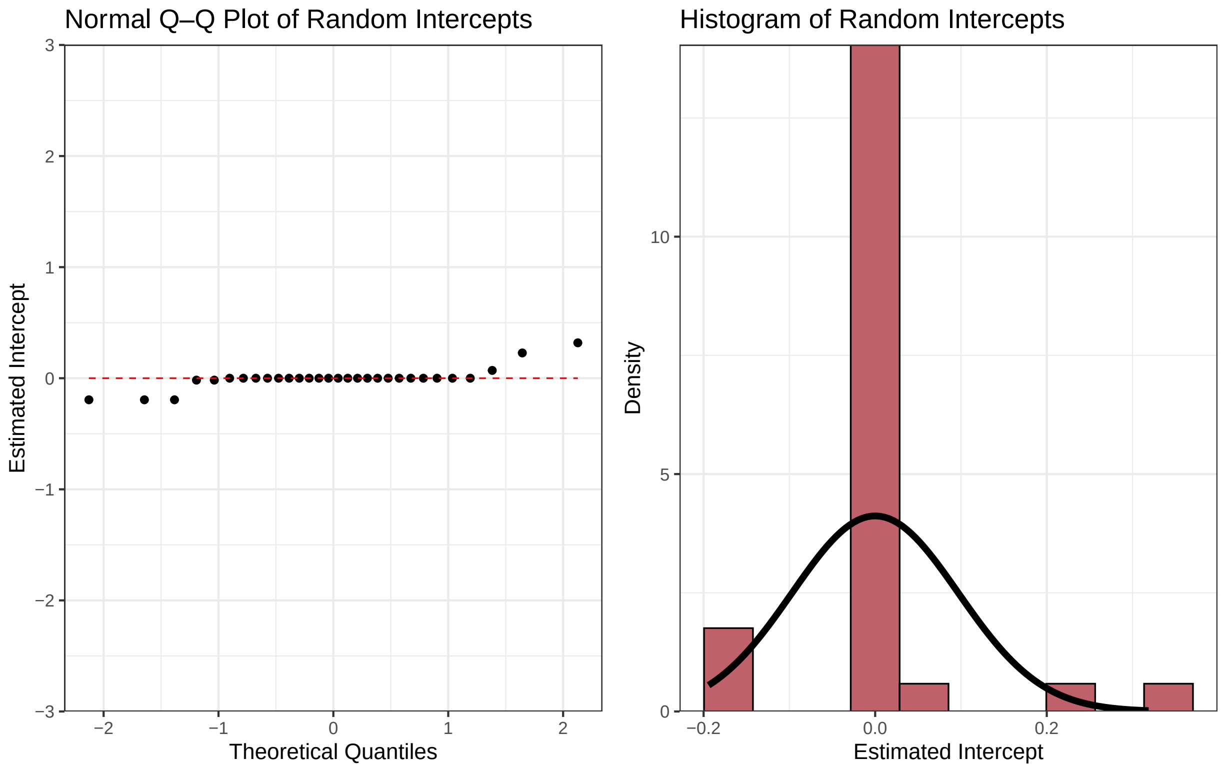

1.6.1.6 Correct Random-Effects Structure

```
## mice = pdLogChol(1)
##             Variance   StdDev
## (Intercept) 0.05075696 0.2252930
## Residual    0.55786620 0.7469044

## [1] 0.08339633
```

1.6.1.7 No Perfect Multicollinearity

```
##               GVIF Df GVIF^(1/(2*Df))
## group        81.00  4        1.732051
## day          56.25  2        2.738613
## group:day  1923.75  8        1.604203
```

The VIF terms are very large (where values greater than 10 are considered severe issues) but this is an unadjusted inflation factor on multi-df terms. The $GVIF^2$ terms provide a reasonable estimate indicating that we have no major issues with collinearity.

1.6.1.8 Properly Nested Data All `mice` ID appear in exactly one group (no mouse switches between antibiotic conditions) and each mice has the same number of observations (one per level of `day`), so the repeated-measures structure is balanced and nested. This indicates our data is properly nested for a random-intercept model.

1.6.2 Results

Model Fit Statistics

Statistic	Value
AIC	235.39
BIC	274.79
logLik	−100.70

Random-Effects Variance Components

	Variance	StdDev
(Intercept)	0.051	0.225
Residual	0.558	0.747

Fixed Effects Estimates – Linear Mixed Model

Modeling CFU by group x day x organism

Term	Estimate	SE	Lower 95% CI	Upper 95% CI	z	p-value
(Intercept)	2.00	0.39	1.22	2.78	5.13	0.000
1 Day Abx Day	0.00	0.52	−1.08	1.08	0.00	1.000
6 Day Abx Day	0.00	0.52	−1.08	1.08	0.00	1.000
NA Day	0.00	0.48	−0.98	0.98	0.00	1.000
NA Day	0.00	0.48	−0.98	0.98	0.00	1.000
1	0.00	0.53	−1.06	1.06	0.00	1.000
NA	0.00	0.53	−1.06	1.06	0.00	1.000
1 Day Abx Day × 1	0.00	0.71	−1.42	1.42	0.00	1.000

6 Day Abx Day × 1	0.00	0.71	−1.42	1.42	0.00	1.000
NA Day × 1	0.00	0.65	−1.30	1.30	0.00	1.000
NA Day × 1	0.49	0.65	−0.80	1.79	0.76	0.448
1 Day Abx Day × NA	0.00	0.71	−1.42	1.42	0.00	1.000
6 Day Abx Day × NA	0.00	0.71	−1.42	1.42	0.00	1.000
NA Day × NA	0.00	0.65	−1.30	1.30	0.00	1.000
NA Day × NA	2.22	0.65	0.92	3.52	3.44	0.001

Dunnett Post-hoc Comparisons

Each antibiotic group vs. saline at each post-treatment day for VRE

	Estimate	SE	DF	Lower 95% CI	Upper 95% CI	t-ratio	p-value
0 Day Post-Treatment							
1 Day - Saline	0.00	0.52	80	−1.34	1.34	0.00	1.000
3 Day - Saline	0.00	0.52	80	−1.34	1.34	0.00	1.000
6 Day - Saline	0.00	0.48	80	−1.22	1.22	0.00	1.000
10 Day - Saline	0.00	0.48	80	−1.22	1.22	0.00	1.000
1 Day Post-Treatment							
1 Day - Saline	0.00	0.52	80	−1.34	1.34	0.00	1.000
3 Day - Saline	0.00	0.52	80	−1.34	1.34	0.00	1.000
6 Day - Saline	0.00	0.48	80	−1.22	1.22	0.00	1.000
10 Day - Saline	0.49	0.48	80	−0.73	1.72	1.03	1.000
5 Day Post-Treatment							
1 Day - Saline	0.00	0.52	80	−1.34	1.34	0.00	1.000
3 Day - Saline	0.00	0.52	80	−1.34	1.34	0.00	1.000
6 Day - Saline	0.00	0.48	80	−1.22	1.22	0.00	1.000
10 Day - Saline	2.22	0.48	80	1.00	3.44	4.65	0.000

To evaluate how antibiotic duration influences *Klebsiella* colonization over time, Dunnett-adjusted pairwise comparisons between each antibiotic group and the saline control at 1 and 5 days post challenge. One day after inoculation with *Klebsiella*, there were no significant differences in comparison to the saline control group for any of the antibiotic groups (all $p > 0.99$). By day 5, the 1, 3, and 6 antibiotic groups continued to remain non-significantly different from the controls, while the 10 day antibiotic group had a significantly large increase in *Klebsiella* stool concentration ($\Delta = 2.22$; 95% CI [1.00, 3.45]; $p < 0.001$). These results suggest that antibiotic durations longer than 10 days, allow for an mice to be a greater risk of *Klebsiella* infection.

Overall Group Comparisons (Klebsiella)

Comparisons of overall differences for antibiotics group in colonization

	Estimate	SE	DF	Lower 95% CI	Upper 95% CI	t-ratio	p-value
Saline - 1 Day	0.00	0.33	25	−1.01	1.01	0.00	1.000
Saline - 3 Day	0.00	0.33	25	−1.01	1.01	0.00	1.000
Saline - 6 Day	0.00	0.30	25	−0.92	0.92	0.00	1.000
Saline - 10 Day	−0.91	0.30	25	−1.82	0.01	−3.04	0.039
1 Day - 3 Day	0.00	0.31	25	−0.95	0.95	0.00	1.000
1 Day - 6 Day	0.00	0.28	25	−0.85	0.85	0.00	1.000
1 Day - 10 Day	−0.91	0.28	25	−1.76	−0.05	−3.26	0.029
3 Day - 6 Day	0.00	0.28	25	−0.85	0.85	0.00	1.000
3 Day - 10 Day	−0.91	0.28	25	−1.76	−0.05	−3.26	0.029

6 Day - 10 Day	−0.91	0.24	25	−1.65	−0.16	−3.72	0.010

Pairwise comparisons across all groups found that there was a significant difference in *Klebsiella concentration* between all antibiotic and controls with the 10-day antibiotic group (all $p < 0.04$).

1.7 Repeated Measure ANOVA (VRE)

1.7.1 Model Diagnostics

The repeated measures ANOVA has the following assumptions:

- **Independence of subjects** - Each mouse's response is independent of every other's
- **Normality of residuals** - The within-subject residuals (deviations from each mouse's group by day mean are approximately normally distributed)
- **Sphericity (Homogeneity of covariances)** - The variances of the pairwise differences between levels of the within subject factor (`day`) are equal
- **Homoscedasticity of between-subjects factor** - The variance of observations across the levels of the between-subjects factor (`group`) is approximately equal
- **Balanced Design** - The within-subject levels are categorical and each subject has the same measurements at every level

1.7.1.1 Independence of Subjects

The subjects (`mice`) are independent of one another and had no effect on the other mice. All measurements came from the same mice at multiple time points

1.7.1.2 Normality of residuals

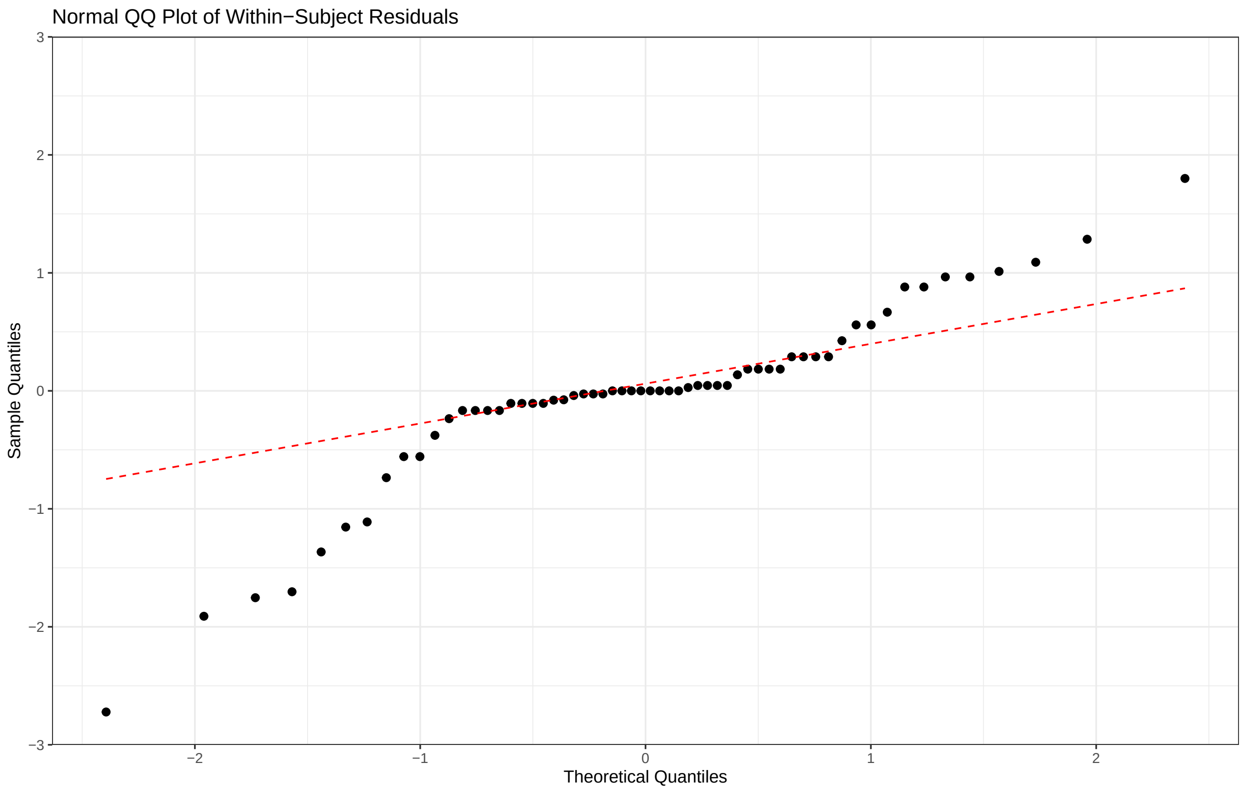

1.7.1.3 Sphericity

Mauchly's Test for Sphericity

	W	p-value	p<.05
day	0.961	0.622	
group:day	0.961	0.622	

1.7.1.4 Balanced Data The data contains no missing values for any of the trials, however there is an imbalance in the in the number of subjects for each of the antibiotic groups. Repeated-measures ANOVA typically require the same number of subjects when doing the comparisons, but the ANOVA is robust to moderate departures in it's assumptions.

1.7.2 Results

Repeated-Measures ANOVA Results

	DFn	DFd	SSn	SSd	F	p-value	p<.05	ges	
(Intercept)	1	25	476.99776	40.61745	293.59	0.000	*	0.8627490	*
group	4	25	22.36341	40.61745	3.44	0.023	*	0.2276245	*
day	2	50	12.36901	35.26607	8.77	0.001	*	0.1401547	*
group:day	8	50	15.62257	35.26607	2.77	0.013	*	0.1707271	*

Mauchly's Test for Sphericity

	W	p-value	p<.05
day	0.961	0.622	
group:day	0.961	0.622	

Dunnett Post-hoc Comparisons

Each antibiotic group vs. saline for VRE

	Estimate	SE	DF	Lower 95% CI	Upper 95% CI	t-ratio	p-value
NA							
1 Day - Saline	0.00	0.00	25	0.00	0.00	−2.15	0.131
3 Day - Saline	0.00	0.00	25	0.00	0.00	−2.15	0.131
6 Day - Saline	0.00	0.00	25	0.00	0.00	−2.36	0.088
10 Day - Saline	0.00	0.00	25	0.00	0.00	−2.36	0.088
1 Day - Saline	0.26	0.78	25	−1.80	2.32	0.33	0.974
3 Day - Saline	0.41	0.78	25	−1.65	2.46	0.52	0.925
6 Day - Saline	1.37	0.72	25	−0.51	3.24	1.91	0.205
10 Day - Saline	2.55	0.72	25	0.67	4.42	3.56	0.006
1 Day - Saline	0.00	0.87	25	−2.27	2.27	0.00	1.000
3 Day - Saline	0.00	0.87	25	−2.27	2.27	0.00	1.000
6 Day - Saline	0.00	0.79	25	−2.08	2.08	0.00	1.000
10 Day - Saline	1.31	0.79	25	−0.77	3.38	1.65	0.313

Overall Group Comparisons

Comparisons of overall average VRE CFU differences for antibiotics groups

	Estimate	SE	DF	Lower 95% CI	Upper 95% CI	t-ratio	p-value
Saline - 1 Day	−0.09	0.49	25	−1.61	1.43	−0.18	1.000
Saline - 3 Day	−0.14	0.49	25	−1.66	1.38	−0.28	1.000
Saline - 6 Day	−0.46	0.45	25	−1.84	0.93	−1.01	1.000
Saline - 10 Day	−1.28	0.45	25	−2.67	0.10	−2.85	0.085
1 Day - 3 Day	−0.05	0.47	25	−1.48	1.38	−0.11	1.000
1 Day - 6 Day	−0.37	0.42	25	−1.66	0.92	−0.88	1.000
1 Day - 10 Day	−1.20	0.42	25	−2.49	0.09	−2.85	0.085
3 Day - 6 Day	−0.32	0.42	25	−1.61	0.97	−0.76	1.000
3 Day - 10 Day	−1.15	0.42	25	−2.44	0.14	−2.74	0.090
6 Day - 10 Day	−0.83	0.37	25	−1.96	0.30	−2.25	0.233

1.8 Repeated Measure ANOVA (Klebsiella)

1.8.1 Model Diagnostics

The repeated measures ANOVA has the following assumptions:

- **Independence of subjects** - Each mouse's response is independent of every other's
- **Normality of residuals** - The within-subject residuals (deviations from each mouse's group by day mean are approximately normally distributed)
- **Sphericity (Homogeneity of covariances)** - The variances of the pairwise differences between levels of the within subject factor (`day`) are equal
- **Homoscedasticity of between-subjects factor** - The variance of observations across the levels of the between-subjects factor (`group`) is approximately equal
- **Balanced Design** - The within-subject levels are categorical and each subject has the same measurements at every level

1.8.1.1 Independence of Subjects

The subjects (`mice`) are independent of one another and had no effect on the other mice. All measurements came from the same mice at multiple time points

1.8.1.2 Normality of residuals

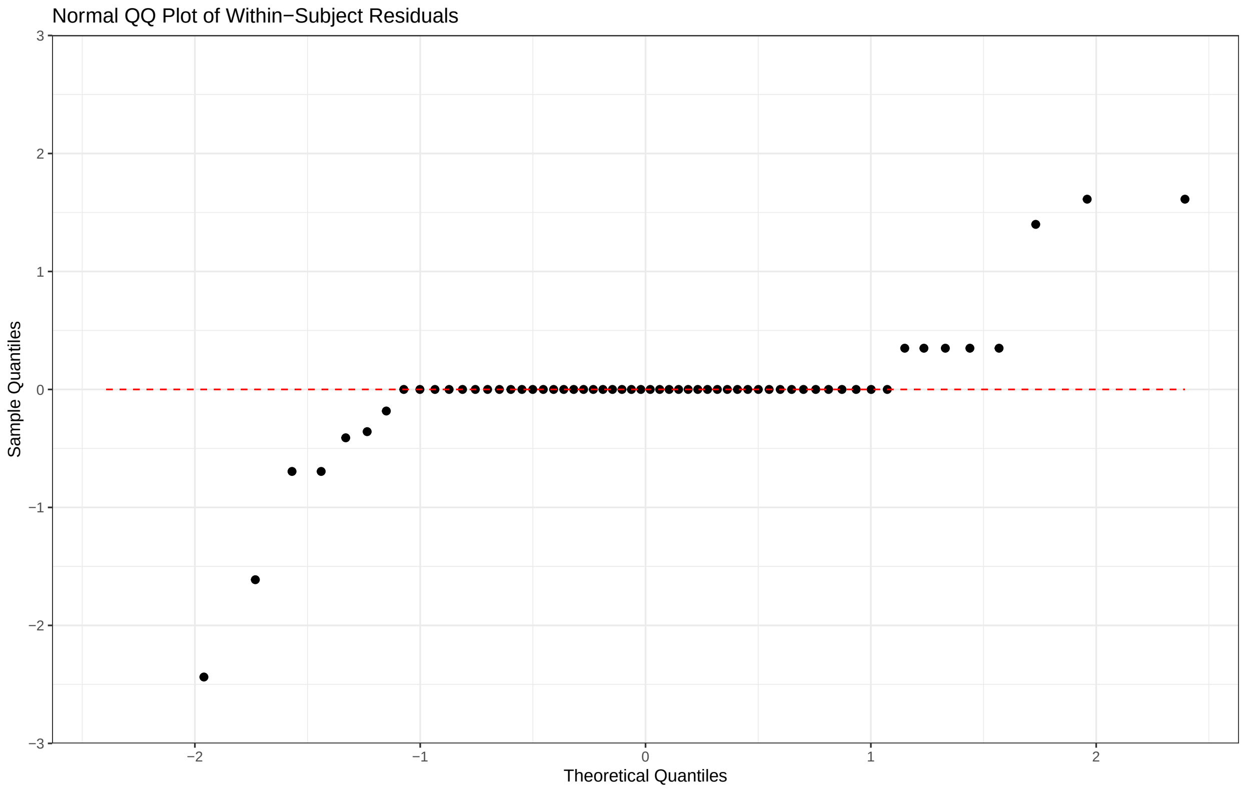

1.8.1.3 Sphericity

Mauchly's Test for Sphericity

	W	p-value	p<.05
day	0.222	0.000	*
group:day	0.222	0.000	*

```
## # A tibble: 2 x 6
##   Effect      df1_orig df2_orig epsilon_GG df1_adj df2_adj
##   <chr>          <dbl>    <dbl>      <dbl>   <dbl>   <dbl>
## 1 Day                2        8      0.222   0.444    1.78
## 2 Group x Day       50       50      0.222  11.1     11.1
```

Greenhouse–Geisser DF Adjustment

	df_1	df_2	ϵ_{GG}	$df_1 \times \epsilon_{GG}$	$df_2 \times \epsilon_{GG}$
Day	2	8	0.222	0.44	1.78
Group x Day	50	50	0.222	11.10	11.10

1.8.1.4 Balanced Data The data contains no missing values for any of the trials, however there is an imbalance in the in the number of subjects for each of the antibiotic groups. Repeated-measures ANOVA typically require the same number of subjects when doing the comparisons, but the ANOVA is robust to moderate departures in it's assumptions.

1.8.2 Results

Repeated-Measures ANOVA Results

	DFn	DFd	SSn	SSd	F	p-value	p<.05	ges	
(Intercept)	1	25	396.434294	17.75343	558.25	0.000	*	0.89674577	*
group	4	25	14.431295	17.75343	5.08	0.004	*	0.24020919	*
day	2	50	3.025458	27.89331	2.71	0.076		0.06215989	
group:day	8	50	15.974418	27.89331	3.58	0.002	*	0.25923595	*

Mauchly's Test for Sphericity

	W	p-value	p<.05
day	0.222	0.000	*
group:day	0.222	0.000	*

```
## # A tibble: 2 x 6
##   Effect      df1_orig df2_orig epsilon_GG df1_adj df2_adj
##   <chr>          <dbl>    <dbl>      <dbl>   <dbl>   <dbl>
## 1 Day                2        8      0.222   0.444    1.78
## 2 Group x Day       50       50      0.222  11.1     11.1
```

Greenhouse–Geisser DF Adjustment

	df_1	df_2	ϵ_{GG}	$df_1 \times \epsilon_{GG}$	$df_2 \times \epsilon_{GG}$
Day	2	8	0.222	0.44	1.78
Group x Day	50	50	0.222	11.10	11.10

Dunnett Post-hoc Comparisons

Each antibiotic group vs. saline for Klebsiella

	Estimate	SE	DF	Lower 95% CI	Upper 95% CI	t-ratio	p-value
NA							
1 Day - Saline	0.00	0.00	25	0.00	0.00	−2.15	0.131
3 Day - Saline	0.00	0.00	25	0.00	0.00	−2.15	0.131
6 Day - Saline	0.00	0.00	25	0.00	0.00	−2.36	0.088
10 Day - Saline	0.00	0.00	25	0.00	0.00	−2.36	0.088
1 Day - Saline	0.00	0.25	25	−0.65	0.65	0.00	1.000
3 Day - Saline	0.00	0.25	25	−0.65	0.65	0.00	1.000
6 Day - Saline	0.00	0.23	25	−0.59	0.59	0.00	1.000
10 Day - Saline	0.49	0.23	25	−0.10	1.09	2.19	0.123
1 Day - Saline	0.00	0.87	25	−2.29	2.29	0.00	1.000
3 Day - Saline	0.00	0.87	25	−2.29	2.29	0.00	1.000
6 Day - Saline	0.00	0.80	25	−2.09	2.09	0.00	1.000
10 Day - Saline	2.22	0.80	25	0.13	4.31	2.79	0.034

Overall Group Comparisons

Comparisons of overall average VRE CFU differences for antibiotics groups

	Estimate	SE	DF	Lower 95% CI	Upper 95% CI	t-ratio	p-value
Saline - 1 Day	0.00	0.33	25	−1.00	1.00	0.00	1.000
Saline - 3 Day	0.00	0.33	25	−1.00	1.00	0.00	1.000
Saline - 6 Day	0.00	0.30	25	−0.92	0.92	0.00	1.000
Saline - 10 Day	−0.91	0.30	25	−1.82	0.01	−3.04	0.038
1 Day - 3 Day	0.00	0.31	25	−0.95	0.95	0.00	1.000
1 Day - 6 Day	0.00	0.28	25	−0.85	0.85	0.00	1.000
1 Day - 10 Day	−0.91	0.28	25	−1.76	−0.05	−3.26	0.029
3 Day - 6 Day	0.00	0.28	25	−0.85	0.85	0.00	1.000
3 Day - 10 Day	−0.91	0.28	25	−1.76	−0.05	−3.26	0.029
6 Day - 10 Day	−0.91	0.24	25	−1.65	−0.16	−3.72	0.010

1.8.3 Conclusions
