# Supplementary material for: Longer durations of piperacillin/tazobactam treatment cause more prolonged alteration of colonization resistance in mice
Source: PLoS One. 2026 Jun 1;21(6):e0350031. doi: 10.1371/journal.pone.0350031 (PMC13225349; doi:10.1371/journal.pone.0350031)
Supplement: S4 Table — (PDF) [file pone.0350031.s004.pdf]

Antibiotic Duration for Gram Negatives and Enterococci Day 0

Samir Memic

2025-06-10

Contents

1 Antibiotic Duration Effects on Gram Negatives and Enterococci **2**
1.1 Summary . . . 2
1.1.1 Variable Summary for Gram Negatives and Enterococci Data . . . 2
1.2 Loading the Data . . . 2
1.3 Numerical Summary . . . 3
1.4 EDA . . . 6
1.5 Repeated Measures ANOVA . . . 6
1.5.1 Model Diagnostics . . . 6
1.5.2 Results . . . 9
1.5.3 Conclusions . . . 14
1.6 Linear Mixed Model . . . 14
1.6.1 Model Diagnositics . . . 14
1.6.2 Results . . . 20
1.6.3 Model Conclusions . . . 24
1.7 Repeated Measures ANOVA (Gram Negative) . . . 24
1.7.1 Model Diagnostics . . . 24
1.7.2 Results . . . 26
1.7.3 Conclusions . . . 28
1.8 Repeated Measures ANOVA (Enterococci) . . . 28
1.8.1 Model Diagnostics . . . 28
1.8.2 Results . . . 30
1.8.3 Conclusions . . . 31
1.9 Linear Mixed Model (Gram Negatives) . . . 31
1.9.1 Model Diagnositics . . . 31
1.9.2 Results . . . 37
1.9.3 Conclusions . . . 40
1.10 Linear Mixed Model (Enterococci) . . . 40
1.10.1 Model Diagnositics . . . 40
1.10.2 Results . . . 46
1.10.3 Conclusions . . . 48

---

1 Antibiotic Duration Effects on Gram Negatives and Enterococci

1.1 Summary

1.1.1 Variable Summary for Gram Negatives and Enterococci Data

1.2 Loading the Data

Preview of the dataset:

Preview of Gram Negative and Enterocci Data

group	mice	day	organism	cfu
Saline	1	-1	Gram Negatives	7.079181
Saline	2	-1	Gram Negatives	7.079181
Saline	3	-1	Gram Negatives	7.301030
Saline	4	-1	Gram Negatives	5.778151
1 Day	5	-1	Gram Negatives	4.778151
1 Day	6	-1	Gram Negatives	6.000000
1 Day	7	-1	Gram Negatives	6.778151
1 Day	8	-1	Gram Negatives	5.602060
1 Day	9	-1	Gram Negatives	3.845098
3 Day	10	-1	Gram Negatives	6.146128

Missingness check:

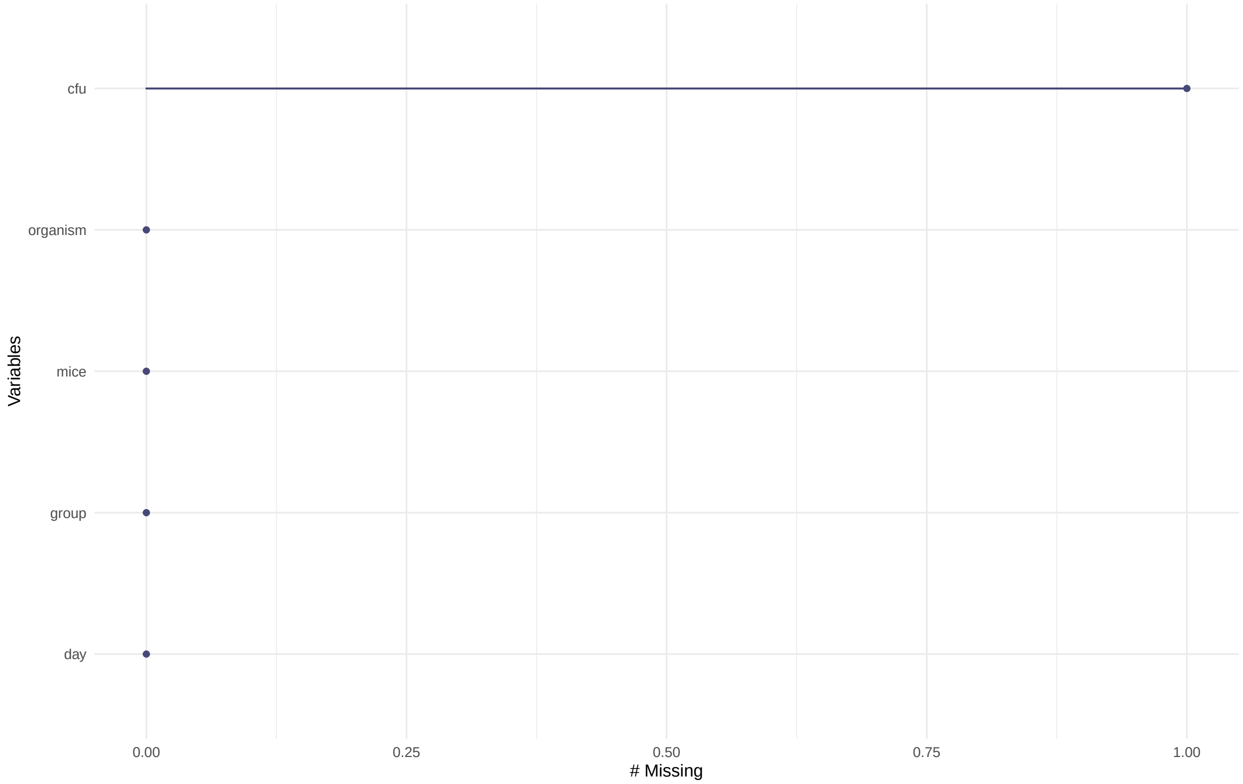

There is one mice that is missing it's CFU value. This has the potential to impact any repeated measures ANOVA.

1.3 Numerical Summary

```
## fig6
##
##  5  Variables      420  Observations
## ---------------------------------------------------------------------------
## group
##        n  missing distinct
##      420        0        5
##
## Value      Saline  1 Day  3 Day  6 Day 10 Day
## Frequency      56     70     70    112    112
## Proportion  0.133  0.167  0.167  0.267  0.267
## ---------------------------------------------------------------------------
## mice
##        n  missing distinct
##      420        0       30
##
## lowest : 1  2  3  4  5 , highest: 26 27 28 29 30
## ---------------------------------------------------------------------------
## day
##        n  missing distinct
##      420        0        7
##
## Value         -1     0     1     3     7    14    22
## Frequency     60    60    60    60    60    60    60
## Proportion 0.143 0.143 0.143 0.143 0.143 0.143 0.143
## ---------------------------------------------------------------------------
## organism
##        n  missing distinct
##      420        0        2
##
## Value      Gram Negatives    Enterococci
## Frequency             210            210
## Proportion            0.5            0.5
## ---------------------------------------------------------------------------
## cfu
##        n  missing distinct     Info     Mean      Gmd      .05      .10
##      419        1       88    0.918    4.778    3.035    2.000    2.000
##      .25      .50      .75      .90      .95
##    2.000    5.000    7.161    8.602    9.067
##
## lowest : 1       2       3       3.47712 3.60206
## highest: 10      10.2304 10.301  10.4771 10.6021
## ---------------------------------------------------------------------------
```

Gram Negatives Log_{10} CFU Recovered by Antibiotic Group and Day

day	n	Mean	SD	SEM	Median	IQR	Min	Max
Saline								
-1	4	6.81	0.70	0.35	7.08	0.38	5.78	7.30
0	4	6.39	0.26	0.13	6.39	0.29	6.08	6.70
1	4	6.18	0.30	0.15	6.24	0.27	5.78	6.48
3	4	6.33	0.45	0.23	6.45	0.48	5.70	6.70

7	4	6.78	0.13	0.07	6.81	0.13	6.60	6.90
14	4	6.31	0.56	0.28	6.07	0.41	5.95	7.15
22	4	6.15	0.72	0.36	5.85	0.60	5.70	7.20
1 Day								
-1	5	5.40	1.13	0.51	5.60	1.22	3.85	6.78
0	5	2.20	0.45	0.20	2.00	0.00	2.00	3.00
1	5	2.00	0.00	0.00	2.00	0.00	2.00	2.00
3	5	2.00	0.00	0.00	2.00	0.00	2.00	2.00
7	5	3.57	3.51	1.57	2.00	0.00	2.00	9.85
14	5	3.84	2.75	1.23	2.00	3.00	2.00	8.18
22	5	2.74	1.65	0.74	2.00	0.00	2.00	5.70
3 Day								
-1	5	5.86	0.36	0.16	5.95	0.67	5.48	6.23
0	5	2.00	0.00	0.00	2.00	0.00	2.00	2.00
1	5	2.00	0.00	0.00	2.00	0.00	2.00	2.00
3	5	2.00	0.00	0.00	2.00	0.00	2.00	2.00
7	5	3.94	3.30	1.47	2.00	2.08	2.00	9.61
14	5	5.77	3.57	1.60	6.78	6.78	2.00	9.30
22	5	4.78	2.62	1.17	5.60	5.00	2.00	7.30
6 Day								
-1	8	5.49	0.53	0.19	5.48	0.74	4.60	6.15
0	8	3.12	1.24	0.44	2.80	2.28	2.00	4.78
1	8	2.00	0.00	0.00	2.00	0.00	2.00	2.00
3	8	2.00	0.00	0.00	2.00	0.00	2.00	2.00
7	8	3.29	2.92	1.03	2.00	0.51	2.00	10.30
14	8	5.67	3.24	1.14	6.63	5.96	2.00	9.48
22	8	4.72	2.39	0.84	5.60	4.08	2.00	8.08
10 Day								
-1	8	6.20	0.53	0.19	6.36	0.77	5.30	6.78
0	8	3.34	1.14	0.40	3.80	2.24	2.00	4.60
1	8	2.00	0.00	0.00	2.00	0.00	2.00	2.00
3	8	2.12	0.35	0.12	2.00	0.00	2.00	3.00
7	8	2.59	1.66	0.59	2.00	0.00	2.00	6.70
14	8	2.63	1.44	0.51	2.00	0.25	2.00	6.08
22	8	2.00	0.00	0.00	2.00	0.00	2.00	2.00

Enterococci Log_{10} CFU Recovered by Antibiotic Group and Day

day	n	Mean	SD	SEM	Median	IQR	Min	Max
Saline								
-1	4	7.77	0.68	0.34	7.99	0.51	6.78	8.30
0	4	7.55	0.87	0.44	7.80	0.65	6.30	8.30
1	4	6.62	0.74	0.37	6.50	1.12	6.00	7.48
3	4	7.02	1.01	0.50	7.24	1.24	5.70	7.90
7	4	7.57	0.82	0.41	7.87	0.50	6.37	8.18
14	4	5.63	0.93	0.47	5.87	0.68	4.30	6.48
22	4	7.18	1.67	0.84	7.39	1.14	4.95	9.00
1 Day								

-1	5	7.03	0.84	0.37	6.78	1.00	5.95	8.04
0	5	2.00	0.00	0.00	2.00	0.00	2.00	2.00
1	5	6.71	3.00	1.34	7.40	2.59	2.00	9.78
3	5	6.19	3.84	1.72	8.48	7.00	2.00	9.48
7	5	5.87	2.44	1.09	6.90	2.48	2.00	7.95
14	5	6.01	2.38	1.07	6.95	1.90	2.00	7.78
22	5	5.44	3.19	1.43	7.00	5.60	2.00	8.60
3 Day								
-1	5	5.74	1.40	0.63	5.85	1.18	3.48	7.00
0	5	2.00	0.00	0.00	2.00	0.00	2.00	2.00
1	5	4.43	3.33	1.49	2.00	5.85	2.00	8.30
3	5	5.90	3.57	1.60	8.00	6.48	2.00	9.00
7	5	4.76	3.83	1.71	2.00	6.00	2.00	9.78
14	5	7.99	0.48	0.21	8.00	0.70	7.48	8.48
22	5	6.69	1.53	0.68	6.60	1.18	4.48	8.60
6 Day								
-1	8	6.05	0.94	0.33	5.94	0.94	4.85	7.78
0	8	2.00	0.00	0.00	2.00	0.00	2.00	2.00
1	8	3.64	2.79	0.99	2.00	2.08	2.00	8.85
3	8	4.24	3.68	1.30	2.00	6.40	1.00	9.30
7	8	6.21	3.53	1.25	8.10	6.93	2.00	9.60
14	8	6.34	2.95	1.04	7.54	3.78	2.00	9.70
22	8	5.64	2.57	0.91	6.00	2.62	2.00	9.00
10 Day								
-1	8	6.95	0.72	0.26	6.94	1.15	5.95	7.78
0	8	2.00	0.00	0.00	2.00	0.00	2.00	2.00
1	8	4.57	3.23	1.14	3.39	4.08	2.00	9.48
3	8	7.48	3.75	1.32	9.45	5.59	2.00	10.60
7	8	7.39	3.35	1.19	8.85	2.49	2.00	9.90
14	8	8.33	0.62	0.22	8.54	0.77	7.15	9.00
22	8	8.04	0.80	0.28	8.08	0.93	6.95	9.48

1.4 EDA

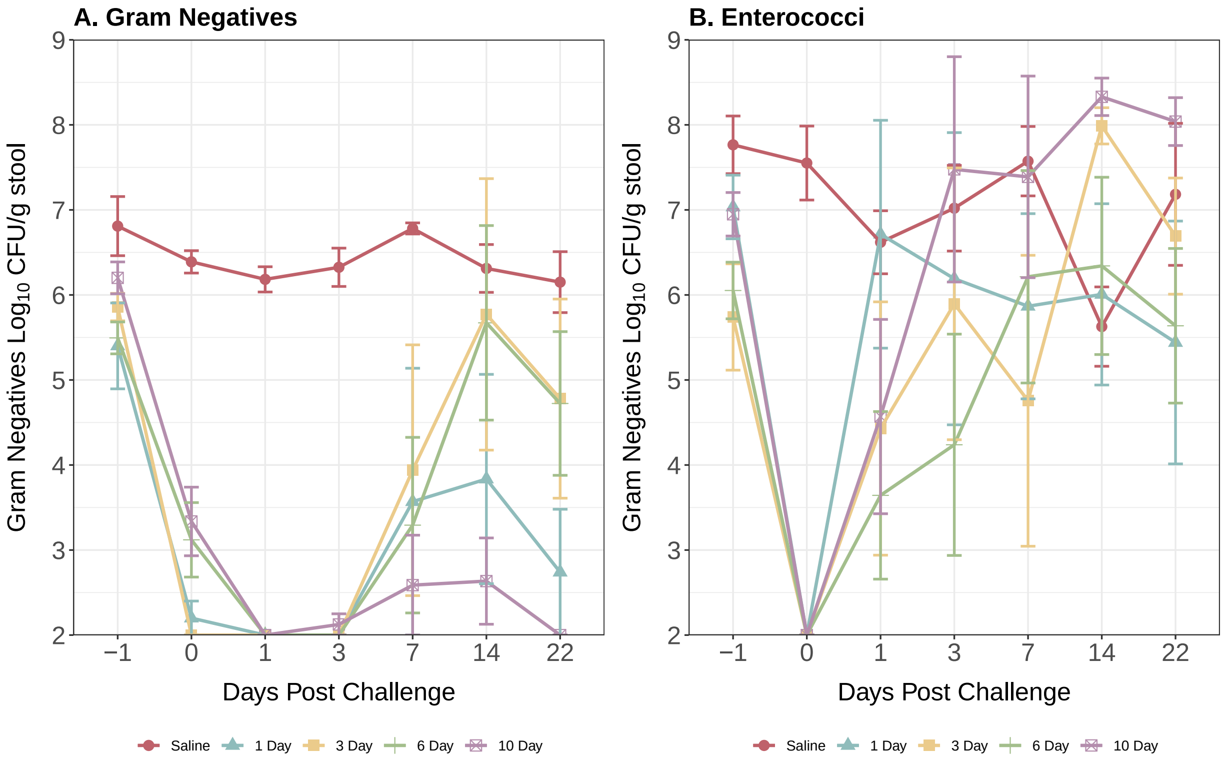

1.5 Repeated Measures ANOVA

A repeated measures ANOVA is an extension of the classic ANOVA in which each subject provides measurements at multiple levels of a within-subject factor. In this analysis, we assess how $\log_{10}$ CFU varies between antibiotic groups across by various days, and by organisms.

Of note, in a repeated measures ANOVA, if a subject is missing a value for any of its repeated measurements, the entire subject's data must be excluded for this analysis. In this case, the subject is `mice` 10 on day 14. To account for this, the mice will be dropped from the analysis. Therefore, the final dataset will contain 29 subjects after day -1. The

1.5.1 Model Diagnostics

The repeated measures ANOVA has the following assumptions:

- **Independence of subjects** - Each mouse's response is independent of every other's
- **Normality of residuals** - The within-subject residuals (deviations from each mouse's group by day mean are approximately normally distributed)
- **Sphericity (Homogeneity of covariances)** - The variances of the pairwise differences between levels of the within subject factor (`day`) are equal
- **Homoscedasticity of between-subjects factor** - The variance of observations across the levels of the between-subjects factor (`group`) is approximately equal
- **Balanced Design** - The within-subject levels are categorical and each subject has the same measurements at every level

1.5.1.1 Normality of Residuals

Normality of residuals in a repeated measure ANOVA is important because the model assumes the data is

distributed around a central value which is important for valid F-tests. Deviation from normality may lead to misleading results such as inflated p-values.

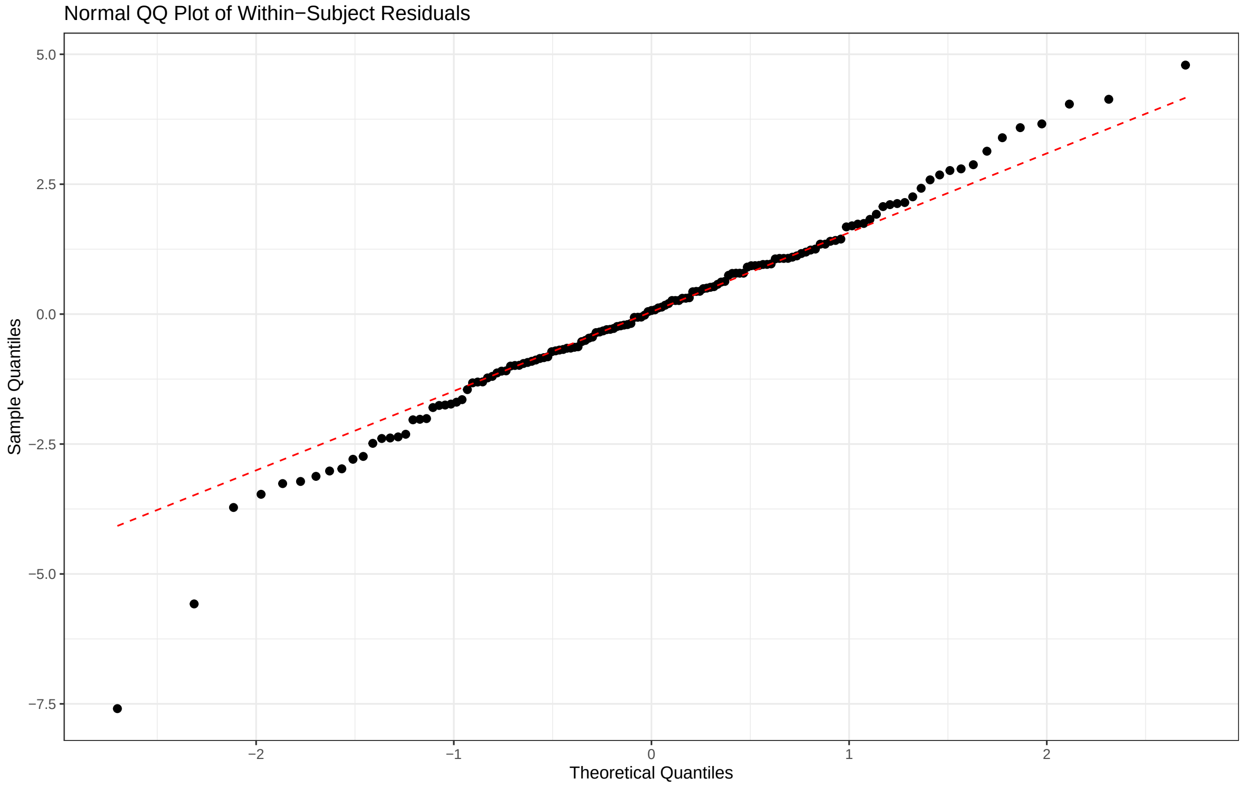

The QQ plot displays a normal QQ plot. The vast majority of points hug the red dashed line, indicating that most of the residuals conform to what would be expected within a normal distribution. There a few points that deviate from the distribution, but these make up a small proportion of the moderately sized dataset.

1.5.1.2 Sphericity

The sphericity assumption of the repeated measures ANOVA, is the assumption that the variances of the differences between all possible pairs of levels of the repeated measure factor (`day`) are equal. Violations of this assumption can lead to results being inflated and the higher probability of a type 1 error (False positive). Or in other words, it may lead to the repeated measures ANOVA may come to the false conclusion that there is a significant effect when there isn't one. A common test for sphericity is Mauchly's test. A significant p-value from a Mauchly's test suggests that sphericity has been violated:

Mauchly's Test for Sphericity

	W	p-value	p<.05
day	0.197	0.001	*
group:day	0.197	0.001	*
day:organism	0.173	0.000	*
group:day:organism	0.173	0.000	*

The table displays the results of the Mauchly's test. It corresponds to testing four effects: - `day` (The main within-subjects effect) - `group:day` (The group x day interaction) - `day:organism` (The day x organism interaction) - `group:day:organism` (The group x day x organism interaction)

The columns are as follows:

- **W** - Mauchly's W statistic (range 0-1; smaller values indicate greater departure from spherecitiy)
- **p** - The significance test for equality of variances of all pairwise differences
- **p<.05** - An asterisk (*) if p < .05, indicating a significant violation

For both the `day` and `group:day` effects had a $W = 0.197$ with $p < 0.001$. Because $p < 0.05$ in these cases, we reject the null hypothesis of sphericity. In other words, the variances of the differences between days are not all equal, and likewise for the interaction contrasts. To obtain valid F-tests for these within-subject effects, a correction—typically **Greenhouse–Geisser** or **Huynh–Feldt** - must be applied to adjust the degrees of freedom.

When Mauchly's test shows that sphericity is violated, the uncorrected F-tests for within-subjects effects will have inflated Type I error. Greenhouse–Geisser (GG) and Huynh–Feldt (HF) corrections adjust the degrees of freedom of those F-tests so that the p-values remain valid despite unequal variances of the contrasts. Slight differences exist between the two corrections:

- **Greenhouse–Geisser**
  - Provides a **conservative** estimate of how much to shrink both the numerator and denominator dfs.
  - Defined on [1/(k–1), 1], where k is the number of within-subject levels. An ϵ_{GG} closer to 0 indicates a **severe violation** of sphericity; values near 1 mean sphericity roughly holds.
- **Huynh–Feldt**
  - A **less conservative** alternative to GG, typically producing larger ϵ_{GG} (and thus larger effective dfs).
  - Also bounded by the same theoretical limits but often nearer to 1 than ϵ_{GG}.

Sphericity Corrections

	ϵ_{GG}	p[GG]	p[GG]<.05	ϵ_{HF}	p[HF]	p[HF]<.05
day	0.684	0.000	*	0.811	0.000	*
group:day	0.684	0.024	*	0.811	0.016	*
day:organism	0.635	0.002	*	0.743	0.001	*
group:day:organism	0.635	0.033	*	0.743	0.024	*

Both the **day** main effect and the **group×day** interaction have a correction factor of $\epsilon_{GG} = 0.684$ and $\epsilon_{HF} = 0.811$. After applying this correction factor to the degree of freedom, both the adjusted F-test remain highly significant ($p < 0.001$), so we retain the same conclusions about *day* and the *group* x *day* interaction on colonization levels, however, with the caveat that they are now properly controlled for Type 1 errors.

1.5.1.3 Homoscedasticity of between-subjects factor

To check for homoscedasticity, a Levene's test will be used. Levene's test is a formal way to check the homogeneity of variances (homoscedasticity) across two or more groups. For each observation it computes the absolute deviation from its group's center (mean or median), then runs a one-way ANOVA on those deviations. If the resulting F-test is significant, this indicates at least one group's variance is different from the other and that the equal-variance assumption is violated.

Levene's Test for Homogeneity of Variance (center = median)

Df	F value	Pr(>F)
4	3.338876	0.01061129
343		

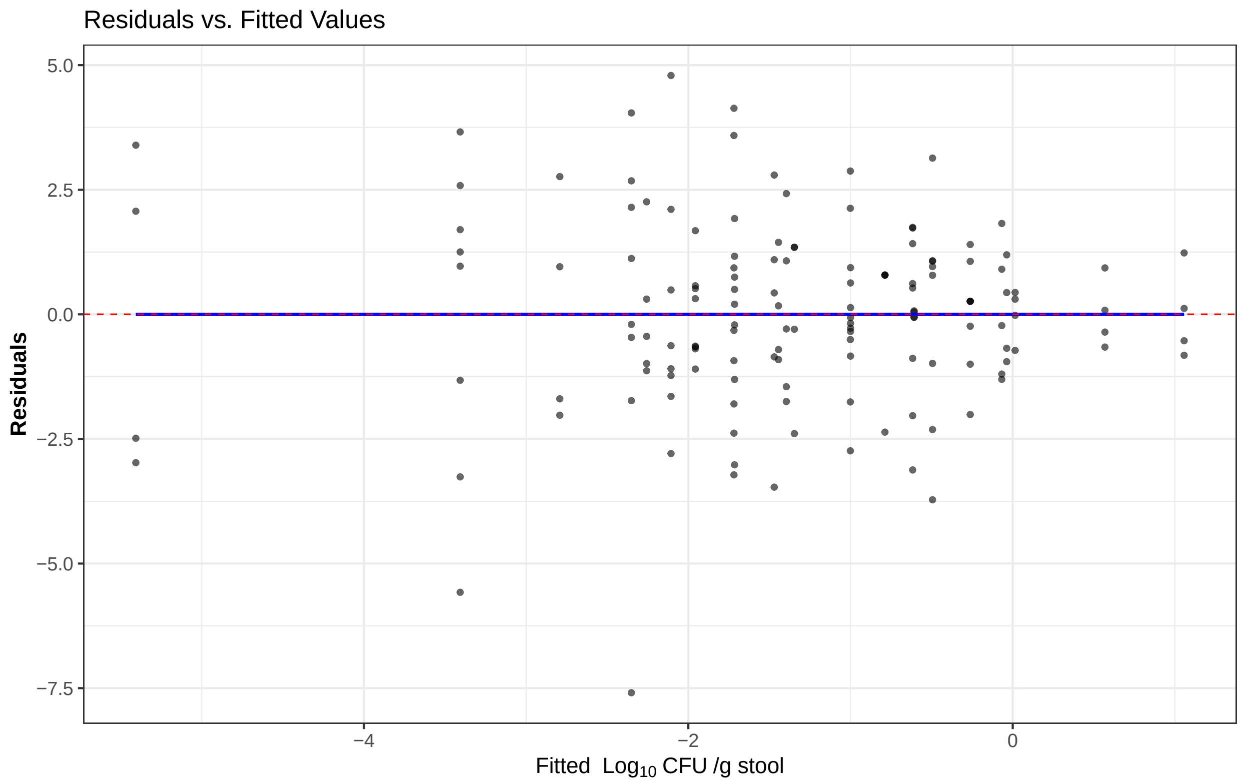

1.5.1.4 Balanced data

There is one `mice` that has a measurement missing, and has been excluded from the analysis.

1.5.2 Results

Repeated-Measures ANOVA Results

	DFn	DFd	SSn	SSd	F	p-value	p<.05	ges	
(Intercept)	1	24	6934.20968	256.1588	649.68	0.000	*	0.83999078	*
group	4	24	253.33877	256.1588	5.93	0.002	*	0.16092857	*
day	5	120	261.17203	489.3815	12.81	0.000	*	0.16508305	*
organism	1	24	274.86204	190.2474	34.67	0.000	*	0.17224582	*
group:day	20	120	167.91875	489.3815	2.06	0.009	*	0.11278714	*
group:organism	4	24	138.48439	190.2474	4.37	0.009	*	0.09489282	*
day:organism	5	120	87.40994	385.1047	5.45	0.000	*	0.06206760	*
group:day:organism	20	120	128.58717	385.1047	2.00	0.011	*	0.08871264	*

Mauchly's Test for Sphericity

	W	p-value	p<.05
day	0.197	0.001	*
group:day	0.197	0.001	*
day:organism	0.173	0.000	*
group:day:organism	0.173	0.000	*

Sphericity Corrections

	ϵ_{GG}	p[GG]	p[GG]<.05	ϵ_{HF}	p[HF]	p[HF]<.05
day	0.684	0.000	*	0.811	0.000	*
group:day	0.684	0.024	*	0.811	0.016	*
day:organism	0.635	0.002	*	0.743	0.001	*
group:day:organism	0.635	0.033	*	0.743	0.024	*

Greenhouse–Geisser DF Adjustment

	df_1	df_2	ϵ_{GG}	$df_1 \times \epsilon_{GG}$	$df_2 \times \epsilon_{GG}$
Day	5	120	0.684	3.42	82.08
Group x Day	20	120	0.684	13.68	82.08
Day x Organism	5	120	0.635	3.17	76.20
Group x Day x Organism	20	120	0.635	12.70	76.20

A repeated measures ANOVA was employed to examine the effects antibiotic duration (`group`), time (`day`), and bacterial organism type (Gram negatives and *Enterococci*) on the $\log_{10}$ CFU burden recovered from the stool of mice across six different time points. Mauchly's test indicated significant violations of the assumption of sphericity for all within-subject effects involving `day` (all $p < 0.05$). Thus, Greenhouse-Geiseer (GG) correction were applied ($\epsilon = 0.684$ and $\epsilon = 0.635$) to these terms to adjust degrees of freedom and significance levels.

The corrected analysis revealed significant main effects of `group` ($F(4,24) = 5.93$, $p = 0.002$), day ($F(3.42, 82.08) = 12.81$, $p < 0.001$, GG-corrected), and organism ($F(1,24) = 34.67$, $p < 0.001$). These results indicate that bacterial concentrations differentiated significantly by antibiotic treatment duration, the number of days after stopping antibiotics, and differed significantly between organisms commonly found in stool. Furthermore, significant interactions effects emerged for `group` x `organism` ($F(4,24) = 4.37$, $p = 0.009$), `day` x `organism` ($F(3.17, 76.20) = 5.45$, $p = 0.002$, GG corrected), and the three-way interaction term `group` x `day` x `organism` ($F(12.71, 76.20) = 2.00$, $p = 0.033$, GG corrected). These interactions highlight that the trajectory of microbial recovery following antibiotic administration is influenced not only by the type of organisms but also by the duration of antibiotic treatment and how these factors interact across multiple time points.

Dunnett Post-hoc Comparisons for Gram Negatives

Each antibiotic group vs. saline at each post-treatment day

	Estimate	SE	DF	Lower 95% CI	Upper 95% CI	t-ratio	p-value
Baseline							
1 Day - Saline	−4.19	1.44	233	−7.81	−0.57	−2.92	0.016
3 Day - Saline	−4.39	1.51	233	−8.20	−0.58	−2.90	0.016
6 Day - Saline	−3.27	1.31	233	−6.57	0.03	−2.49	0.027
10 Day - Saline	−3.05	1.31	233	−6.35	0.25	−2.33	0.027
1 Day Post-Treatment							
1 Day - Saline	−4.18	1.44	233	−7.80	−0.57	−2.91	0.008
3 Day - Saline	−4.18	1.51	233	−8.00	−0.37	−2.76	0.008
6 Day - Saline	−4.18	1.31	233	−7.48	−0.88	−3.19	0.006
10 Day - Saline	−4.18	1.31	233	−7.48	−0.88	−3.19	0.006
3 Day Post-Treatment							
1 Day - Saline	−4.33	1.44	233	−7.94	−0.71	−3.01	0.006

	Estimate	SE	DF	Lower 95% CI	Upper 95% CI	t-ratio	p-value
3 Day - Saline	−4.33	1.51	233	−8.14	−0.51	−2.86	0.006
6 Day - Saline	−4.33	1.31	233	−7.63	−1.02	−3.30	0.005
10 Day - Saline	−4.20	1.31	233	−7.50	−0.90	−3.20	0.005
7 Day Post-Treatment							
1 Day - Saline	−3.21	1.44	233	−6.83	0.40	−2.24	0.053
3 Day - Saline	−2.36	1.51	233	−6.17	1.45	−1.56	0.121
6 Day - Saline	−3.49	1.31	233	−6.79	−0.19	−2.66	0.025
10 Day - Saline	−4.19	1.31	233	−7.50	−0.89	−3.20	0.006
14 Day Post-Treatment							
1 Day - Saline	−2.48	1.44	233	−6.09	1.14	−1.72	0.258
3 Day - Saline	0.40	1.51	233	−3.41	4.21	0.27	1.000
6 Day - Saline	−0.64	1.31	233	−3.94	2.66	−0.49	1.000
10 Day - Saline	−3.68	1.31	233	−6.98	−0.38	−2.80	0.022
22 Day Post-Treatment							
1 Day - Saline	−3.41	1.44	233	−7.03	0.21	−2.37	0.055
3 Day - Saline	−0.67	1.51	233	−4.49	3.14	−0.45	0.656
6 Day - Saline	−1.43	1.31	233	−4.73	1.87	−1.09	0.555
10 Day - Saline	−4.15	1.31	233	−7.45	−0.85	−3.16	0.007

Dunnett Post-hoc Comparisons for Enterococci

Each antibiotic group vs. saline at each post-treatment day

	Estimate	SE	DF	Lower 95% CI	Upper 95% CI	t-ratio	p-value
Baseline							
1 Day - Saline	−5.55	1.44	233	−9.17	−1.93	−3.86	0.000
3 Day - Saline	−5.55	1.51	233	−9.36	−1.74	−3.67	0.000
6 Day - Saline	−5.55	1.31	233	−8.85	−2.25	−4.23	0.000
10 Day - Saline	−5.55	1.31	233	−8.85	−2.25	−4.23	0.000
1 Day Post-Treatment							
1 Day - Saline	0.09	1.44	233	−3.52	3.71	0.07	0.947
3 Day - Saline	−3.04	1.51	233	−6.86	0.77	−2.01	0.137
6 Day - Saline	−2.98	1.31	233	−6.28	0.33	−2.27	0.097
10 Day - Saline	−2.05	1.31	233	−5.35	1.25	−1.56	0.239
3 Day Post-Treatment							
1 Day - Saline	−0.83	1.44	233	−4.45	2.79	−0.58	1.000
3 Day - Saline	−1.90	1.51	233	−5.71	1.91	−1.26	0.632
6 Day - Saline	−2.78	1.31	233	−6.08	0.52	−2.12	0.140
10 Day - Saline	0.46	1.31	233	−2.84	3.76	0.35	1.000
7 Day Post-Treatment							
1 Day - Saline	−1.71	1.44	233	−5.32	1.91	−1.19	0.709
3 Day - Saline	−4.07	1.51	233	−7.88	−0.26	−2.69	0.031
6 Day - Saline	−1.36	1.31	233	−4.66	1.94	−1.04	0.709
10 Day - Saline	−0.18	1.31	233	−3.49	3.12	−0.14	0.888
14 Day Post-Treatment							
1 Day - Saline	0.38	1.44	233	−3.24	4.00	0.26	1.000
3 Day - Saline	2.36	1.51	233	−1.45	6.17	1.56	0.361

6 Day - Saline	0.71	1.31	233	−2.59	4.02	0.54	1.000
10 Day - Saline	2.70	1.31	233	−0.60	6.00	2.06	0.162
22 Day Post-Treatment							
1 Day - Saline	−1.74	1.44	233	−5.36	1.87	−1.21	0.906
3 Day - Saline	−0.47	1.51	233	−4.28	3.34	−0.31	1.000
6 Day - Saline	−1.55	1.31	233	−4.85	1.76	−1.18	0.906
10 Day - Saline	0.86	1.31	233	−2.45	4.16	0.65	1.000

Post-hoc contrasts were conducted using Dunnett's test and correction to compare each antibiotic group to the saline group across each of the time points for both gram negatives and *enterococci*.

For gram negatives, all antibiotic groups showed significantly lower bacterial counts compared to the saline control at baseline (Day 0, all $p < 0.05$). This reduction remained significant for all groups 1 and 3 days post-treatment (all $p < 0.05$). By 7 days post treatment, the mice treated with antibiotics for 1 and 3 days had lowered but non-significant bacterial burdens compared to the control group (δ = -3.21, 95% CI [-6.83, 0.40], $p = 0.053$ and δ = -2.36, 95% CI [-6.17, 1.45], $p = 0.121$, respectively), while the mice treated with 6 and 10 days of antibiotics remained significantly different from the controls ($p = 0.025$ and $p = 0.006$, respectively). Fourteen days after treatment, only the 10-day antibiotic mice remained significantly different from the controls (δ = -3.68, 95% CI [-6.98, -0.38], $p = 0.022$), while all other groups were non-significant (all $p > 0.05$). This trend continued into day 22, where only the 10 day antibiotic treatment mice had a lower microbial burden of gram negatives compared to the control group (δ = -4.15, 95% CI [-7.45, -0.85], $p = 0.007$), while all other antibiotic groups were lower but non-significantly different from the saline control.

For *enterococci* all antibiotic treatment groups significantly differed from saline group while on antibiotics compared to the saline group ($p < 0.001$). After arresting antibiotic treatment duration, the mice with a one-day treatment returned to similar levels compared to the saline group (δ = 0.09, 95% CI [-3.52, 3.71], p-value = 0.947). The 3, 6, and10 day antibiotic duration groups had lower but non-significant microbial burdens compared to the saline group (3 day: δ = -3.04, 95% CI [-6.86, 0.77], p-value = 0.137, 6 day: δ = -2.98, 95% CI [-6.28, 0.33], p-value = 0.097, 10 day: δ = -2.05, 95% CI [-5.35, 1.25], p-value = 0.239). This trend continued onto day 3 with all antibiotic groups remaining non-significant.

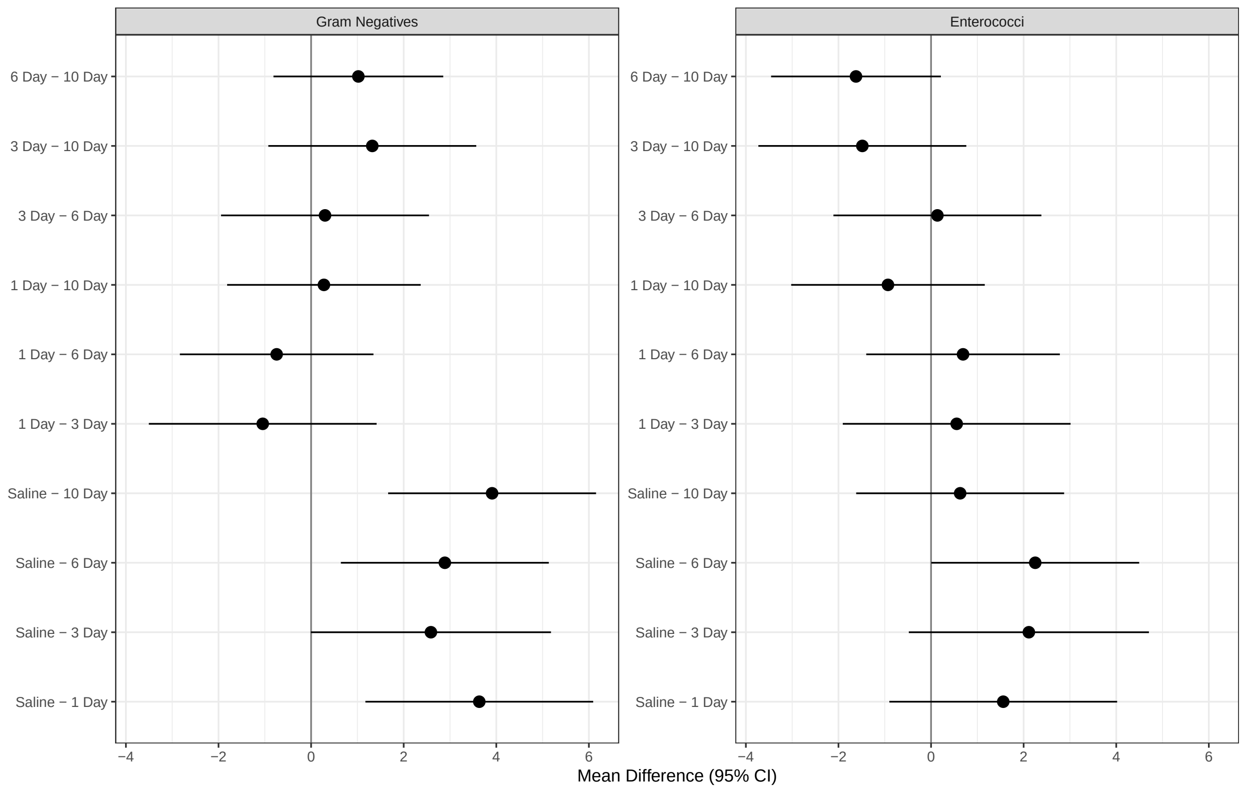
Gram Negatives
Enterococci
6 Day − 10 Day
3 Day − 10 Day
3 Day − 6 Day
1 Day − 10 Day
1 Day − 6 Day
1 Day − 3 Day
Saline − 10 Day
Saline − 6 Day
Saline − 3 Day
Saline − 1 Day
−4 −2 0 2 4 6
Mean Difference (95% CI)

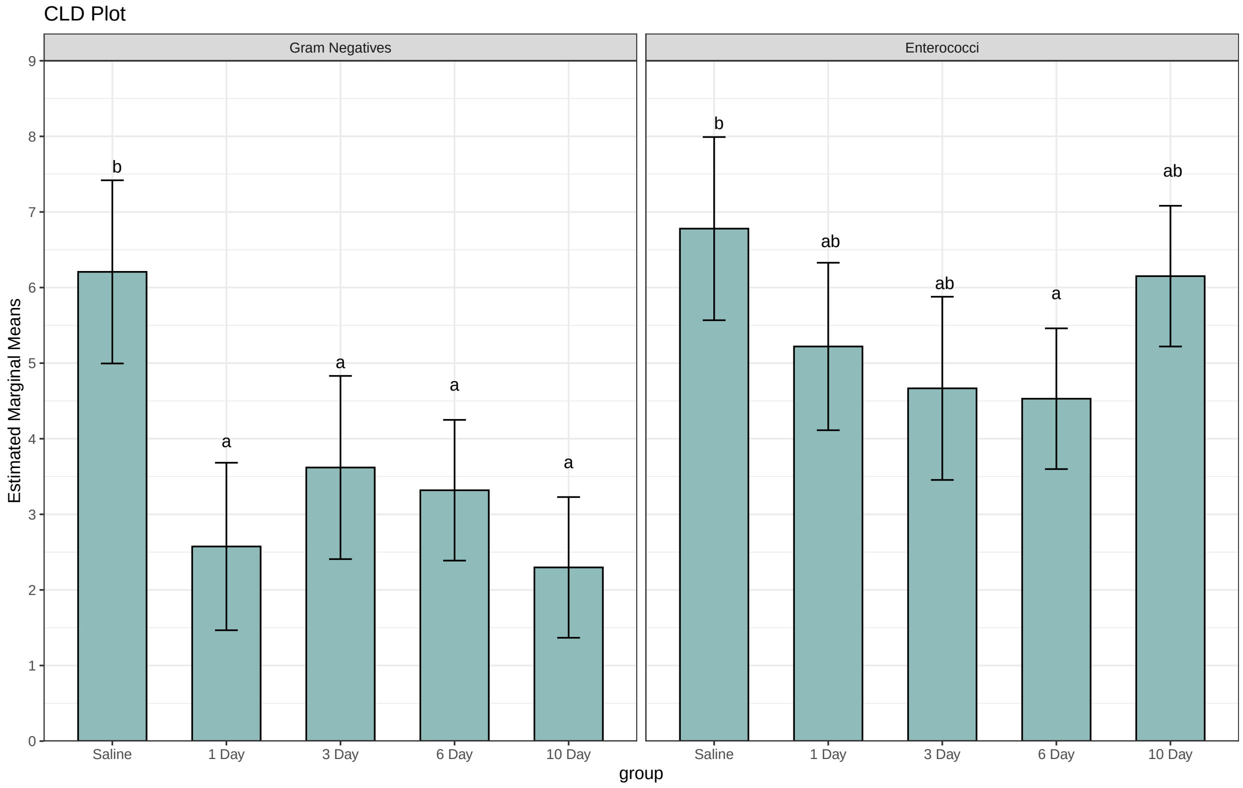
CLD Plot
Gram Negatives
Enterococci
Estimated Marginal Means
0 1 2 3 4 5 6 7 8 9
b
a
a
a
a
b
ab
ab
a
ab
Saline
1 Day
3 Day
6 Day
10 Day
group

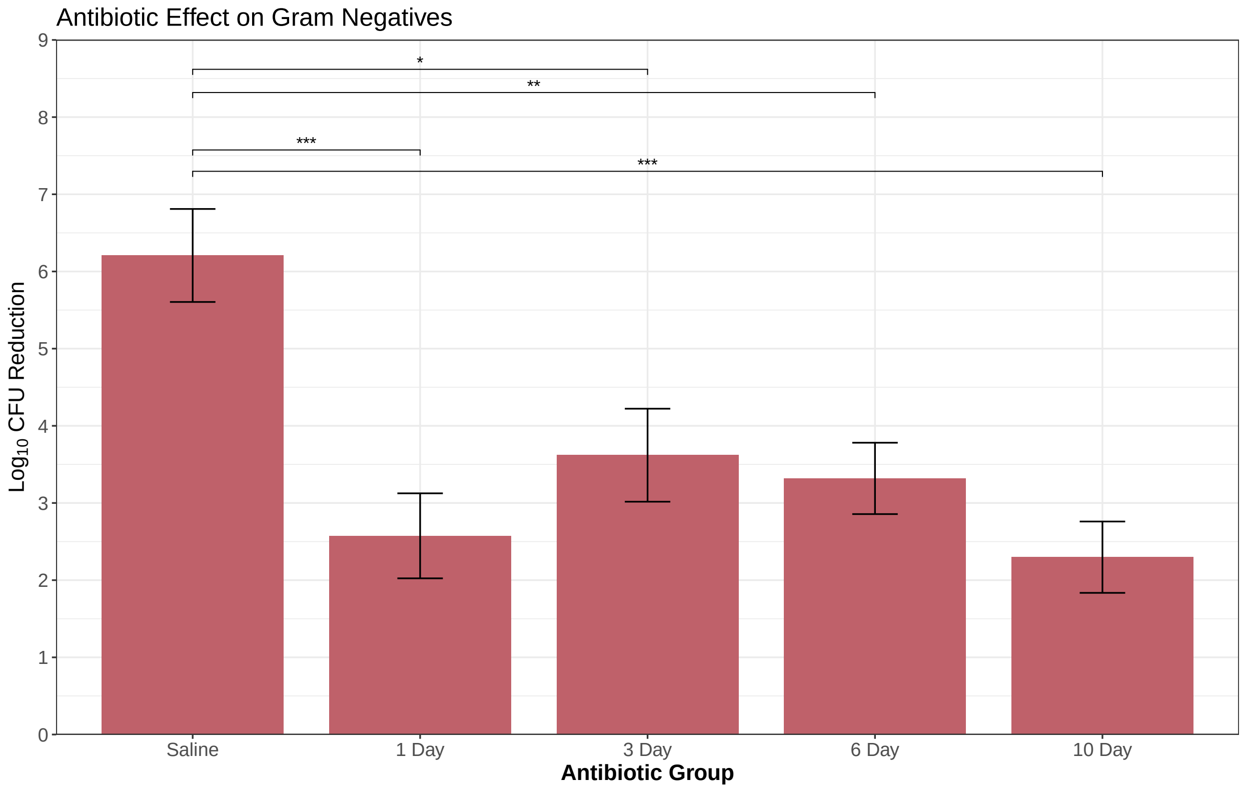

1.5.3 Conclusions

1.6 Linear Mixed Model

A linear mixed effects model is an extension of the ordinary regression model that incorporates fixed effects (population parameters such as antibiotic group, day, and their interaction) and random effects (Subject specific deviations that account for correlation among repeated measures, such as a random intercept for each mouse to account for baseline CFU levels). The model specified incorporated the fixed effects of group and day and their interaction in predicting $\log_{10}$ CFU levels. A random intercept was incorporated for each mice to allow them to have their own average baseline CFU levels. Furthermore, a variance identity structure was incorporated to allow each antibiotic among each organism to have it's own residual variance. This allows each antibiotic group for each organism to have it's own variance σ^2 rather than assuming all groups share the same variance. This is due to the repeated-measures ANOVA diagnostic plots indicating that variance differs between antibiotic groups and organisms. By properly accounting for this variance, the model's SE's for the fixed effects and their contrasts properly reflect the true hetroskedasticity ensuring that the confidence intervals and hypothesis tests are valid.

1.6.1 Model Diagnositics

Assumptions include:

- **Linearity** - The expected CFU change is a linear function of the fixed predictors. If this assumption is violated then β estimates will be biased.
- **Normality of Level-1 Residuals** - After accounting for fixed effects and random effects, the remaining residuals are approximately normal. Violations of this assumption causes p-values and confidence intervals for the fixed-effect to be biased.
- **Homoscedasticity of Residuals** - The variance of the residuals should be constant across levels of predictors. If residuals systematically change then standard error and test statistics can be wrong

- **Independence of Level-1 Errors** - Residuals (after accounting for random effects) are uncorrelated across observations. Violations inflate type 1 error.
- **Correct Random-Effects Structure** - Every random intercept slope should be included in the model. Violations of this assumption lead to biased standard errors and overfitting.
- **Normality of Random Effects** - The distribution of the random intercepts should be normal. Mixed Models assumes random effects come from a normal distribution, and severe departures can bias variance estimates
- **No Extreme Multicollinearity** - The fixed predictors are not exact linear combinations of one another. Violations lead to inflated standard errors and results in unstable coefficients.
- **Proper Nesting / Independence of Clusters** - The group factor (`mice`) should correctly partition the data into independent clusters, with no mouse appearing in two different clusters
- **No Missing Data** - Missing observations (if any) should be missing at random (MAR), so model-based likelihood inference remains valid.
- **No influential observations or outliers** - No single mouse or single observation should greatly impact the fixed-effect estimates

1.6.1.1 Linearity

Mixed effects models assume that the fixed-effect predictors capture the outcome variable in a linear fashion. Once we have accounted for the random effects of the data, the conditional mean of the outcome should approximately be a straight-line function for the covariates.

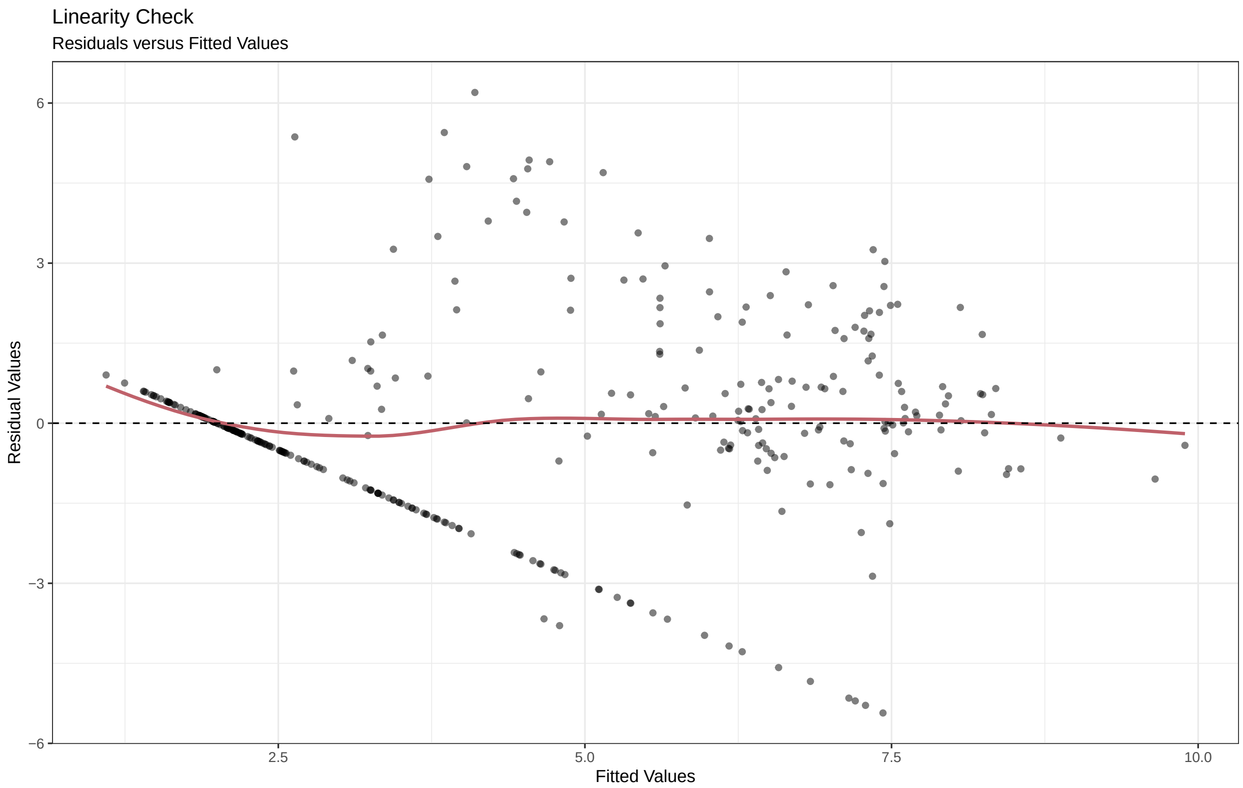

The red LOESS curve lies close to the dashed-zero-line across the entire range of values indicating that linearity does not change much. Furthermore, there is no U-shaped or inverted U-shaped patterns which would be indicative of under or over-fitting non-linear trends. The normality assumption is met.

1.6.1.2 Homoscedastictiy (Constant Variance of Residuals)

Homoscedasticity means that the residuals have constant variance across levels of fitted values or predictors.

The following will look to ensure that the spread of residuals remains roughly the same regardless of the predicted value.

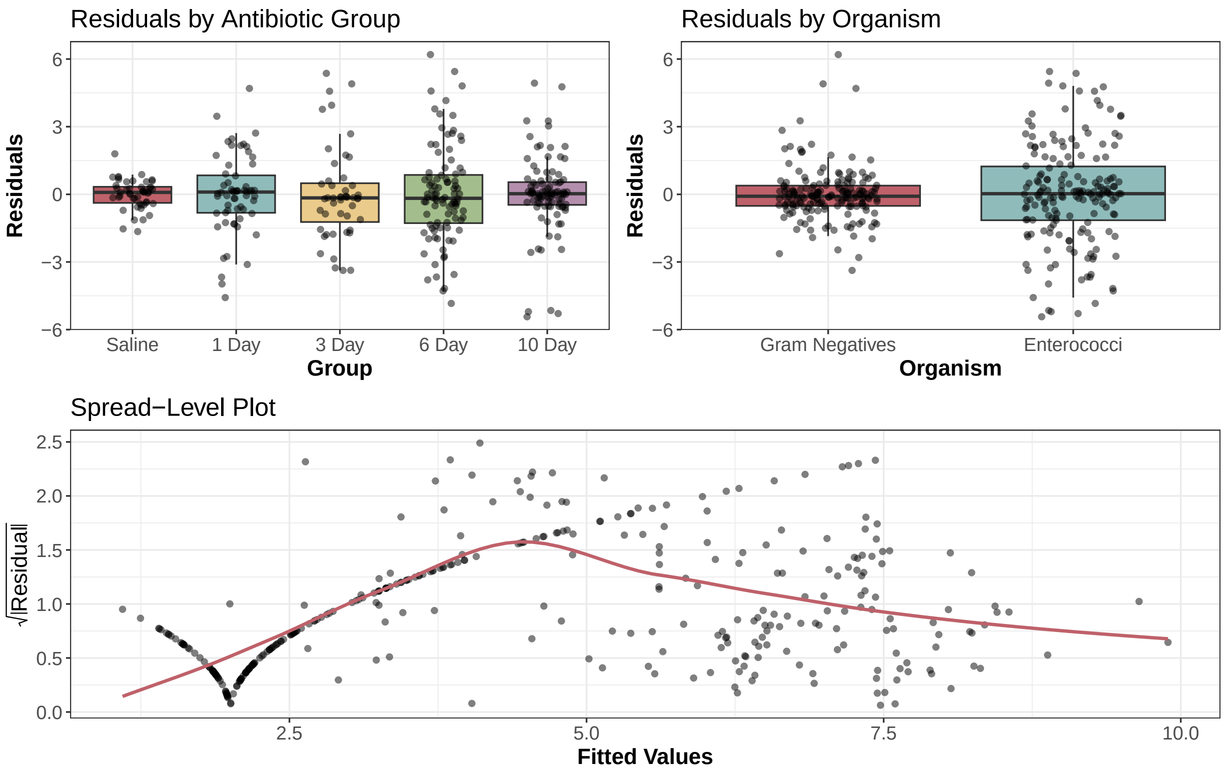

The residuals boxplots for both `organism` and `day` look fairly symmetric and centered around zero with no extreme outliers or major differences in spread across groups. The spread-level plot shows some curvature in the LOESS line, which suggest mild heteroscedasticity (i.e., non-consistent residual variance across fitted values). However, it's not too extreme so the model should be able to handle it relatively well since the model includes variance structures for `organisms` and `group`. Overall, the model passes the homoscedasticity check.

1.6.1.3 Normality of Level-1 Residuals The normality of residuals are needed to have valid hypothesis testing (i.e., p-values and confidence intervals). Deviation can occur with large samples, but major skewness or heavy tails may indicate problems with the model

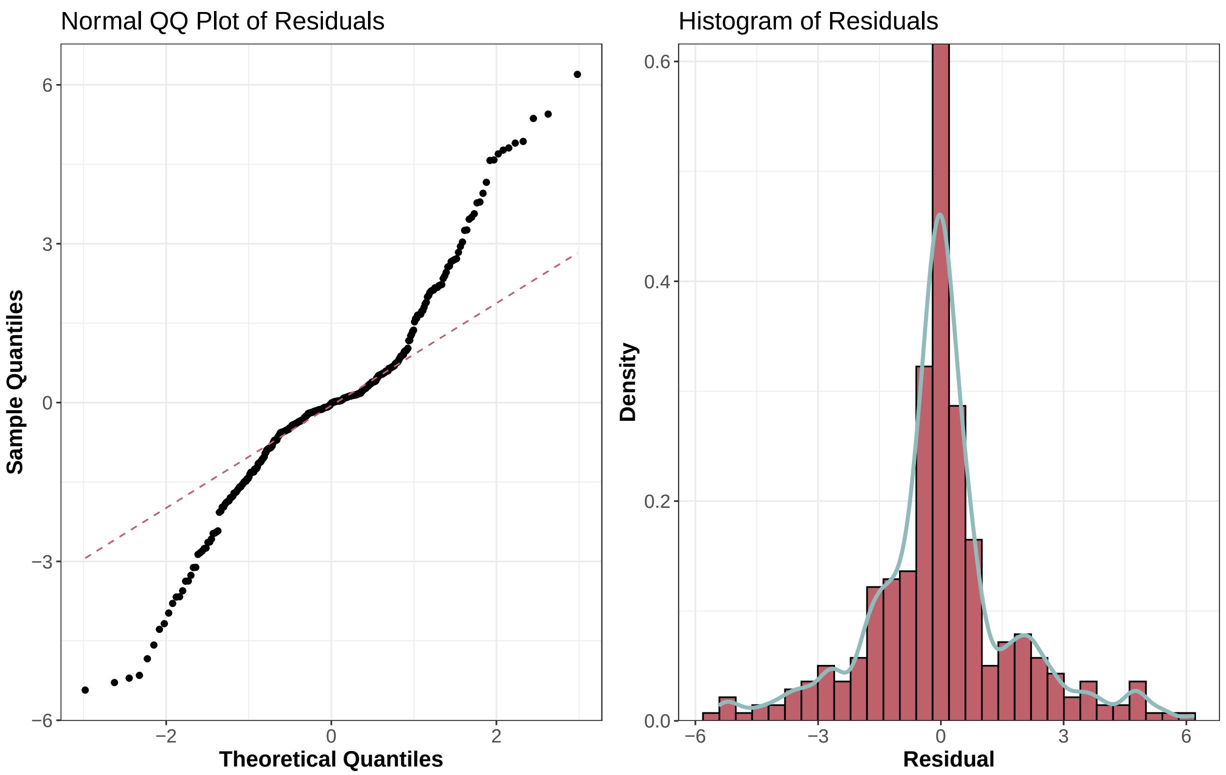

The QQ plot has an S-shaped curve and departs from normality at both tails indicating that the residuals deviate from a normal distribution. The Histogram confirms this trend. Although the residuals are centered at zero, the tails are heavier than expected under a normal curve. Additionally there appears to be several peaks within the histogram, potentially indicating different biological groups. This is somewhat expected when measuring various subjects under different biological conditions.

However, mixed models with many observations are fairly robust to moderate non-normality, and variance has been accounted for with the different variance structures specified within the model. For a conservative approach, we may consider robust standard error estimates and verifying the results using non-parametric methods or bootstrapping.

1.6.1.4 Independence of Residuals For a mixed effects model with repeated measures, the residuals should not be autocorrelated with one another (i.e., Residuals from one observation are not related to other across time within subjects).

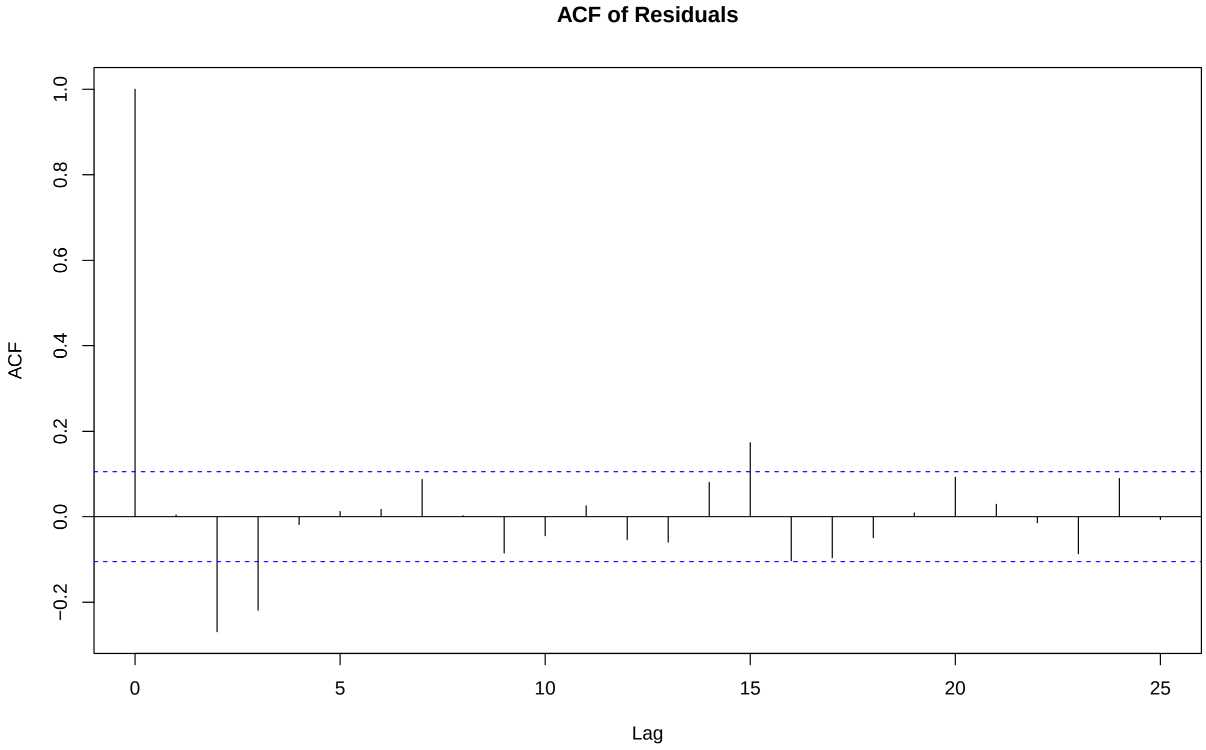


```
## Warning: Autocorrelated residuals detected (p = 0.006).
```

The model performance found that there is autocorrelation ($p = 0.004$). The ACF plot shows several spikes above the blue line, with most of them occurring at lag 0. This not a violation since this is expected for biological data, especially when the day 0 results are similar to one another. For the other spikes, since the vast majority are not above the blue line, this indicate the lags that are violated, are more likely due to random noise.

1.6.1.5 Normality of Random Effects Mixed effect models assume that the random effects (i.e., the random intercepts for `mice`) are normally distributed. Violations of this can impact the estimation of variance components and confidence intervals for fixed effects.

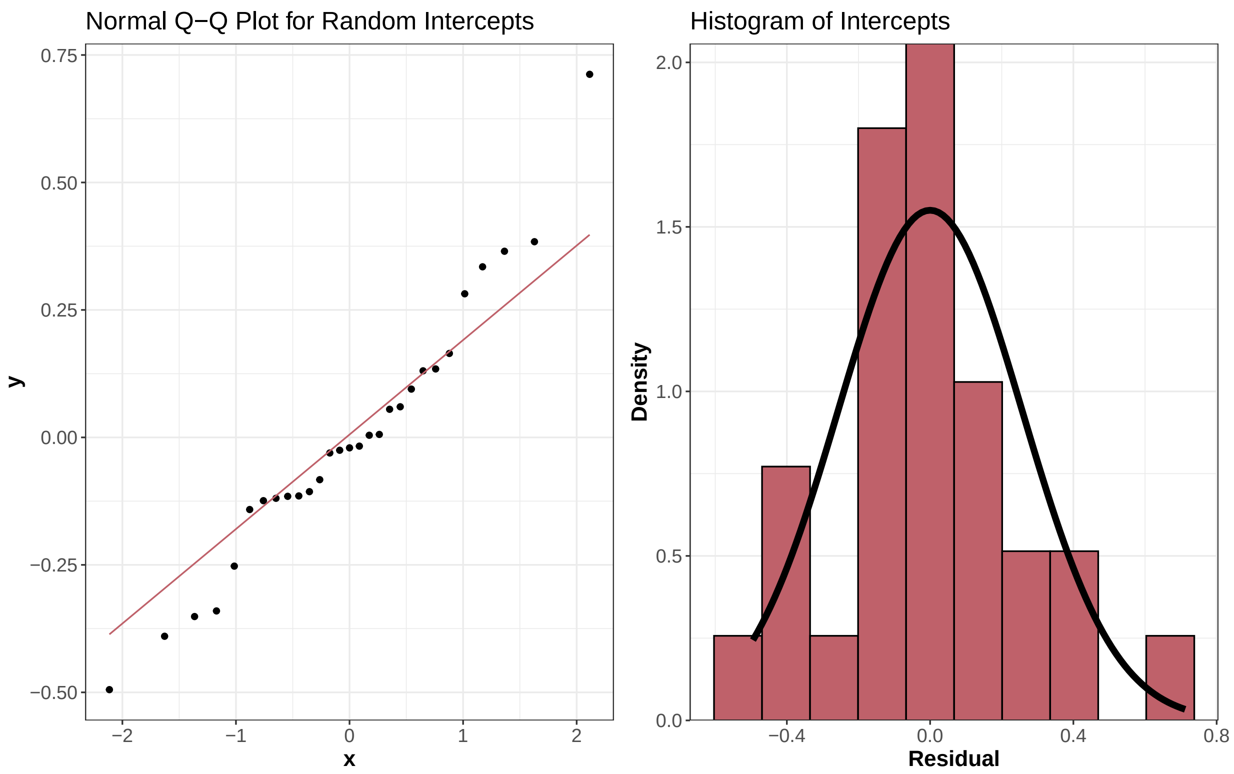

The QQ plot of the intercepts shows that most of the intercepts fall along the red line with only a few slight deviations from normality, and shows no major issues. The histogram plot of the intercepts further reaffirms this point with the intercepts following an approximately normal distribution Overall, there are no major issues with the normality of the random effects.

1.6.1.6 Multicollinearity

Multicollinearity between predictors can inflate standard errors, influence coefficients and cause instability in the model estimations.

term	GVIF	Df	GVIF^(1/(2*Df))
group	1.000000	4	1.000000
day	1.625204	5	1.049762
organism	1.102000	1	1.049762
group:day	1.625204	20	1.012215
group:organism	1.102000	4	1.012215
day:organism	1.625204	5	1.049762
group:day:organism	1.625204	20	1.012215

None of the interaction effects or the main effects have problematic GVIF values above 5 or 10, indicating there is no issues with multicollinearity within the model.

1.6.1.7 Properly Nested Data

Linear mixed effect models require that each cluster (i.e. `mice`) is independent of the other clusters and that observations within each cluster (i.e. repeated measure) are correctly grouped under the random-effects structure. Violations can lead to inflated degrees of freedom, biased variance estimates, and invalid standard errors.

All `mice` ID appear in exactly one group (no mouse switches between antibiotic conditions) and each mice

has the same number of observations (one per level of day per organism), so the repeated measures structure is balanced and nested. This indicates our data is properly nested for a random-intercept model.

1.6.1.8 Outliers If the model contains extreme observations this can influence the model leading to influencing fixed-effect estimates and bias model diagnostics.

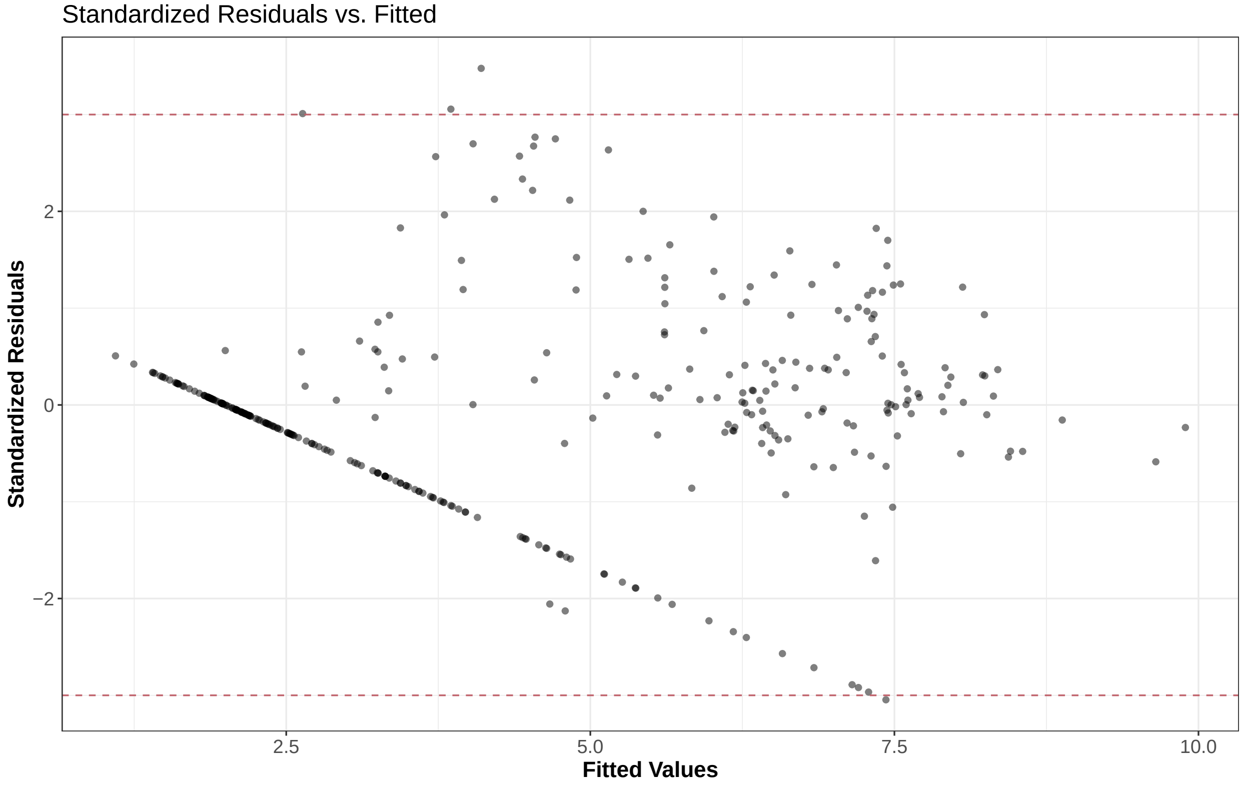

There are only a few points that have a standardized residual greater than -3 or 3, but even then this points do not lie far from the line. There is no issue with outliers within the data.

1.6.2 Results

Fixed Effects Estimates – Linear Mixed Model

Modeling CFU by group × day × organism

Term	Estimate	SE	Lower 95% CI	Upper 95% CI	z	p-value
(Intercept)	4.69	0.17	4.36	5.01	28.28	0.000
1 Day	1.96	0.32	1.33	2.58	6.13	0.000
3 Day	−0.64	0.34	−1.31	0.03	−1.87	0.061
6 Day	−0.39	0.40	−1.18	0.39	−0.99	0.325
10 Day	−0.61	0.31	−1.21	−0.01	−2.00	0.045
1	−1.43	0.24	−1.89	−0.96	−6.05	0.000
3	−0.76	0.23	−1.21	−0.30	−3.25	0.001
7	−0.24	0.23	−0.68	0.21	−1.04	0.299
14	0.43	0.22	0.00	0.87	1.94	0.052
22	1.26	0.23	0.81	1.71	5.46	0.000
Enterococci	−0.93	0.10	−1.13	−0.74	−9.36	0.000
1 Day × 1	1.75	0.32	1.12	2.39	5.43	0.000

3 Day × 1	−0.52	0.49	−1.48	0.44	−1.06	0.289
6 Day × 1	−0.87	0.63	−2.09	0.36	−1.38	0.167
10 Day × 1	−0.09	0.47	−1.02	0.84	−0.18	0.854
1 Day × 3	0.51	0.31	−0.10	1.13	1.65	0.100
3 Day × 3	1.07	0.49	0.11	2.02	2.20	0.028
6 Day × 3	−0.75	0.62	−1.97	0.47	−1.21	0.228
10 Day × 3	−0.50	0.47	−1.42	0.42	−1.06	0.290
1 Day × 7	0.27	0.29	−0.31	0.84	0.90	0.366
3 Day × 7	0.28	0.48	−0.65	1.22	0.60	0.549
6 Day × 7	−0.50	0.61	−1.70	0.70	−0.81	0.417
10 Day × 7	−0.72	0.46	−1.62	0.19	−1.55	0.120
1 Day × 14	0.10	0.28	−0.44	0.64	0.37	0.714
3 Day × 14	0.24	0.47	−0.68	1.15	0.51	0.611
6 Day × 14	−0.76	0.60	−1.95	0.42	−1.27	0.205
10 Day × 14	0.25	0.46	−0.65	1.14	0.54	0.589
1 Day × 22	−1.93	0.31	−2.53	−1.33	−6.33	0.000
3 Day × 22	−0.39	0.48	−1.33	0.56	−0.80	0.422
6 Day × 22	1.80	0.62	0.59	3.01	2.91	0.004
10 Day × 22	0.67	0.47	−0.24	1.59	1.44	0.149
1 Day × Enterococci	0.65	0.12	0.41	0.89	5.27	0.000
3 Day × Enterococci	−0.39	0.21	−0.80	0.02	−1.87	0.061
6 Day × Enterococci	0.41	0.27	−0.12	0.94	1.52	0.129
10 Day × Enterococci	0.33	0.20	−0.07	0.73	1.61	0.107
1 × Enterococci	1.08	0.22	0.65	1.52	4.86	0.000
3 × Enterococci	−0.16	0.22	−0.60	0.28	−0.72	0.471
7 × Enterococci	−0.63	0.22	−1.06	−0.19	−2.81	0.005
14 × Enterococci	−0.06	0.22	−0.49	0.38	−0.25	0.802
22 × Enterococci	0.02	0.22	−0.42	0.46	0.09	0.927
1 Day × 1 × Enterococci	−1.38	0.27	−1.92	−0.84	−5.01	0.000
3 Day × 1 × Enterococci	0.34	0.47	−0.57	1.25	0.73	0.465
6 Day × 1 × Enterococci	−0.56	0.60	−1.74	0.63	−0.92	0.355
10 Day × 1 × Enterococci	0.08	0.46	−0.81	0.98	0.18	0.856
1 Day × 3 × Enterococci	0.23	0.27	−0.31	0.77	0.83	0.405
3 Day × 3 × Enterococci	−0.87	0.47	−1.79	0.04	−1.87	0.061
6 Day × 3 × Enterococci	−0.10	0.60	−1.29	1.08	−0.17	0.864
10 Day × 3 × Enterococci	−0.06	0.46	−0.95	0.84	−0.12	0.904
1 Day × 7 × Enterococci	0.57	0.27	0.03	1.10	2.06	0.040
3 Day × 7 × Enterococci	−0.15	0.47	−1.06	0.77	−0.31	0.754
6 Day × 7 × Enterococci	−0.41	0.60	−1.59	0.77	−0.68	0.497
10 Day × 7 × Enterococci	0.11	0.46	−0.78	1.01	0.25	0.804
1 Day × 14 × Enterococci	−0.05	0.27	−0.59	0.48	−0.20	0.845
3 Day × 14 × Enterococci	0.23	0.47	−0.69	1.15	0.49	0.623
6 Day × 14 × Enterococci	1.04	0.60	−0.14	2.22	1.73	0.084
10 Day × 14 × Enterococci	−0.80	0.46	−1.69	0.09	−1.76	0.079
1 Day × 22 × Enterococci	0.61	0.27	0.07	1.15	2.21	0.027
3 Day × 22 × Enterococci	0.22	0.47	−0.70	1.13	0.47	0.641
6 Day × 22 × Enterococci	−0.13	0.60	−1.32	1.05	−0.22	0.825
10 Day × 22 × Enterococci	0.25	0.46	−0.64	1.14	0.55	0.583

Random Effects Variance Components

Variance and standard deviation for each random effect

Group	Effect	Variance	Std. Dev
mice	(Intercept)	0.092	0.303
Residual		0.242	0.492

Post-Hoc Contrasts (Gram Negatives)

Each antibiotic group compared to Saline

	Estimate	SE	DF	Lower 95% CI	Upper 95% CI	t-ratio	p-value
0 Day Post-Treatment							
1 Day - Saline	−4.19	0.60	279	−5.70	−2.68	−6.96	0.000
3 Day - Saline	−4.39	0.78	279	−6.36	−2.42	−5.60	0.000
6 Day - Saline	−3.27	0.58	279	−4.73	−1.80	−5.61	0.000
10 Day - Saline	−3.05	0.46	279	−4.21	−1.90	−6.66	0.000
1 Day Post-Treatment							
1 Day - Saline	−4.18	0.61	279	−5.73	−2.64	−6.82	0.000
3 Day - Saline	−4.18	0.79	279	−6.18	−2.19	−5.27	0.000
6 Day - Saline	−4.18	0.59	279	−5.67	−2.69	−7.05	0.000
10 Day - Saline	−4.18	0.47	279	−5.37	−3.00	−8.88	0.000
3 Day Post-Treatment							
1 Day - Saline	−4.33	0.64	279	−5.94	−2.71	−6.74	0.000
3 Day - Saline	−4.33	0.82	279	−6.38	−2.27	−5.29	0.000
6 Day - Saline	−4.33	0.62	279	−5.88	−2.77	−7.01	0.000
10 Day - Saline	−4.20	0.50	279	−5.46	−2.94	−8.39	0.000
7 Day Post-Treatment							
1 Day - Saline	−3.21	0.71	279	−5.00	−1.42	−4.51	0.000
3 Day - Saline	−2.36	0.88	279	−4.57	−0.14	−2.68	0.008
6 Day - Saline	−3.49	0.68	279	−5.20	−1.78	−5.14	0.000
10 Day - Saline	−4.19	0.58	279	−5.64	−2.75	−7.29	0.000
14 Day Post-Treatment							
1 Day - Saline	−2.48	0.87	279	−4.67	−0.28	−2.83	0.015
3 Day - Saline	0.40	1.03	279	−2.19	2.99	0.39	0.873
6 Day - Saline	−0.64	0.82	279	−2.70	1.42	−0.78	0.873
10 Day - Saline	−3.68	0.74	279	−5.53	−1.82	−4.98	0.000
22 Day Post-Treatment							
1 Day - Saline	−3.41	1.09	279	−6.16	−0.67	−3.12	0.006
3 Day - Saline	−0.67	1.24	279	−3.79	2.44	−0.54	0.587
6 Day - Saline	−1.43	1.02	279	−3.98	1.13	−1.41	0.322
10 Day - Saline	−4.15	0.95	279	−6.54	−1.76	−4.37	0.000

Post-hoc comparisons reveal a consistent and significant reduction in Gram-negative concentration in across all antibiotic treatment groups compared to the saline control on day 0, 1, 3, and 7. At baseline (Day 0), all antibiotic groups (1 Day, 3 Day, 6 day, and 10 day) exhibited substantial reduction in $\log_{10}$ CFU/g stool, with estimated differences ranging from -3.05 to -4.39 $\log_{10}$ CFU/g ($p < 0.001$). This suppressive effect remained through days 1 and 3, where all antibiotic treatment groups continued to differ significantly from saline, suggesting any duration of antibiotic treatment will decrease the concentration native gram negative bacteria located within the intestines.

By day 7, all treatment groups remained statistically significant different from the saline group, the magnitude of these differences began to shift back to baseline concentrations, particularly in the 1 day and 3 day antibiotic groups (δ = -3.21, CI [-4.51, -1.42], $p < 0.001$ and δ = -2.36, 95% CI [-4.57, -0.14], $p = 0.008$, respectively). By day 14, only the 1 day and 10 day antibiotic groups remained significantly different from the saline group ($p = 0.015$ and $p < 0.001$, respectively). Notably, the 3 day and 6 day antibiotic groups no longer differed significantly from the saline group by this day (δ = 0.40, 95% CI [0.39, 2.99], p = 0.873, and δ = -0.64, 95% CI [-0.78, 1.42], p = 0.873, respectively), suggesting that partial microbial regrowth or gram negatives at intermediate antibiotic treatment durations.

After 22 days, the 10 day antibiotic treatment group continued to exhibit significant reductions in Gram-negative stool concentrations (δ = -4.15, 95% CI [-6.54, -1.76], $p < 0.001$), and the 1 day antibiotic group was also significantly different from the saline group (δ = -3.41, 95% CI [-6.16, -0.67], $p = 0.006$). However, 3 and 6 day treatment durations with antibiotics remained statistically indistinguishable from the control (p = 0.587 and p = 0.322, respectively), further indicating that intermediate antibiotic durations have a minimal impact on naturally occurring gram-negative flora compared to shorter and longer antibiotic treatment durations.

Post-Hoc Contrasts (Enterococci)

Each antibiotic group compared to Saline

	Estimate	SE	DF	Lower 95% CI	Upper 95% CI	t-ratio	p-value
**0 Day Post-Treatment**							
1 Day - Saline	−5.55	1.12	279	−8.37	−2.73	−4.95	0.000
3 Day - Saline	−5.55	1.48	279	−9.27	−1.83	−3.75	0.000
6 Day - Saline	−5.55	1.09	279	−8.29	−2.81	−5.09	0.000
10 Day - Saline	−5.55	0.84	279	−7.66	−3.44	−6.62	0.000
**1 Day Post-Treatment**							
1 Day - Saline	0.09	1.13	279	−2.74	2.93	0.08	0.933
3 Day - Saline	−3.04	1.49	279	−6.78	0.69	−2.05	0.083
6 Day - Saline	−2.98	1.10	279	−5.73	−0.22	−2.71	0.028
10 Day - Saline	−2.05	0.85	279	−4.17	0.07	−2.43	0.048
**3 Day Post-Treatment**							
1 Day - Saline	−0.83	1.14	279	−3.70	2.04	−0.73	0.937
3 Day - Saline	−1.90	1.50	279	−5.67	1.87	−1.27	0.617
6 Day - Saline	−2.78	1.11	279	−5.57	0.01	−2.51	0.051
10 Day - Saline	0.46	0.86	279	−1.71	2.62	0.53	0.937
**7 Day Post-Treatment**							
1 Day - Saline	−1.71	1.19	279	−4.69	1.28	−1.44	0.456
3 Day - Saline	−4.07	1.53	279	−7.93	−0.22	−2.66	0.033
6 Day - Saline	−1.36	1.14	279	−4.23	1.52	−1.19	0.471
10 Day - Saline	−0.18	0.91	279	−2.47	2.10	−0.20	0.839
**14 Day Post-Treatment**							
1 Day - Saline	0.38	1.29	279	−2.86	3.62	0.29	1.000
3 Day - Saline	2.36	1.63	279	−1.73	6.45	1.45	0.443
6 Day - Saline	0.71	1.23	279	−2.39	3.81	0.58	1.000
10 Day - Saline	2.70	1.02	279	0.14	5.26	2.65	0.034
**22 Day Post-Treatment**							
1 Day - Saline	−1.74	1.44	279	−5.37	1.89	−1.21	0.915
3 Day - Saline	−0.47	1.76	279	−4.90	3.96	−0.27	0.938
6 Day - Saline	−1.55	1.37	279	−4.99	1.90	−1.13	0.915

10 Day - Saline	0.86	1.18	279	−2.11	3.82	0.72	0.938

Antibiotic treatment durations effects on *Enterococci* flora exhibited noteworthy different temporal patterns compared to gram-negatives, with fewer sustained differences between treatment groups and the saline control. At baseline (day 0), all antibiotic duration groups (1 day, 3 day, 6 day, and 10 day) showed large and statistically significant reductions in *Enterococci* flora compared to the saline group (all $p < 0.001$), suggesting that there is a uniform effect across all treatment durations while receiving antibiotics.

However, beginning at 1 day post-antibiotic treatment, a divergence in *Enterococci* recolonization emerges. The 1 day antibiotic group returned to baseline concentration levels and had no significant difference from the saline group (δ = 0.09, 95% CI [-2.74, 2.93], p = 0.937), and the 3 day group showed lower but non-significant differences (δ = -3.04, 95% CI [-6.78, 0.69], p = 0.083) in *Enterococci* burden. In contrast, the 6 day (δ = -2.98, 95% CI [-5.73, -0.22], p = 0.028) and 10 day (δ = -2.05, 95% CI [-4.17, 0.07], p = 0.048) remained significantly lower than the saline groups, with *Enterococci* colonization recovering for all groups compared to day on antibiotic treatment. This indicates that shorter courses of antibiotic have a smaller impact on *Enterococcal* levels compared to longer courses (6-10 days). At day 3, none of the antibiotic groups were significantly different from saline (all $p > 0.05$), with the day 6 antibiotic group having the largest difference compared to the saline group (δ = -2.78, 95% CI [-5.57, 0.01], p = 0.051).

At day 7, the 3 day antibiotic group was statistically significant compared to the saline group (p = 0.033), while the 1 day,6 day, and 10 day groups had no significant difference. Notably, this trend reversed by day 14, the 10 day group showed a significant increase in *Enterococci* concentration (δ = 2.70, 95% CI [2.65, 5.26], p = 0.0034), while all other groups remained non significant. By day 22, no significant differences between any antibiotic groups and the saline control remained (all $p > 0.05$), indicating microbial convergence back to baseline across all groups.

1.6.3 Model Conclusions

Our results demonstrate that antibiotic duration significantly influences the dynamics of naturally occurring microbes such as gram negatives organisms and *Enterococci.* For gram negatives, all antibiotic groups exhibited rapid and substantial reductions in colonization compared to saline controls, particularly while on antibiotics, and 1 and 3 days after stopping antibiotics. The greatest effects were observed in the the 10 day antibiotic treatment group, which significantly lowered the level of gram negatives up to 22 days after stopping antibiotics. In contrast, *Enterococcal* were less uniformly affected by antibiotic exposure. Although all treatment groups initially experienced reductions at baseline, only the the 6 day treatment group remained significantly different 1 day after antibiotic duration, and all antibiotic duration groups were non significant by day 3. By day 22, all antibiotic treatment groups remained comparable to the saline group.

1.7 Repeated Measures ANOVA (Gram Negative)

A repeated measures ANOVA is an extension of the classic ANOVA in which each subject provides measurements at multiple levels of a within-subject factor. In this analysis, we assess how $\log_{10}$ CFU varies between antibiotic groups across by various days for gram negatives.

1.7.1 Model Diagnostics

The repeated measures ANOVA has the following assumptions:

- **Independence of subjects** - Each mouse's response is independent of every other's
- **Normality of residuals** - The within-subject residuals (deviations from each mouse's group by day mean are approximately normally distributed)
- **Sphericity (Homogeneity of covariances)** - The variances of the pairwise differences between levels of the within subject factor (`day`) are equal
- **Homoscedasticity of between-subjects factor** - The variance of observations across the levels of the between-subjects factor (`group`) is approximately equal

- **Balanced Design** - The within-subject levels are categorical and each subject has the same measurements at every level

1.7.1.1 Normality of Residuals

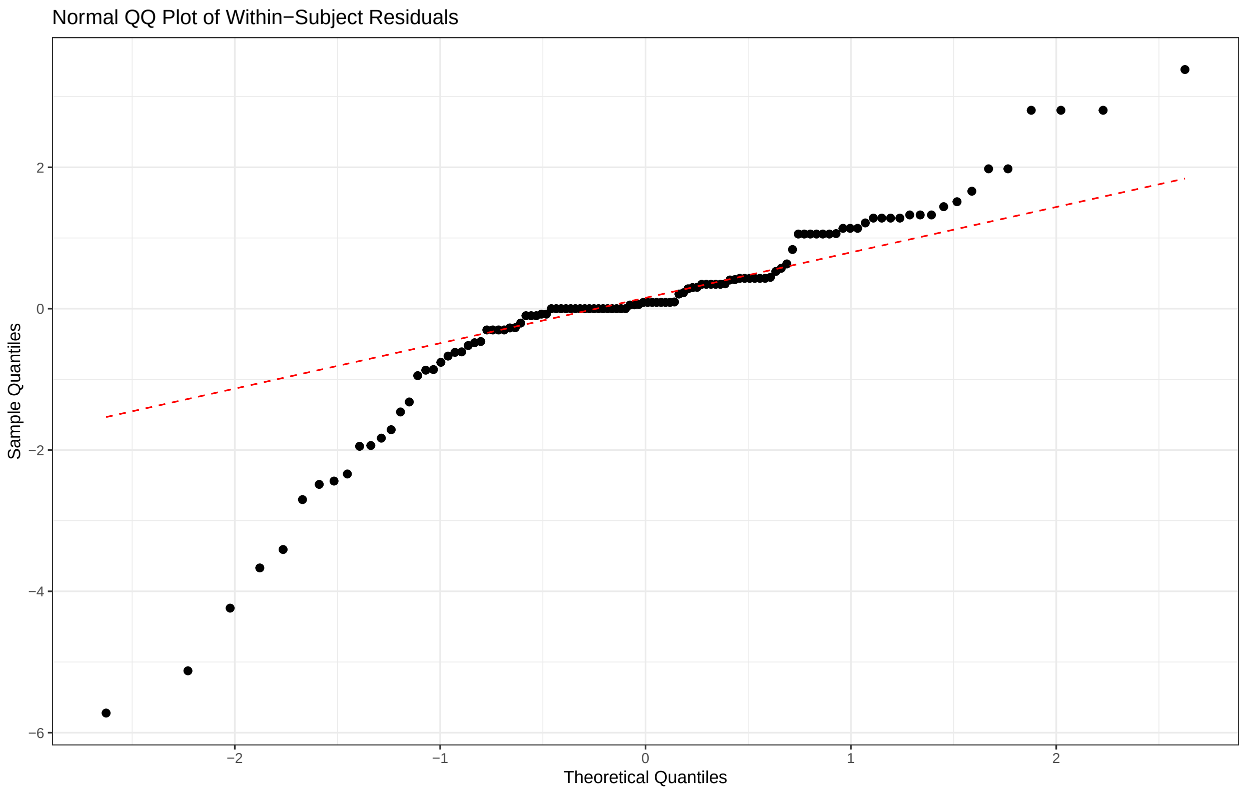

1.7.1.2 Sphericity

Mauchly's Test for Sphericity

	W	p-value	p<.05
day	0.004	0.000	*
group:day	0.004	0.000	*

Sphericity Corrections

	ϵ_{GG}	p[GG]	p[GG]<.05	ϵ_{HF}	p[HF]	p[HF]<.05
day	0.464	0.000	*	0.517	0.000	*
group:day	0.464	0.028	*	0.517	0.023	*

1.7.1.3 Homoscedasticity of between-subjects factor

Levene's Test for Homogeneity of Variance (center = median)

Df	F value	Pr(>F)
4	3.511961	0.008788221
169		

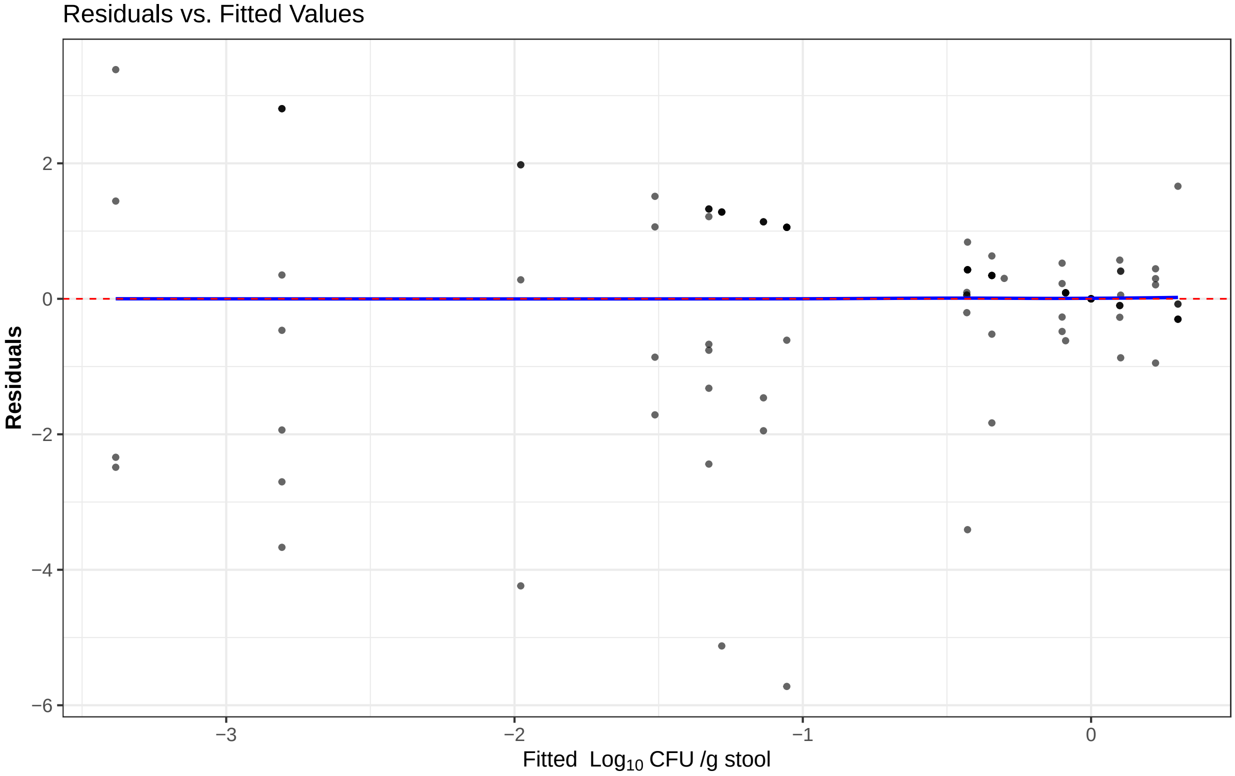

1.7.1.4 Balanced Data

There is one `mice` that has a measurement missing, and has been excluded from the analysis.

1.7.2 Results

Repeated-Measures ANOVA Results

	DFn	DFd	SSn	SSd	F	p-value	p<.05	ges	
(Intercept)	1	24	2223.97400	148.1355	360.31	0.000	*	0.8436843	*
group	4	24	268.93696	148.1355	10.89	0.000	*	0.3949209	*
day	5	120	97.41118	263.9169	8.86	0.000	*	0.1912034	*
group:day	20	120	100.41718	263.9169	2.28	0.003	*	0.1959476	*

Mauchly's Test for Sphericity

	W	p-value	p<.05
day	0.004	0.000	*

group:day	0.004	0.000	*

Sphericity Corrections

	ϵ_{GG}	p[GG]	p[GG]<.05	ϵ_{HF}	p[HF]	p[HF]<.05
day	0.464	0.000	*	0.517	0.000	*
group:day	0.464	0.028	*	0.517	0.023	*

Greenhouse–Geisser DF Adjustment

	df_1	df_2	ϵ_{GG}	$df_1 \times \epsilon_{GG}$	$df_2 \times \epsilon_{GG}$
Day	5	120	0.464	2.32	55.68
Group x Day	20	120	0.464	9.28	55.68

Dunnett Post-hoc Comparisons for Gram Negatives

Each antibiotic group vs. saline at each post-treatment day

	Estimate	SE	DF	Lower 95% CI	Upper 95% CI	t-ratio	p-value
**Baseline**							
1 Day - Saline	−4.19	1.13	114	−7.07	−1.31	−3.69	0.001
3 Day - Saline	−4.39	1.20	114	−7.42	−1.35	−3.67	0.001
6 Day - Saline	−3.27	1.04	114	−5.90	−0.64	−3.16	0.004
10 Day - Saline	−3.05	1.04	114	−5.68	−0.42	−2.95	0.004
**1 Day Post-Treatment**							
1 Day - Saline	−4.18	1.13	114	−7.06	−1.30	−3.69	0.001
3 Day - Saline	−4.18	1.20	114	−7.22	−1.15	−3.50	0.001
6 Day - Saline	−4.18	1.04	114	−6.81	−1.55	−4.04	0.000
10 Day - Saline	−4.18	1.04	114	−6.81	−1.55	−4.04	0.000
**3 Day Post-Treatment**							
1 Day - Saline	−4.33	1.13	114	−7.21	−1.45	−3.81	0.000
3 Day - Saline	−4.33	1.20	114	−7.36	−1.29	−3.62	0.000
6 Day - Saline	−4.33	1.04	114	−6.95	−1.70	−4.18	0.000
10 Day - Saline	−4.20	1.04	114	−6.83	−1.57	−4.05	0.000
**7 Day Post-Treatment**							
1 Day - Saline	−3.21	1.13	114	−6.09	−0.33	−2.83	0.011
3 Day - Saline	−2.36	1.20	114	−5.40	0.68	−1.97	0.051
6 Day - Saline	−3.49	1.04	114	−6.12	−0.86	−3.37	0.003
10 Day - Saline	−4.19	1.04	114	−6.82	−1.57	−4.05	0.000
**14 Day Post-Treatment**							
1 Day - Saline	−2.48	1.13	114	−5.36	0.40	−2.18	0.093
3 Day - Saline	0.40	1.20	114	−2.63	3.44	0.34	1.000
6 Day - Saline	−0.64	1.04	114	−3.27	1.99	−0.62	1.000
10 Day - Saline	−3.68	1.04	114	−6.31	−1.05	−3.55	0.002
**22 Day Post-Treatment**							

1 Day - Saline	−3.41	1.13	114	−6.29	−0.53	−3.01	0.010
3 Day - Saline	−0.67	1.20	114	−3.71	2.36	−0.56	0.574
6 Day - Saline	−1.43	1.04	114	−4.06	1.20	−1.38	0.342
10 Day - Saline	−4.15	1.04	114	−6.78	−1.52	−4.01	0.000

1.7.3 Conclusions

1.8 Repeated Measures ANOVA (Enterococci)

1.8.1 Model Diagnostics

The repeated measures ANOVA has the following assumptions:

- **Independence of subjects** - Each mouse's response is independent of every other's
- **Normality of residuals** - The within-subject residuals (deviations from each mouse's group by day mean are approximately normally distributed)
- **Sphericity (Homogeneity of covariances)** - The variances of the pairwise differences between levels of the within subject factor (`day`) are equal
- **Homoscedasticity of between-subjects factor** - The variance of observations across the levels of the between-subjects factor (`group`) is approximately equal
- **Balanced Design** - The within-subject levels are categorical and each subject has the same measurements at every level

1.8.1.1 Normality of Residuals

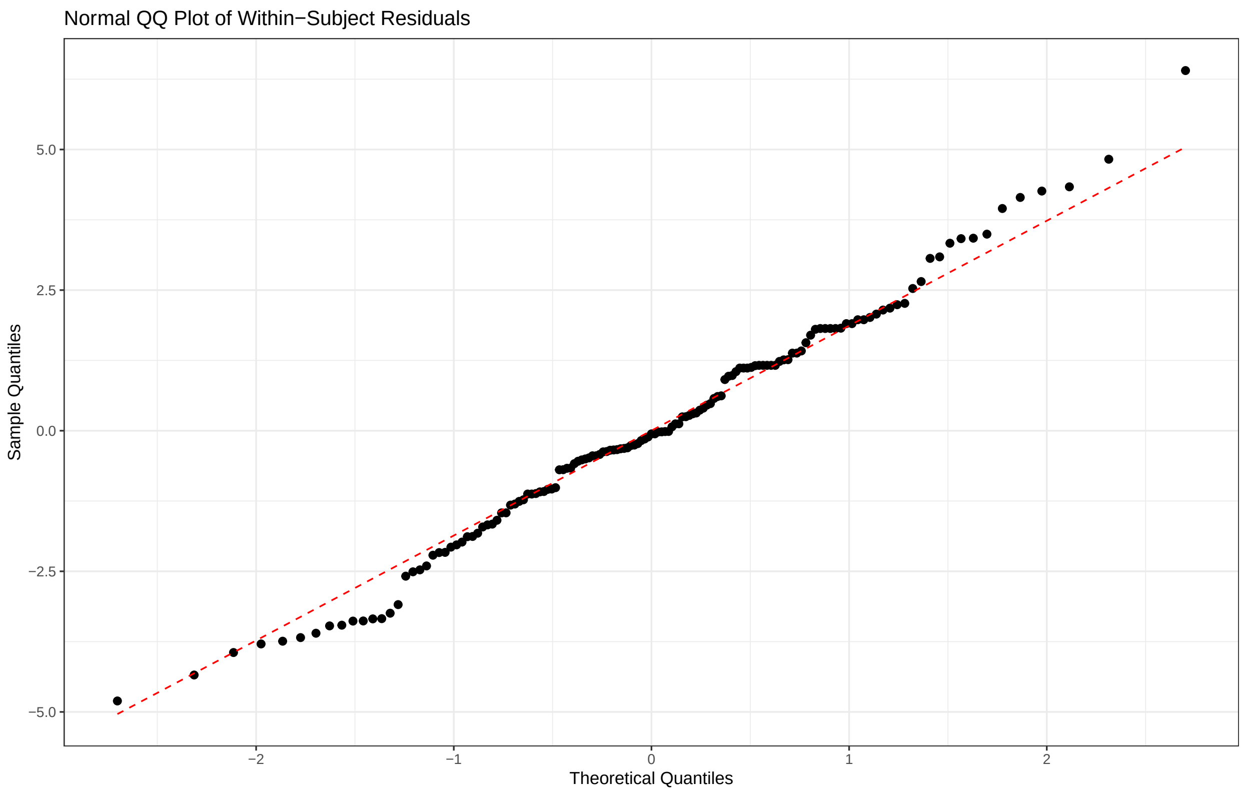

1.8.1.2 Sphericity

Mauchly's Test for Sphericity

	W	p-value	p<.05
day	0.139	0.000	*
group:day	0.139	0.000	*

Sphericity Corrections

	ϵ_{GG}	p[GG]	p[GG]<.05	ϵ_{HF}	p[HF]	p[HF]<.05
day	0.639	0.000	*	0.749	0.000	*
group:day	0.639	0.040	*	0.749	0.030	*

1.8.1.3 Homoscedasticity of Between-subjects Factor

Levene's Test for Homogeneity of Variance (center = median)

Df	F value	Pr(>F)
4	3.349036	0.01143684
169		

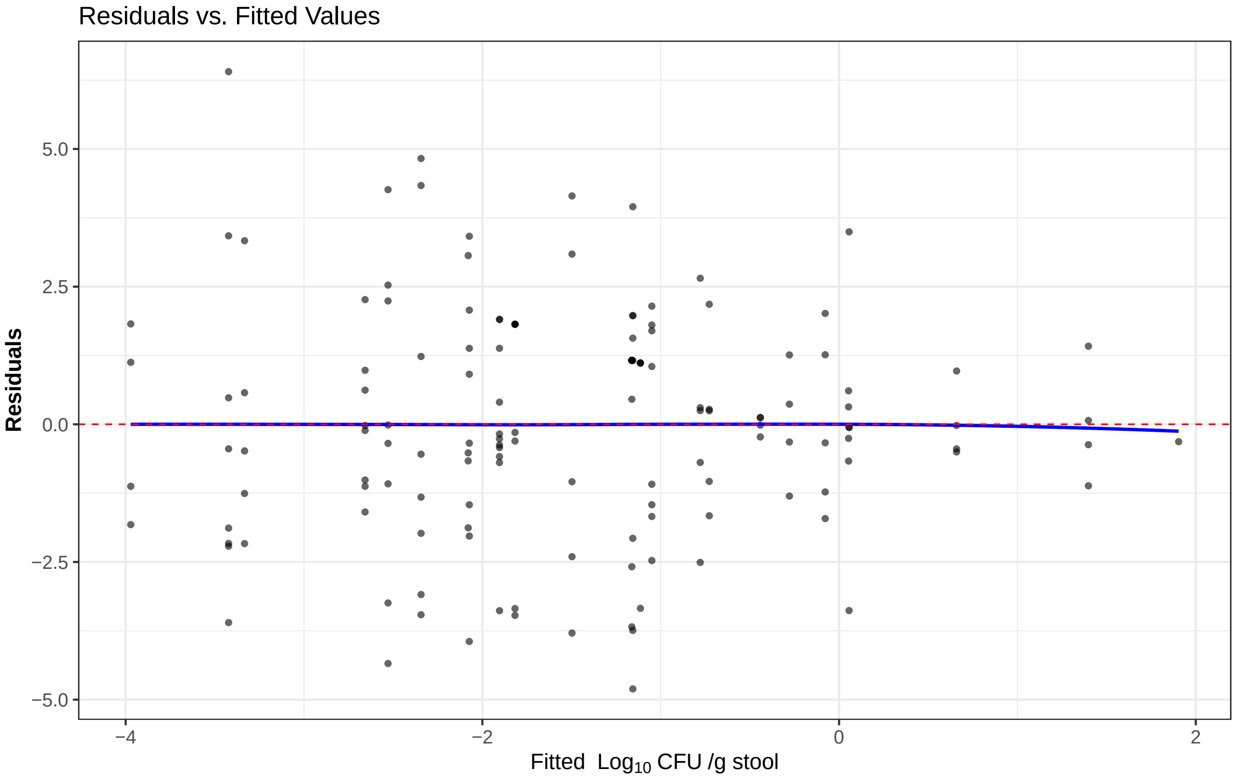

1.8.1.4 Balanced Data There is one `mice` that has a measurement missing, and has been excluded from the analysis.

1.8.2 Results

Repeated-Measures ANOVA Results

	DFn	DFd	SSn	SSd	F	p-value	p<.05	ges	
(Intercept)	1	24	4985.0977	298.2707	401.12	0.000	*	0.8458009	*
group	4	24	122.8862	298.2707	2.47	0.072		0.1191074	
day	5	120	251.1708	610.5693	9.87	0.000	*	0.2165245	*
group:day	20	120	196.0887	610.5693	1.93	0.016	*	0.1774673	*

Mauchly's Test for Sphericity

	W	p-value	p<.05
day	0.139	0.000	*
group:day	0.139	0.000	*

Sphericity Corrections

	ϵ_{GG}	p[GG]	p[GG]<.05	ϵ_{HF}	p[HF]	p[HF]<.05
day	0.639	0.000	*	0.749	0.000	*
group:day	0.639	0.040	*	0.749	0.030	*

Greenhouse–Geisser DF Adjustment

	df_1	df_2	ϵ_{GG}	$df_1 \times \epsilon_{GG}$	$df_2 \times \epsilon_{GG}$
Day	5	120	0.639	3.20	76.68
Group x Day	20	120	0.639	12.78	76.68

Dunnett Post-hoc Comparisons for Enterococci

Each antibiotic group vs. saline at each post-treatment day

	Estimate	SE	DF	Lower 95% CI	Upper 95% CI	t-ratio	p-value
Baseline							
1 Day - Saline	−5.55	1.69	121	−9.82	−1.28	−3.29	0.003
3 Day - Saline	−5.55	1.78	121	−10.06	−1.05	−3.12	0.003
6 Day - Saline	−5.55	1.54	121	−9.45	−1.65	−3.61	0.002
10 Day - Saline	−5.55	1.54	121	−9.45	−1.65	−3.61	0.002
1 Day Post-Treatment							
1 Day - Saline	0.09	1.69	121	−4.18	4.37	0.06	0.955
3 Day - Saline	−3.04	1.78	121	−7.55	1.46	−1.71	0.267
6 Day - Saline	−2.98	1.54	121	−6.88	0.92	−1.93	0.222
10 Day - Saline	−2.05	1.54	121	−5.95	1.85	−1.33	0.370
3 Day Post-Treatment							
1 Day - Saline	−0.83	1.69	121	−5.10	3.44	−0.49	1.000
3 Day - Saline	−1.90	1.78	121	−6.40	2.60	−1.07	0.860

6 Day - Saline	−2.78	1.54	121	−6.68	1.12	−1.81	0.292
10 Day - Saline	0.46	1.54	121	−3.44	4.36	0.30	1.000
7 Day Post-Treatment							
1 Day - Saline	−1.71	1.69	121	−5.98	2.57	−1.01	0.940
3 Day - Saline	−4.07	1.78	121	−8.58	0.43	−2.29	0.094
6 Day - Saline	−1.36	1.54	121	−5.26	2.54	−0.88	0.940
10 Day - Saline	−0.18	1.54	121	−4.09	3.72	−0.12	0.940
14 Day Post-Treatment							
1 Day - Saline	0.38	1.69	121	−3.89	4.65	0.22	1.000
3 Day - Saline	2.36	1.78	121	−2.14	6.87	1.33	0.559
6 Day - Saline	0.71	1.54	121	−3.19	4.61	0.46	1.000
10 Day - Saline	2.70	1.54	121	−1.20	6.60	1.76	0.326
22 Day Post-Treatment							
1 Day - Saline	−1.74	1.69	121	−6.02	2.53	−1.03	1.000
3 Day - Saline	−0.47	1.78	121	−4.97	4.04	−0.26	1.000
6 Day - Saline	−1.55	1.54	121	−5.45	2.35	−1.00	1.000
10 Day - Saline	0.86	1.54	121	−3.05	4.76	0.56	1.000

1.8.3 Conclusions

1.9 Linear Mixed Model (Gram Negatives)

1.9.1 Model Diagnositics

Assumptions include:

- **Linearity** - The expected CFU change is a linear function of the fixed predictors. If this assumption is violated then β estimates will be biased.
- **Normality of Level-1 Residuals** - After accounting for fixed effects and random effects, the remaining residuals are approximately normal. Violations of this assumption causes p-values and confidence intervals for the fixed-effect to be biased.
- **Homoscedasticity of Residuals** - The variance of the residuals should be constant across levels of predictors. If residuals systematically change then standard error and test statistics can be wrong
- **Independence of Level-1 Errors** - Residuals (after accounting for random effects) are uncorrelated across observations. Violations inflate type 1 error.
- **Correct Random-Effects Structure** - Every random intercept slope should be included in the model. Violations of this assumption lead to biased standard errors and overfitting.
- **Normality of Random Effects** - The distribution of the random intercepts should be normal. Mixed Models assumes random effects come from a normal distribution, and severe departures can bias variance estimates
- **No Extreme Multicollinearity** - The fixed predictors are not exact linear combinations of one another. Violations lead to inflated standard errors and results in unstable coefficients.
- **Proper Nesting / Independence of Clusters** - The group factor (`mice`) should correctly partition the data into independent clusters, with no mouse appearing in two different clusters
- **No Missing Data** - Missing observations (if any) should be missing at random (MAR), so model-based likelihood inference remains valid.
- **No influential observations or outliers** - No single mouse or single observation should greatly impact the fixed-effect estimates

1.9.1.1 Linearity

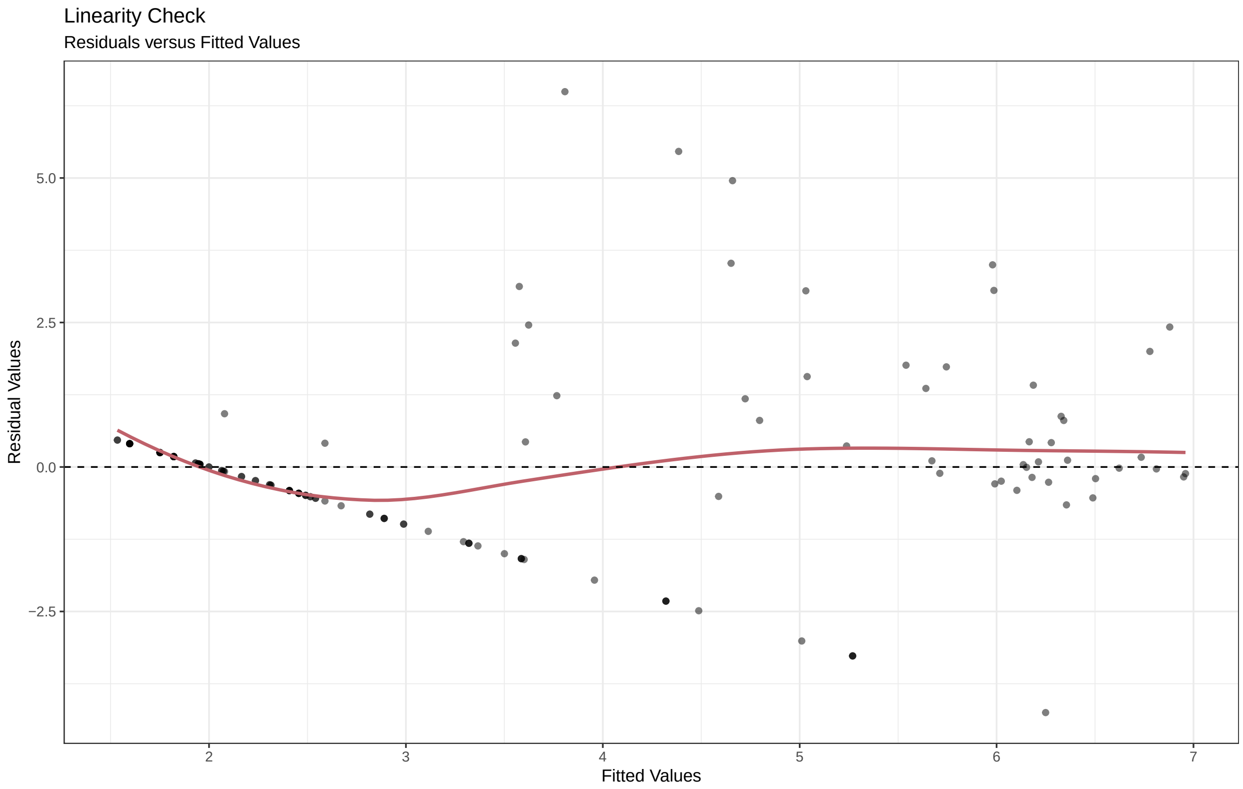

1.9.1.2 Homoscedastictiy (Constant Variance of Residuals)

Homoscedasticity means that the residuals have constant variance across levels of fitted values or predictors. The following will look to ensure that the spread of residuals remains roughly the same regardless of the predicted value.

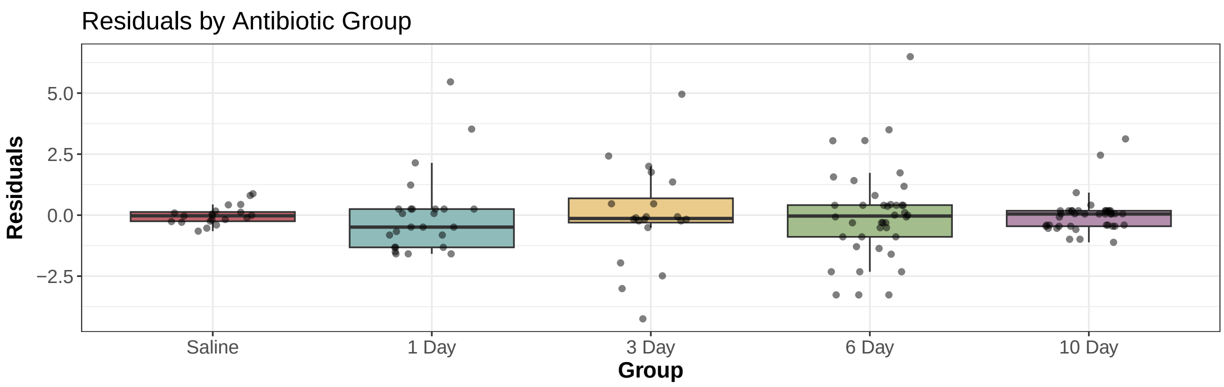

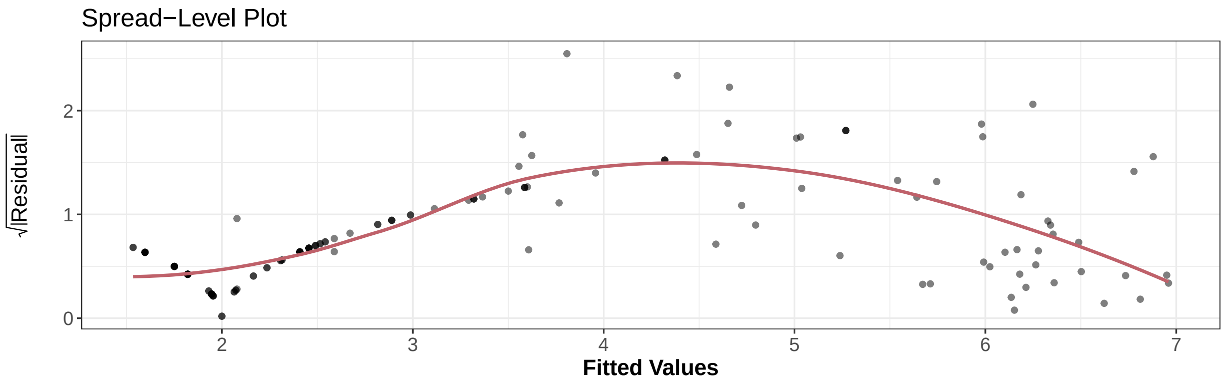

1.9.1.3 Normality of Level-1 Residuals

The normality of residuals are needed to have valid hypothesis testing (i.e., p-values and confidence intervals). Deviation can occur with large samples, but major skewness or heavy tails may indicate problems with the model

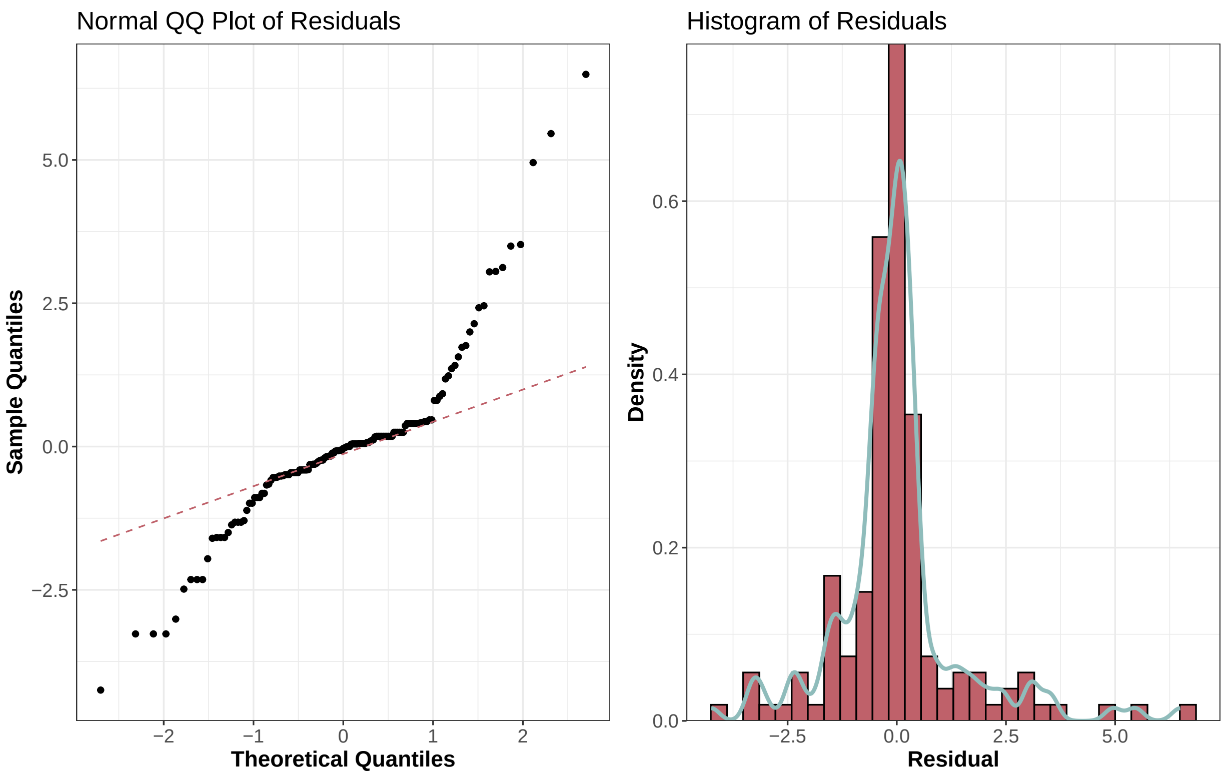

1.9.1.4 Independence of Residuals For a mixed effects model with repeated measures, the residuals should not be autocorrelated with one another (i.e., Residuals from one observation are not related to other across time within subjects).

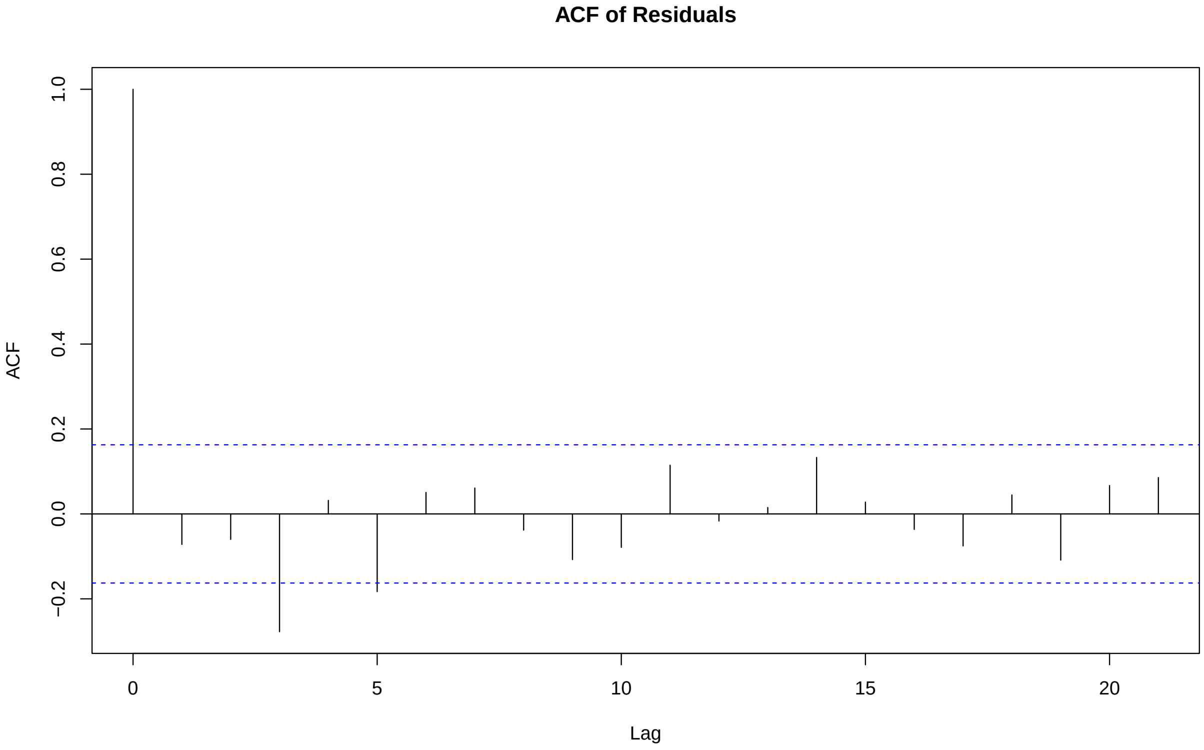


```
## OK: Residuals appear to be independent and not autocorrelated (p = 0.332).
```

1.9.1.5 Normality of Random Effects Mixed effect models assume that the random effects (i.e., the random intercepts for `mice`) are normally distributed. Violations of this can impact the estimation of variance components and confidence intervals for fixed effects.

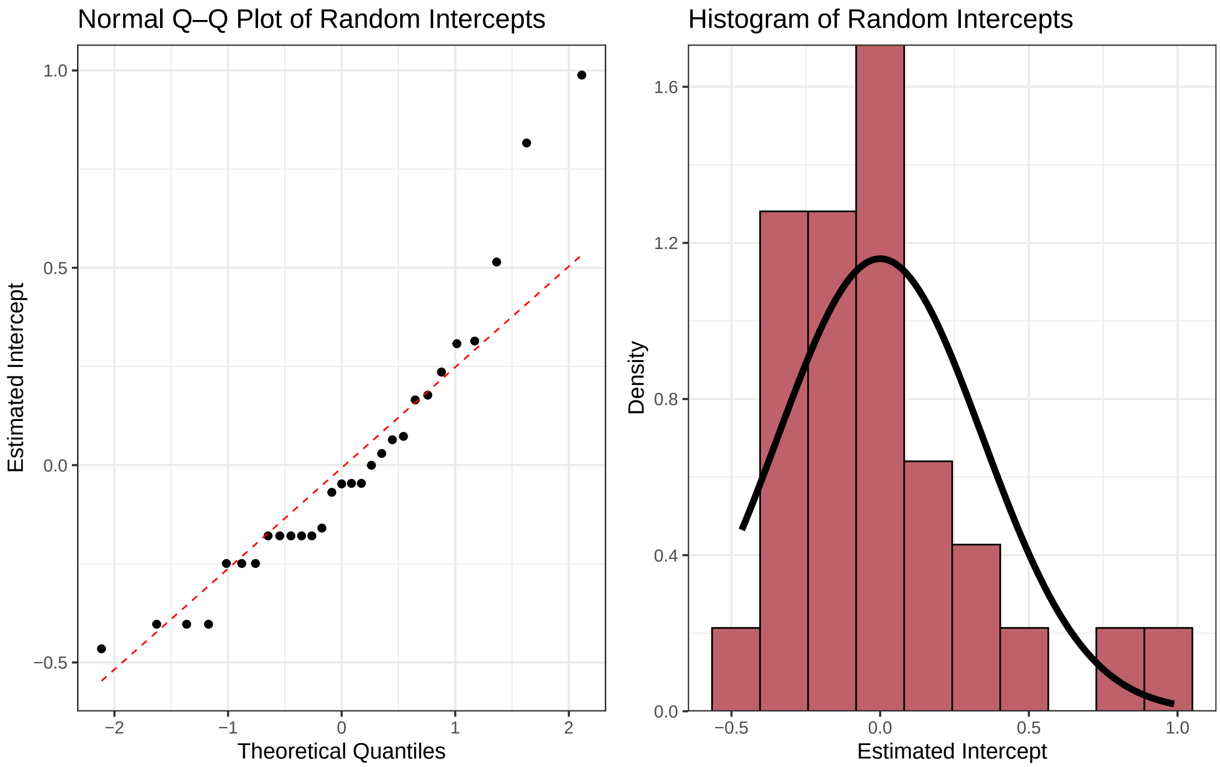

1.9.1.6 Multicollinearity Multicollinearity between predictors can inflate standard errors, influence coefficients and cause instability in the model estimations.

term	GVIF	Df	GVIF^(1/(2*Df))
group	1.000000	4	1.000000
day	1.625204	5	1.049762
group:day	1.625204	20	1.012215

1.9.1.7 Properly Nested Data

Linear mixed effect models require that each cluster (i.e. `mice`) is independent of the other clusters and that observations within each cluster (i.e. repeated measure) are correctly grouped under the random-effects structure. Violations can lead to inflated degrees of freedom, biased variance estimates, and invalid standard errors.

All `mice` ID appear in exactly one group (no mouse switches between antibiotic conditions) and each mice has the same number of observations (one per level of day per organism), so the repeated measures structure is balanced and nested. This indicates our data is properly nested for a random-intercept model.

1.9.1.8 Outliers If the model contains extreme observations this can influence the model leading to influencing fixed-effect estimates and bias model diagnostics.

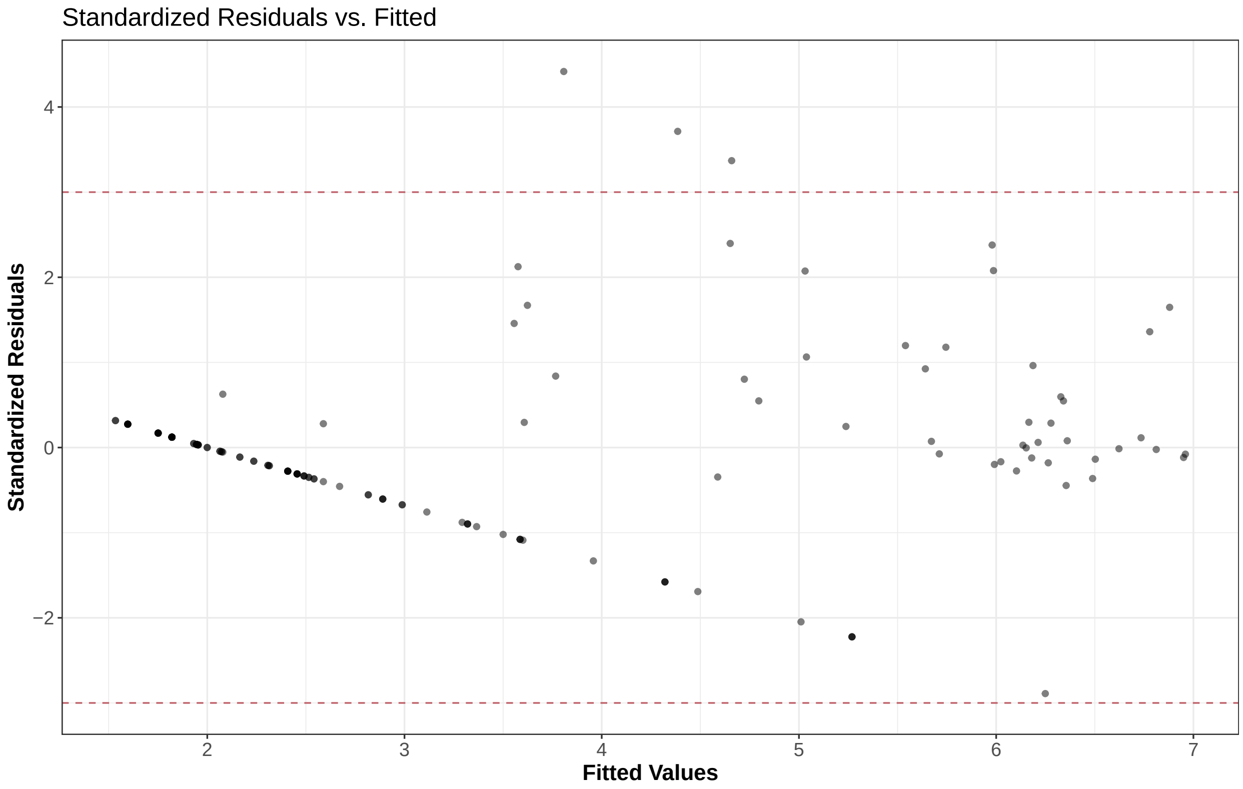

1.9.2 Results

Fixed Effects Estimates – Linear Mixed Model

Modeling CFU by group × day (Gram Negatives)

Term	Estimate	SE	Lower 95% CI	Upper 95% CI	z	p-value
(Intercept)	3.82	0.18	3.46	4.18	20.89	0.000
1 Day Abx	2.53	0.29	1.92	3.13	8.61	0.000
3 Day Abx	−0.99	0.39	−1.80	−0.18	−2.53	0.019
6 Day Abx	0.30	0.49	−0.71	1.31	0.61	0.546
10 Day Abx	−0.28	0.35	−1.00	0.43	−0.82	0.420
3	−0.99	0.30	−1.57	−0.40	−3.33	0.001
7	−0.93	0.30	−1.52	−0.34	−3.14	0.002
14	0.31	0.30	−0.28	0.90	1.04	0.300
22	1.21	0.30	0.62	1.80	4.09	0.000
1 Day Abx × 3	0.82	0.34	0.14	1.50	2.39	0.019
3 Day Abx × 3	0.16	0.65	−1.14	1.45	0.24	0.811
6 Day Abx × 3	−1.14	0.86	−2.84	0.56	−1.33	0.188
10 Day Abx × 3	−0.55	0.59	−1.72	0.61	−0.94	0.348
1 Day Abx × 7	0.91	0.34	0.23	1.59	2.65	0.009
3 Day Abx × 7	0.10	0.65	−1.19	1.40	0.16	0.875
6 Day Abx × 7	−1.19	0.86	−2.89	0.51	−1.39	0.168
10 Day Abx × 7	−0.61	0.59	−1.77	0.56	−1.03	0.304
1 Day Abx × 14	0.12	0.34	−0.56	0.80	0.36	0.721
3 Day Abx × 14	0.43	0.65	−0.86	1.73	0.66	0.511
6 Day Abx × 14	−0.01	0.86	−1.71	1.69	−0.01	0.992
10 Day Abx × 14	−0.55	0.59	−1.72	0.61	−0.94	0.347

1 Day Abx × 22	−1.25	0.34	−1.93	−0.57	−3.66	0.000
3 Day Abx × 22	−0.21	0.65	−1.50	1.09	−0.31	0.754
6 Day Abx × 22	1.38	0.86	−0.32	3.08	1.61	0.111
10 Day Abx × 22	0.92	0.59	−0.24	2.09	1.57	0.119

A linear mixed effects model was fitted with fixed effects for antibiotic group (`group`) and the day for when the sample was taken `day` and an interaction effect between `group` and `day` in predicting $\log_{10}$ CFU levels of gram negatives in mice stool. A random intercept was incorporated for each mice to allow them to have their own average baseline CFU levels and to account for repeated measures.

Post-Hoc Contrasts (Gram Negatives)

Each antibiotic group compared to Saline

	Estimate	SE	DF	Lower 95% CI	Upper 95% CI	t-ratio	p-value
**1 Day Post-Treatment**							
1 Day - Saline	−4.18	0.95	26	−6.74	−1.63	−4.40	0.000
3 Day - Saline	−4.18	1.25	18	−7.66	−0.71	−3.35	0.004
6 Day - Saline	−4.18	0.84	43	−6.38	−1.99	−4.97	0.000
10 Day - Saline	−4.18	0.52	9	−5.79	−2.58	−8.05	0.000
**3 Day Post-Treatment**							
1 Day - Saline	−4.33	0.95	26	−6.88	−1.77	−4.55	0.000
3 Day - Saline	−4.33	1.25	18	−7.80	−0.85	−3.46	0.003
6 Day - Saline	−4.33	0.84	43	−6.52	−2.13	−5.14	0.000
10 Day - Saline	−4.20	0.52	9	−5.81	−2.59	−8.09	0.000
**7 Day Post-Treatment**							
1 Day - Saline	−3.21	0.95	26	−5.77	−0.66	−3.38	0.005
3 Day - Saline	−2.36	1.25	18	−5.83	1.11	−1.89	0.076
6 Day - Saline	−3.49	0.84	43	−5.68	−1.30	−4.15	0.000
10 Day - Saline	−4.19	0.52	9	−5.80	−2.59	−8.08	0.000
**14 Day Post-Treatment**							
1 Day - Saline	−2.48	0.95	26	−5.03	0.08	−2.61	0.045
3 Day - Saline	0.40	1.25	18	−3.07	3.88	0.32	0.902
6 Day - Saline	−0.64	0.84	43	−2.83	1.55	−0.76	0.902
10 Day - Saline	−3.68	0.52	9	−5.28	−2.07	−7.08	0.000
**22 Day Post-Treatment**							
1 Day - Saline	−3.41	0.95	26	−5.96	−0.86	−3.59	0.004
3 Day - Saline	−0.67	1.25	18	−4.15	2.80	−0.54	0.596
6 Day - Saline	−1.43	0.84	43	−3.62	0.77	−1.70	0.194
10 Day - Saline	−4.15	0.52	9	−5.76	−2.54	−7.99	0.000

To quantify the impact of antibiotic duration on the suppression and recovery of gram-negative bacteria, Dunnett-adjusted pairwise comparisons between each antibiotic group and the saline controls were conducted across post-treatment days 1, 3, 7, 14, and 22.

Across all early time points (Days 1-3) all antibiotic showed significantly slower $\log_{10}$ CFU counts compared to the saline group confirming strong and sustained depletion of gram-negative flora following antibiotic exposure. One day after stopping treatment, all $\log_{10}$ CFU reductions relative to saline were highly significant across all antibiotic durations ($p < 0.001$) with an average difference around -4,2 $\log_{10}$ CFU. This reduction persisted across to day 3 with a slight increase in the average difference and all antibiotic groups remaining

significantly different (all $p < 0.003$). By day 7, the 1, 6, and 10 day antibiotic groups remained significantly lower compared to the saline group (all $p < 0.005$). However, the 3-day antibiotic treatment group remained lower but non-significantly different from the saline controls (δ = -2.36; SE = 1.25; 95% CI [-5.83, 1.12]; p = 0.076). By day 14, partial recovery was evident for some groups. The 1-day group remained significantly below saline (δ = -2.48; 95% CI [-5.03, 0.08]; p = 0.045). The 3 and 6 day antibiotic treatment duration groups showed no significant difference from the saline group (p = 0.902). However, the 10-day group continued to exhibit a substantial and significant suppression (δ = -3.68; 95% CI [-5.28, -2.07]; $p < 0.001$), indicating prolong disruption of gram-negative colonization. By day 22 after treatment, a similar pattern emerged. The 3 and 6 day groups did had lower but non-significantly different differences in gram-negative concentration from the saline group (both $p > 0.194$) suggesting nearly full recovery of gram-negatives by this point. Interestingly, the 1-day group surprisingly remained significantly suppressed (δ = -3.41; 95% CI [-5.96, -0.82]; p = 0.004) while the 10-day group still exhibited a large and highly significant reduction ($p <$ 0.001), indicating delayed or incomplete recovery following extended antibiotic exposure.

These results suggest that all durations of antibiotic treatment produce acute suppression of gram-negative bacteria with greater duration associated with a more prolonged and pronounced suppresive effect. Recovery appears largely complete by day 14 in the 3 and 6 day antibiotic groups, while significant deficits remain in the 10 day group up to 22 days after stopping treatment, reflecting duration-dependent disruption and delayed microbial rebound. The unexpected sustained suppression in the 1 day group up to day 22 may reflect residual microbial shifts or stochastic recolonization dynamics, warranting further investigation.

Overall Group Comparisons for Gram Negatives

Comparisons of overall differences for antibiotics group in colonization

	Estimate	SE	DF	Lower 95% CI	Upper 95% CI	t-ratio	p-value
Saline - 1 Day	3.52	0.54	10	1.62	5.43	6.55	0.001
Saline - 3 Day	2.23	0.66	12	−0.01	4.47	3.38	0.037
Saline - 6 Day	2.81	0.48	8	1.00	4.63	5.83	0.003
Saline - 10 Day	4.08	0.38	3	1.05	7.12	10.72	0.018
1 Day - 3 Day	−1.29	0.74	27	−3.55	0.97	−1.75	0.366
1 Day - 6 Day	−0.71	0.59	33	−2.48	1.06	−1.20	0.710
1 Day - 10 Day	0.56	0.51	16	−1.09	2.21	1.10	0.710
3 Day - 6 Day	0.58	0.70	25	−1.57	2.74	0.84	0.710
3 Day - 10 Day	1.85	0.63	16	−0.21	3.92	2.92	0.061
6 Day - 10 Day	1.27	0.45	15	−0.21	2.74	2.83	0.064

To assess cumulative differences in gram-negative colonization across treatment groups, overall pairwise comparisons aggregated across all post-treatment time points were conducted. These comparisons reflect the average difference in $\log_{10}$ CFU burden across the entire experimental period. The results found that all antibiotic-treated groups showed significantly lower gram-negative colonization than the saline group (all $p <$ 0.037). These findings confirm that antibiotic treatment suppresses gram-negative colonization relative to the saline controls, with the largest suppression observed in the 10-day group. Direct comparisons between antibiotic groups were not statistically significant. The largest difference was the 3-day antibiotic group having an average of 1.85 $\log_{10}$ CFU (δ = 1.85, SE = 0.63; 95% CI [-0.22, 3.92]; p = 0.061) more gram negatives compared to the 10-day group. This suggest that greater cumulative suppression occurs with longer antibiotic courses.

These results reinforce that antibiotic exposure significantly reduces gram-negative bacterial colonization, with a duration dependent effect. The day day regimen resulted in the strongest and most sustained suppression. While the 1-day group also showed exceptional reductions compared to the saline group. differences between short and long durations were less pronounced across the full recovery window. The lack of full convergence across antibiotics that even brief exposures with antibiotic treatment can induce lasting microbial disruption, although longer treatment courses amplify this effect.

1.9.3 Conclusions

Antibiotic treatment caused acute and duration-dependent suppression of gram-negative bacteria, with all regimens producing significantly lower colonization levels compared to saline controls during the early post antibiotic treatment period. Recovery trajectories varied by duration: shorter treatments showed earlier rebound while longer exposures (10-day) resulted in sustained exhaustion of gram negatives up to 22 days after cessation of treatment. Overall comparisons confirmed that all antibiotic groups had reduced cumulative gram-negative colonization relative to the controls, with the 10-day group exhibiting the most prominent suppression. While differences between antibiotics groups were generally not statistically significant across the full experimental trail, trends suggested greater microbiol disruption with increased antibiotic duration. However, even shorter courses of antibiotics (1-day) were found to have a pronounced effects on the gram-negative flora within the mice.

1.10 Linear Mixed Model (Enterococci)

1.10.1 Model Diagnositics

Assumptions include:

- **Linearity** - The expected CFU change is a linear function of the fixed predictors. If this assumption is violated then β estimates will be biased.
- **Normality of Level-1 Residuals** - After accounting for fixed effects and random effects, the remaining residuals are approximately normal. Violations of this assumption causes p-values and confidence intervals for the fixed-effect to be biased.
- **Homoscedasticity of Residuals** - The variance of the residuals should be constant across levels of predictors. If residuals systematically change then standard error and test statistics can be wrong
- **Independence of Level-1 Errors** - Residuals (after accounting for random effects) are uncorrelated across observations. Violations inflate type 1 error.
- **Correct Random-Effects Structure** - Every random intercept slope should be included in the model. Violations of this assumption lead to biased standard errors and overfitting.
- **Normality of Random Effects** - The distribution of the random intercepts should be normal. Mixed Models assumes random effects come from a normal distribution, and severe departures can bias variance estimates
- **No Extreme Multicollinearity** - The fixed predictors are not exact linear combinations of one another. Violations lead to inflated standard errors and results in unstable coefficients.
- **Proper Nesting / Independence of Clusters** - The group factor (`mice`) should correctly partition the data into independent clusters, with no mouse appearing in two different clusters
- **No Missing Data** - Missing observations (if any) should be missing at random (MAR), so model-based likelihood inference remains valid.
- **No influential observations or outliers** - No single mouse or single observation should greatly impact the fixed-effect estimates

1.10.1.1 Linearity

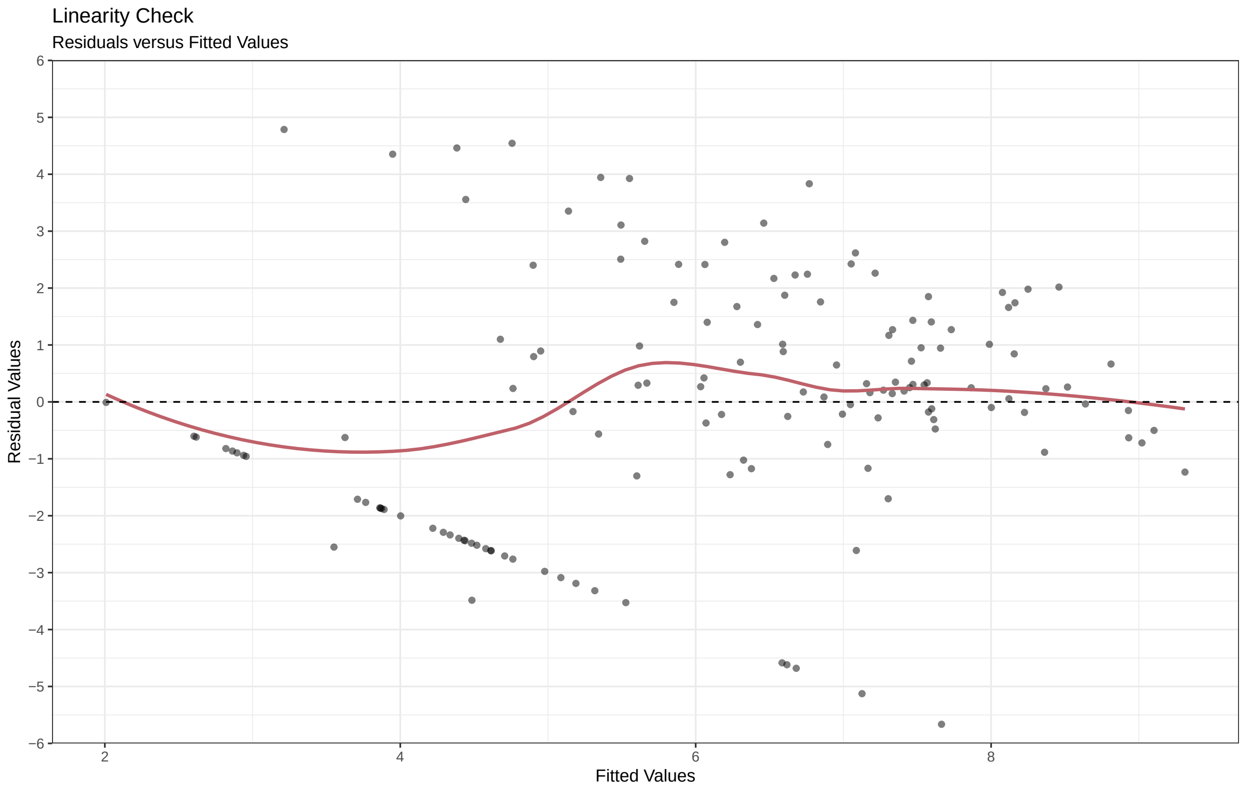

1.10.1.2 Homoscedastictiy (Constant Variance of Residuals)

Homoscedasticity means that the residuals have constant variance across levels of fitted values or predictors. The following will look to ensure that the spread of residuals remains roughly the same regardless of the predicted value.

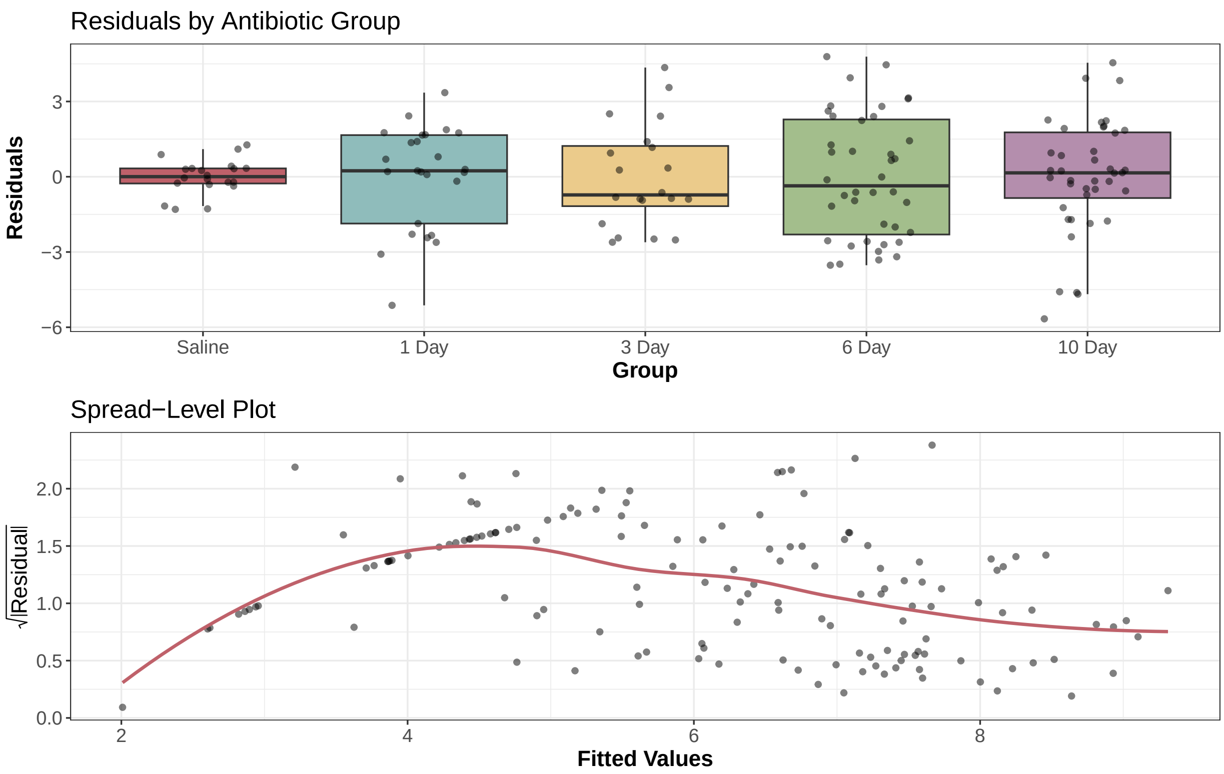

1.10.1.3 Normality of Level-1 Residuals

The normality of residuals are needed to have valid hypothesis testing (i.e., p-values and confidence intervals). Deviation can occur with large samples, but major skewness or heavy tails may indicate problems with the model

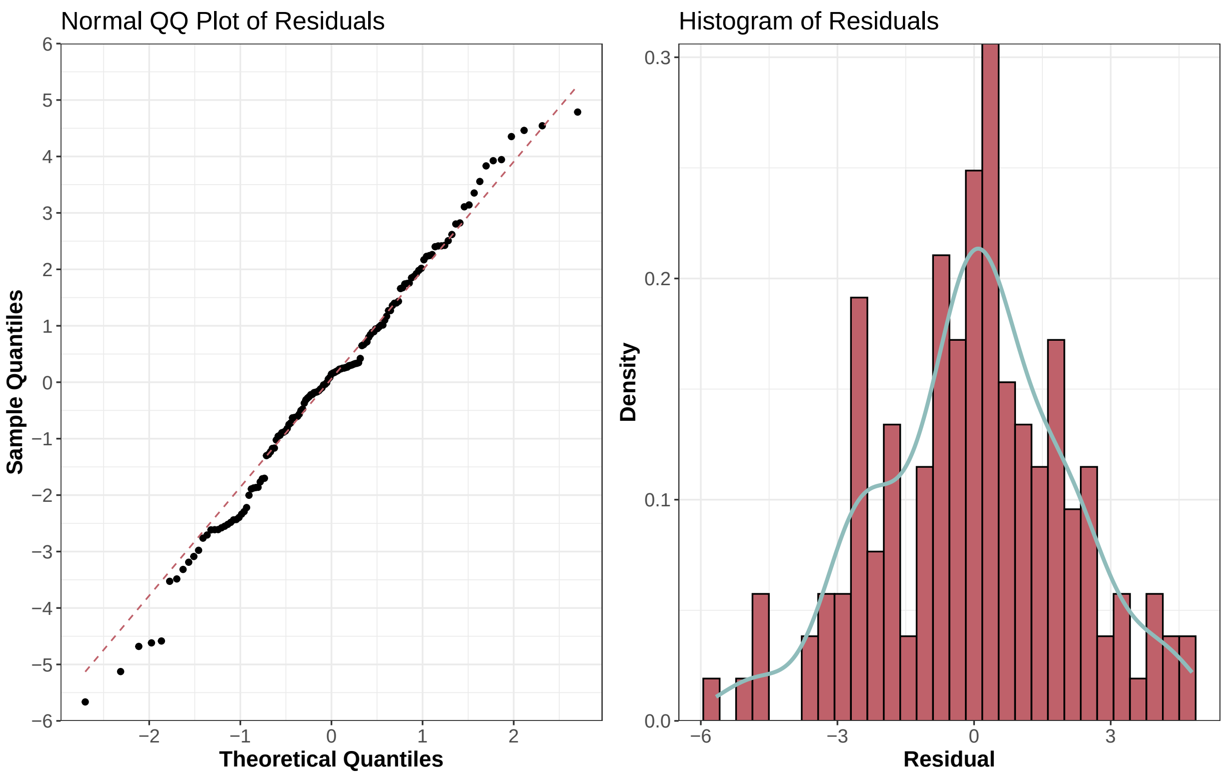

1.10.1.4 Independence of Residuals For a mixed effects model with repeated measures, the residuals should not be autocorrelated with one another (i.e., Residuals from one observation are not related to other across time within subjects).

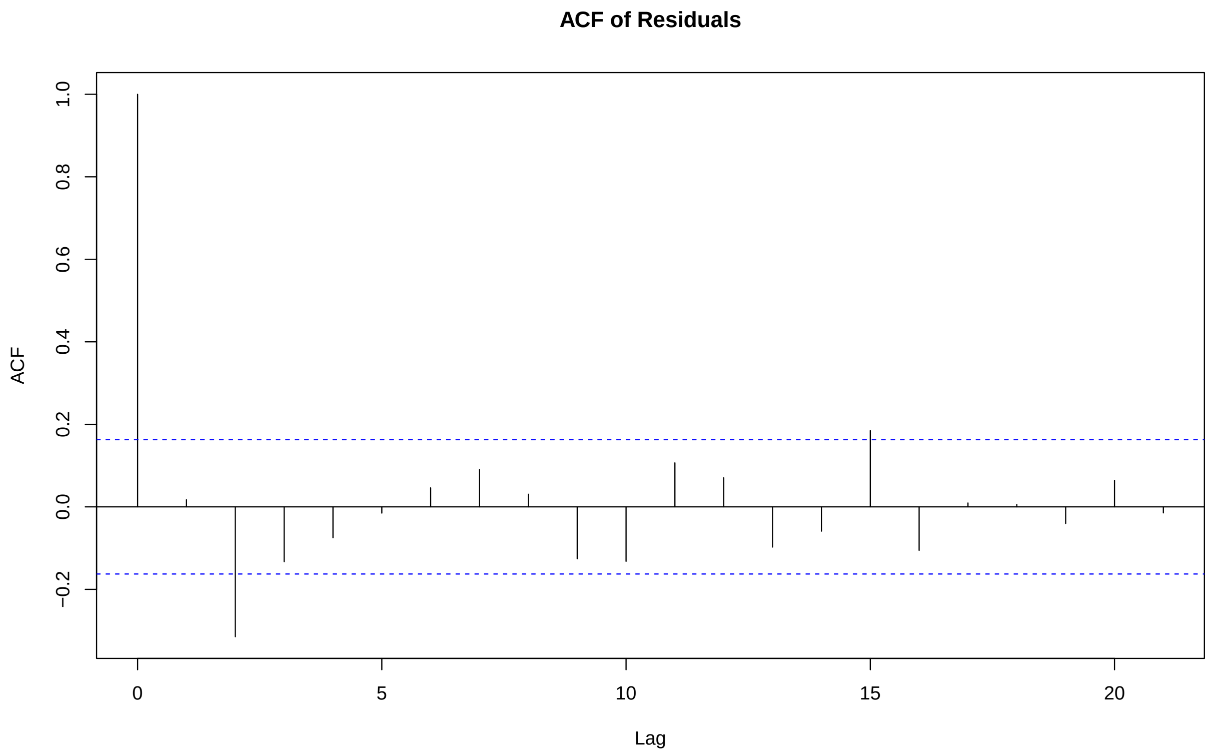


```
## Warning: Autocorrelated residuals detected (p = 0.038).
```

1.10.1.5 Normality of Random Effects Mixed effect models assume that the random effects (i.e., the random intercepts for `mice`) are normally distributed. Violations of this can impact the estimation of variance components and confidence intervals for fixed effects.

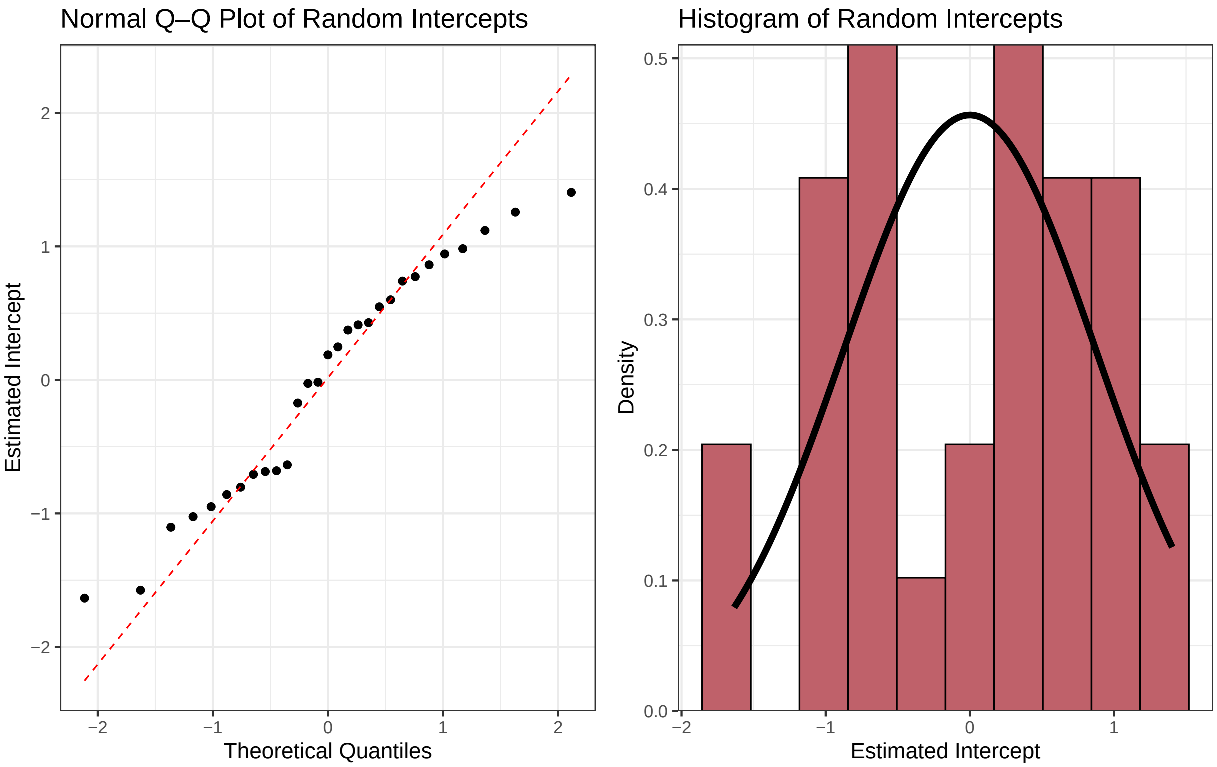

1.10.1.6 Multicollinearity Multicollinearity between predictors can inflate standard errors, influence coefficients and cause instability in the model estimations.

term	GVIF	Df	GVIF^(1/(2*Df))
group	1.000000	4	1.000000
day	1.474777	4	1.049762
group:day	1.474777	16	1.012215

1.10.1.7 Properly Nested Data

Linear mixed effect models require that each cluster (i.e. `mice`) is independent of the other clusters and that observations within each cluster (i.e. repeated measure) are correctly grouped under the random-effects structure. Violations can lead to inflated degrees of freedom, biased variance estimates, and invalid standard errors.

All `mice` ID appear in exactly one group (no mouse switches between antibiotic conditions) and each mice has the same number of observations (one per level of day per organism), so the repeated measures structure is balanced and nested. This indicates our data is properly nested for a random-intercept model.

1.10.1.8 Outliers If the model contains extreme observations this can influence the model leading to influencing fixed-effect estimates and bias model diagnostics.

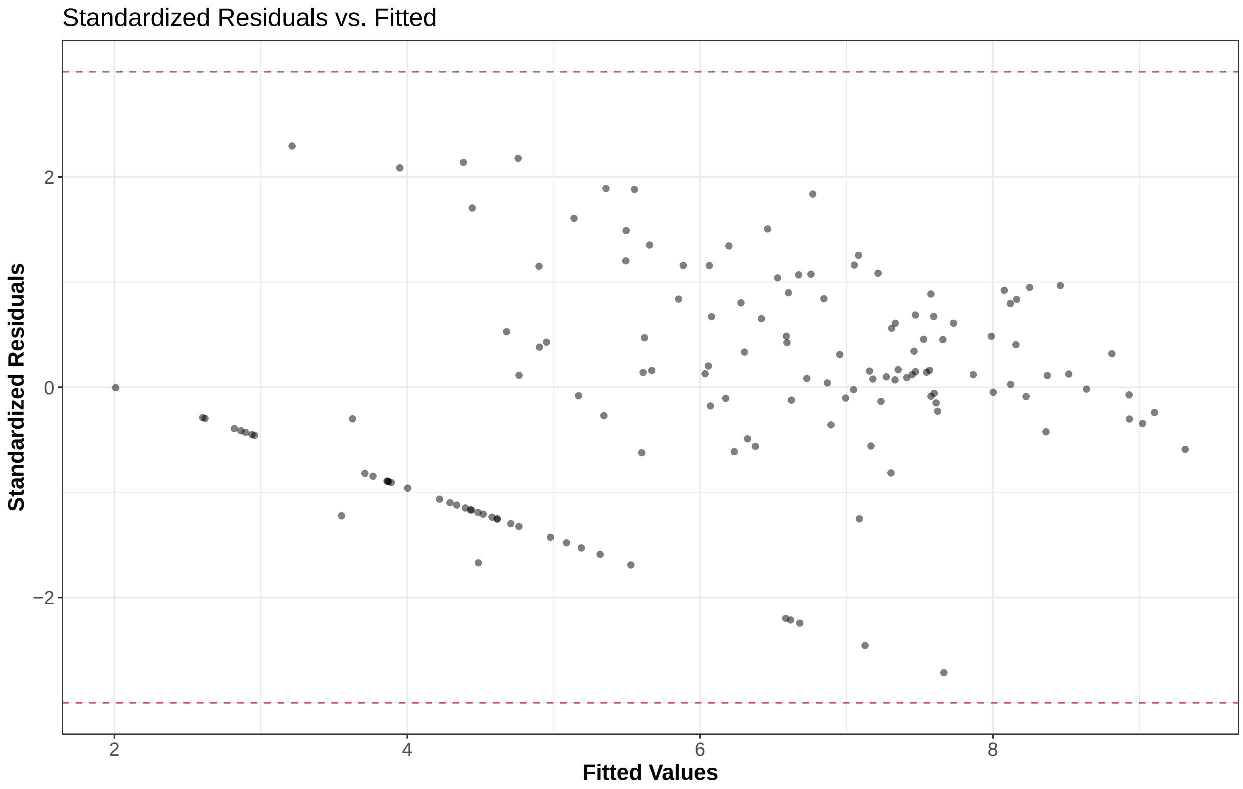

1.10.2 Results

Fixed Effects Estimates – Linear Mixed Model

Modeling CFU by group × day (Enterococci)

Term	Estimate	SE	Lower 95% CI	Upper 95% CI	z	p-value
(Intercept)	6.12	0.31	5.51	6.73	19.90	0.000
1 Day Abx	0.68	0.59	−0.53	1.90	1.16	0.257
3 Day Abx	−0.08	0.64	−1.40	1.25	−0.12	0.906
6 Day Abx	−0.74	0.71	−2.21	0.73	−1.04	0.308
10 Day Abx	−0.91	0.57	−2.07	0.26	−1.60	0.122
3	−1.10	0.38	−1.86	−0.34	−2.86	0.005
7	−0.11	0.38	−0.87	0.65	−0.29	0.771
14	−0.01	0.38	−0.77	0.75	−0.03	0.975
22	0.74	0.38	−0.02	1.50	1.93	0.057
1 Day Abx × 3	0.91	0.49	−0.06	1.88	1.87	0.065
3 Day Abx × 3	1.77	0.83	0.12	3.42	2.13	0.036
6 Day Abx × 3	−0.71	0.94	−2.57	1.15	−0.76	0.452
10 Day Abx × 3	−0.48	0.77	−2.00	1.05	−0.62	0.537
1 Day Abx × 7	0.33	0.49	−0.64	1.30	0.67	0.505
3 Day Abx × 7	0.26	0.83	−1.39	1.91	0.31	0.756
6 Day Abx × 7	−0.15	0.94	−2.01	1.71	−0.16	0.874
10 Day Abx × 7	−0.87	0.77	−2.39	0.66	−1.13	0.262
1 Day Abx × 14	0.78	0.49	−0.19	1.75	1.60	0.113
3 Day Abx × 14	−0.16	0.83	−1.81	1.49	−0.20	0.843
6 Day Abx × 14	−1.87	0.94	−3.73	−0.01	−1.99	0.049
10 Day Abx × 14	1.01	0.77	−0.51	2.53	1.32	0.191

1 Day Abx × 22	−1.92	0.49	−2.88	−0.95	−3.92	0.000
3 Day Abx × 22	−0.78	0.83	−2.43	0.87	−0.93	0.353
6 Day Abx × 22	1.87	0.94	0.01	3.73	2.00	0.049
10 Day Abx × 22	0.39	0.77	−1.13	1.91	0.51	0.614

Post-Hoc Contrasts (Enterococci)

Each antibiotic group compared to Saline

	Estimate	SE	DF	Lower 95% CI	Upper 95% CI	t-ratio	p-value
**1 Day Post-Treatment**							
1 Day - Saline	0.09	1.42	32	−3.66	3.85	0.07	0.947
3 Day - Saline	−3.04	1.57	24	−7.30	1.21	−1.93	0.195
6 Day - Saline	−2.98	1.30	42	−6.36	0.41	−2.29	0.108
10 Day - Saline	−2.05	1.26	34	−5.36	1.26	−1.63	0.224
**3 Day Post-Treatment**							
1 Day - Saline	−0.83	1.42	32	−4.58	2.92	−0.58	1.000
3 Day - Saline	−1.90	1.57	24	−6.15	2.35	−1.21	0.717
6 Day - Saline	−2.78	1.30	42	−6.17	0.61	−2.14	0.152
10 Day - Saline	0.46	1.26	34	−2.86	3.77	0.36	1.000
**7 Day Post-Treatment**							
1 Day - Saline	−1.71	1.42	32	−5.46	2.05	−1.20	0.713
3 Day - Saline	−4.07	1.57	24	−8.33	0.18	−2.59	0.065
6 Day - Saline	−1.36	1.30	42	−4.75	2.03	−1.05	0.713
10 Day - Saline	−0.18	1.26	34	−3.50	3.13	−0.15	0.884
**14 Day Post-Treatment**							
1 Day - Saline	0.38	1.42	32	−3.37	4.13	0.27	1.000
3 Day - Saline	2.36	1.57	24	−1.89	6.61	1.50	0.440
6 Day - Saline	0.71	1.30	42	−2.67	4.10	0.55	1.000
10 Day - Saline	2.70	1.26	34	−0.61	6.02	2.15	0.155
**22 Day Post-Treatment**							
1 Day - Saline	−1.74	1.42	32	−5.49	2.01	−1.23	0.912
3 Day - Saline	−0.47	1.57	24	−4.72	3.79	−0.30	1.000
6 Day - Saline	−1.55	1.30	42	−4.93	1.84	−1.19	0.912
10 Day - Saline	0.86	1.26	34	−2.46	4.17	0.68	1.000

To evaluate how antibiotic duration affects enterococci colonization dynamics, mice were pre-treated with 1, 3, 6, or 10 days antibiotics and then stool samples were collected to evaluate *Enterococcus* CFU. The saline group served as a control and maintained high CFU levels throughout this group. Antibiotic treated groups experienced initial depletion followed by variable recovery trajectories. Dunnett-pairwise comparisons were performed at post-treatment point to assess statistical differences from saline. One day after stopping antibiotic treatment, there was no groups that were significantly significant from the saline controls, but all groups (besides the 1-day group) had lower concentrations of *enterococci* within their stool. The greatest reductions of *enterococci* were in the 3 day (δ = -3.04, 95% CI [-7.30, 1.21]; $p = 0.196$), 6 day (δ = -2.98, 95% CI [-6.36, 0.41]; $p = 0.108$) and 10 day (δ = -2.05, 95% CI [-5.37, 1.27]; $p = 0.225$) antibiotic groups. The 1-day antibiotic had nearly identical $\log_{10}$ CFU concentrations compared to the saline (δ = 0.09, 95% CI [-3.66, 3.85]; $p = 0.947$). All of the groups continued to remain statistically non-significant (all $p > 0.152$) into day 3, with the magnitudes of differences for all the groups decreasing. Seven days after treatment, no comparison reached statistical significance, though the 3 day group trended toward significance (δ = -4.07;

95% CI [-8.33, 0.19]; $p = 0.065$). This suggest that a moderate suppressive effect with a 3 day treatment course of antibiotics. Non-significant differences from the controls continued into 14 days and 22 days after treatment with antibiotics. By day 22, nearly all antibiotic groups were indistinguishable from saline groups (all $p > 0.912$).

Despite initial depletion enterococci recovered rapidly across all antibiotic groups, with no statistically significant differences from saline controls at any time point following post hoc adjustment. While brief suppression was observed in intermediate duration regimens (particularly 3-day treatments) these effects were short-lasting. The findings highlight the minimal impact of antibiotics on *enterococci* and suggest that antibiotic-induced disruption does not meaningfully disrupt gut flora of *enterococci.*

Overall Group Comparisons for Enerococci

Comparisons of overall differences for antibiotics group in colonization

	Estimate	SE	DF	Lower 95% CI	Upper 95% CI	t-ratio	p-value
Saline - 1 Day	0.76	0.98	13	−2.52	4.04	0.78	1.000
Saline - 3 Day	1.43	1.05	13	−2.14	4.99	1.36	1.000
Saline - 6 Day	1.59	0.89	13	−1.42	4.60	1.78	0.872
Saline - 10 Day	−0.36	0.88	12	−3.40	2.68	−0.40	1.000
1 Day - 3 Day	0.66	1.10	20	−2.82	4.14	0.60	1.000
1 Day - 6 Day	0.83	0.95	24	−2.11	3.77	0.87	1.000
1 Day - 10 Day	−1.12	0.94	22	−4.06	1.82	−1.19	1.000
3 Day - 6 Day	0.16	1.03	21	−3.07	3.40	0.16	1.000
3 Day - 10 Day	−1.78	1.02	19	−5.02	1.46	−1.75	0.872
6 Day - 10 Day	−1.95	0.85	24	−4.58	0.69	−2.28	0.318

To assess whether the duration of antibiotic pretreatment had a sustained impact on *Enterococcus* colonization, pairwise comparisons between all treatment and control groups were conducted, averaging $\log_{10}$ CFU levels across all post-treatment time points. These comparisons would help determine whether any antibiotic regimen conferred lasting suppression or enhancement of *enterococci* colonization. The post-hoc test found no statistically significant differences between any pair of groups (all $p > 0.319$). The comparison with the largest difference was between the 6-day and 1- day groups with the 6-day having an average difference of -1.95 $\log_{10}$ CFU/g lower (95% CI [-4.58, 0.69]; $p = 0.319$) *enterococci.* None of the antibiotic groups were significantly different from the saline control (all $p > 0.872$).

These findings reinforce the conclusion that antibiotic duration had no meaningful or lasting effect on overall enterococci colonization. While trasient reductions were observed at specific time points, these differences did not persist when CFU levels were aggregated across the experimental study period.

1.10.3 Conclusions

Unlike gram-negative bacteria, *Enterococcus* populations demonstrate strong resilience to antibiotic perturbation, rebounding to stable levels regardless of pretreatment duration. The absence of any significant difference in overall colonization suggest that Enterococcus can rapidly and consistently recolonize the gut following antibiotic exposure, regardless of regimen length.
