# Supplementary material for: Longer durations of piperacillin/tazobactam treatment cause more prolonged alteration of colonization resistance in mice
Source: PLoS One. 2026 Jun 1;21(6):e0350031. doi: 10.1371/journal.pone.0350031 (PMC13225349; doi:10.1371/journal.pone.0350031)
Supplement: Attachment — Submitted filename: Review_Donskey_4.9.2026.docx [file pone.0350031.s005.docx]

Using a mouse model, the authors examined how the duration of broad-spectrum antibiotic treatment affects colonization resistance. These extensive datasets serve as a foundation for future research aimed at reducing the negative consequences of antibiotic use. However, the experiments are not cohesively integrated. The changes of indigenous enterococci and gram-negative bacteria at 0, 1, 3, 7, 14, and 22 days post-treatment were presented in Figure 4. Results from one experiment should provide data that inform, refine, and necessitate the next experiments. Data presented in Figures 5 and 6 illustrate the impact of piperacillin/tazobactam treatment on the gut microbiome 0 and 14 days post-treatment. The effects of antibiotics treatment on the gut microbiota's ability to prevent pathogen invasion was assessed using cecal contents obtained 6 days post treatment. Yet, pathogen challenge (VRE and *Klebsiella pneumoniae*) was conducted at 10 and 24 days post-treatment. Despite the lack of data regarding microbiota changes or in vitro colonization resistance at those time points.

Translating these findings into clinical practice requires careful consideration of the interspecies differences between humans and mice. Because mice have a much faster metabolism and higher drug clearance rates than humans, a 3-day treatment in mice might translate to a 7- to 14-day human course. However, it is not well understood how antibiotic treatment effects on gut microbiota and colonization resistance differ between humans and mice. A more thorough discussion regarding the translation of study findings into clinical practice is required.

Supplemental data is unlinked to the text.

Minor points:

Lines 34-36; Conclusion of this study is not clear.

Lines 63-65; Please clarify what has not been determined yet, even though there are many reports that describe short courses of antibiotics still can disrupt colonization resistance [17-24]. Is it about the amount of disruption, the effects of broad-spectrum antibiotics, or something else?

Lines 80-82, lines 86-89 are not necessary information for the manuscript. Because there are no adverse events during the study (line 89-92).

Line 107, What is the MIC of C68? 1250 ug/mL or >1250 ug/ml? Please check.

Line 109, carbapenemase-producing Klebsiella pneumoniae is generally resistant to piperacillin/tazobactam. So, MIC of Kp VA367 would be >128 ug/ml. Please check.

Line 199; where are the data from day 3 stool specimens?

Lines 228-233; Please correct the subject word. The ‘mice’ were not correct subject for these sentences.

Line 341; The findings at Day 14 (3-day treatment group) are inconsistent with the data shown in Figure 4(B). Please discuss.

Lines 351-353: These pieces of information are not in Figure 6.

Lines 356-358; Are there any data to show the gut microbiota after aztreonam treatment?
